# Photodegradation reveals that singlet energy transfer impedes energy-gradient-driven singlet fission in polyacene blends†

**DOI:** 10.1039/d4sc06702a

**Published:** 2025-01-14

**Authors:** Alexandra N. Stuart, Jessica M. de la Perrelle, David M. Huang, Tak W. Kee

**Affiliations:** a Department of Chemistry, The University of Adelaide Adelaide South Australia 5005 Australia alexandra.stuart@adelaide.edu.au david.huang@adelaide.edu.au tak.kee@adelaide.edu.au

## Abstract

Singlet fission (SF) is a process that is potentially beneficial for photovoltaics by producing two triplet excitons from a single photon, but its application is often hindered by the inability to effectively separate the resultant triplet excitons. It has been proposed that an energy gradient can assist in separating triplet excitons through triplet energy transfer between chromophores of different triplet energies, but this approach has only been studied in solution and the efficacy of this strategy in the solid state is under explored. Here, we investigate energy-gradient-driven SF in a disordered solid state, in the form of suspensions of 5,12-bis(triisopropylsilylethnyl)tetracene:6,13-bis(triisopropylsilylethnyl)pentance (TIPS-Tn:TIPS-Pn) blend nanoparticles (NPs). Rather than using more conventional techniques such as ultrafast (sub-nanosecond) spectroscopy, we study the photophysics in these NPs through monitoring their photodegradation. TIPS-Tn photodegrades rapidly in neat NPs, but this photodegradation is suppressed upon the addition of TIPS-Pn, indicating a decrease in the TIPS-Tn triplet population. By modeling the photodegradation over a timescale of minutes to hours, we are able to reveal details of processes on the ultrafast timescale. We show that triplet energy transfer occurs from TIPS-Tn to TIPS-Pn, leading to slower photodegradation for TIPS-Tn, and faster photodegradation for TIPS-Pn. However, modeling additionally indicates that singlet energy transfer from TIPS-Tn to TIPS-Pn also occurs, and in fact acts to reduce the efficiency of TIPS-Tn SF. Hence, in this particular system, the energy gradient impedes SF, rather than assisting it. These findings indicate that chromophore pairs must be carefully selected to switch off singlet energy transfer for the energy-gradient approach to be effective in enhancing SF.

## Abstract 2

Employing an energy gradient between singlet-fission chromophores with different triplet energies to assist triplet-exciton separation can instead hinder singlet fission if singlet energy transfer outcompetes triplet energy transfer.

## Graphical abstract

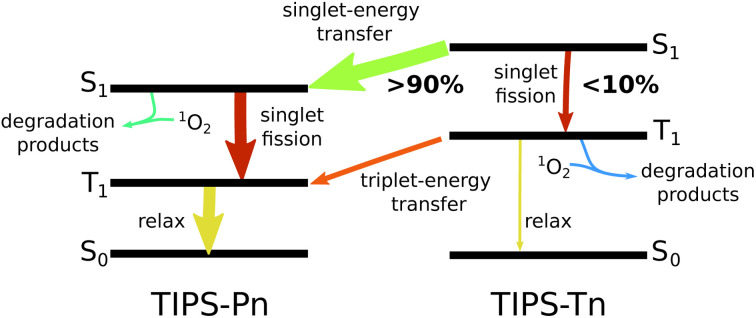

## 1 Introduction

Singlet fission (SF) is a process in which a photoexcited singlet exciton is converted to a pair of triplet excitons with overall singlet character on neighboring chromophores. This process is spin-allowed and can occur on ultrafast (sub-nanosecond) timescales, allowing SF to compete with other loss pathways. SF has grown in popularity for its potential to increase the efficiency of solar cells, specifically by addressing the thermal energy losses from high-energy photons.^1–3^ Typically, the excess energy of photons above the band gap of a solar cell is lost due to thermal relaxation, but including a SF layer in a solar cell allows these high-energy photons to instead produce two excitons, doubling the photocurrent in the high-energy region of the solar spectrum without reducing the voltage of the cell. While this application is promising, attempts to incorporate SF in solar cells have yet to show substantial enhancements,^4–7^ indicating a need to improve the understanding of SF and how to best implement it in devices.

One complexity of SF is the involvement of an intermediate species, the spin-correlated triplet-pair intermediate with overall singlet character, denoted ^1^(TT).^8–11^ Including this intermediate, SF can be described by the equation

1 S_0_ + S_1_ ⇌ ^1^(TT) ⇌ T_1_ + T_1_.

It has been demonstrated in several systems that this intermediate can fail to separate, and instead recombine to S_1_, or decay directly to the ground state.^12–17^ For SF to be of any benefit in solar cells, the resultant triplet excitons should be able to separate and be harvested for charge generation. It is undesirable for the free triplets to recombine to singlets (*e.g. via* triplet fusion or triplet–triplet annihilation), or for the system to become “stuck” in the triplet-pair state, only to decay or recombine. Another main requirement for SF is that the efficiency of the process should be high, *i.e.* two triplet excitons should be formed for every singlet exciton excited, giving an efficiency or triplet yield close to 200%. But achieving both high efficiency and good triplet separability simultaneously is challenging. Often the conditions for efficient SF (or at least an efficient first step of SF) are different to those that allow triplets to easily separate, leading to the major question of how to balance the formation of triplet excitons (or triplet-pair intermediates) with the ability to separate and harvest them.^15,18–23^

One approach that has been proposed to address these conflicting requirements is to design systems with an energy gradient that can channel triplets away from each other. This design principle was demonstrated by Pun *et al.* and uses two different chromophores with different triplet energies to create an energetic landscape that drives spatial separation.^24^ Pun *et al.* demonstrated this strategy by synthesizing oligomers with tetracene chromophores in the center, and pentacenes on the ends. After excitation, the central tetracenes undergo SF to give triplet excitons or triplet-pair intermediates. The terminal pentacenes have a lower triplet energy, which gives the resulting triplets (or triplet pairs) the energetic drive to transfer onto the pentacenes, helping them to separate, as well as making their recombination energetically uphill. In other words, the lower energy of the pentacenes acts to funnel the triplets away from each other. The results of this study were encouraging, producing long-lived triplet excitons at high yields. However, the molecules were only studied in solution and the tetracene and pentacene moieties were covalently bound in a highly specific arrangement. For triplet excitons to be harvested for practical application, these oligomers would ultimately need to be in the solid state. The question arises of how an energy-gradient design would fare in the solid state, in which other molecular arrangements are sampled, and alternative competing pathways for excitons become available. The efficacy of an energy-gradient design in a disordered solid state, in which several different molecular arrangements are sampled, may be a particular challenge for this design principle. Zeiser *et al.* previously studied crystalline blend films of tetracene and pentacene, with a focus on investigating singlet heterofission (SF to triplet excitons on chemically distinct chromophores), and codeposited the two molecules to form a solid solution.^25^ While they demonstrated that heterofission can occur in these blend films when pentacene was excited, they did not observe heterofission when tetracene was excited, nor any triplet-energy transfer (TET) from tetracene to pentacene. Instead, these films predominantly exhibited singlet-energy transfer (SET) from tetracene to pentacene, which outcompeted any tetracene SF. Although investigating the effectiveness of the energy-gradient approach was not the goal of this study, this finding further suggests that a highly specific chromophore arrangement may be necessary for an energy gradient to be beneficial for SF. If such an arrangement is absent in a crystalline form, then an amorphous morphology that samples many different arrangements may in fact be more advantageous for the solid state.

In another study on the electrically detected magnetic resonance of triplets, crystalline tetracene films were doped with a small percentage (0.1%) of pentacenes to detect the spin polarization of isolated pentacene triplets.^26^ Triplet excitons were detected on isolated pentacenes after tetracene was excited, indicating that in this system SF on tetracene was able to outcompete singlet-energy transfer to some extent. However, the relative amounts of SF, SET, and TET were not quantified in this study, nor by Zeiser *et al.*, making it difficult to compare or extract general conclusions.

**Fig. 1:**
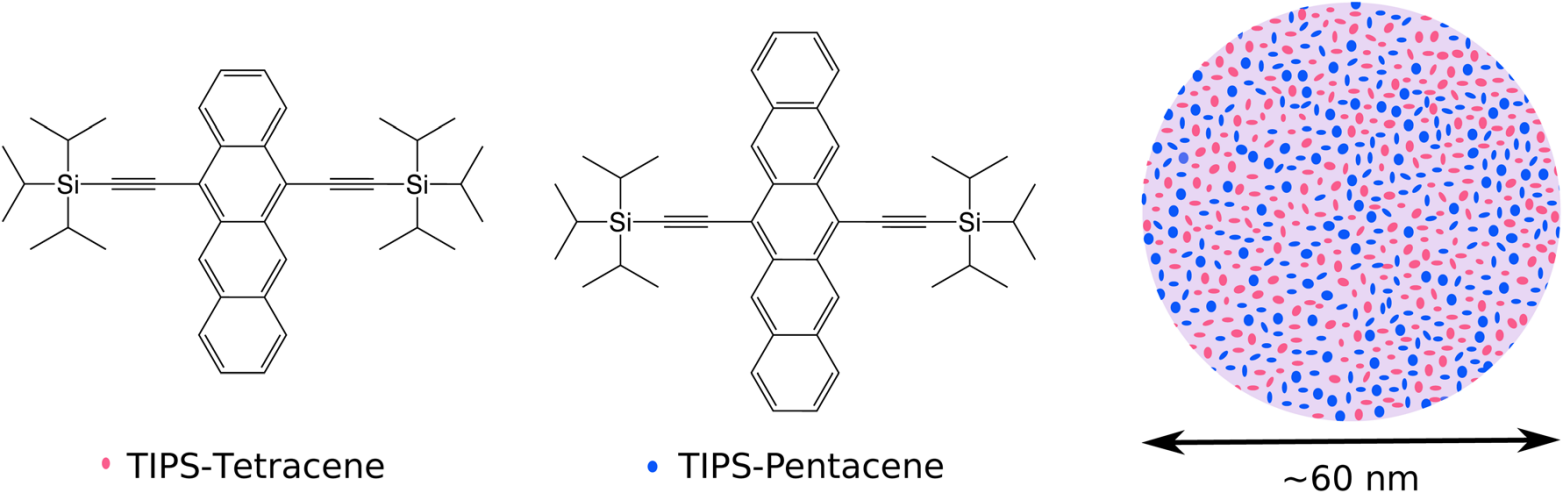
Molecular structures of TIPS-Tn and TIPS-Pn, and a cross-section schematic of a TIPS-Tn:TIPS-Pn blend nanoparticle, in which TIPS-Tn and TIPS-Pn are represented in red and blue, respectively.

In this study, we investigate the effect of an energy gradient on SF in a disordered solid state through the medium of aqueous nanoparticle (NP) suspensions. NPs are a convenient model of the solid state that allow disordered and well blended morphologies, and mitigate many of the challenges associated with other solid-state media, such as poor transparency, light scattering, photodegradation, and artifacts from heating,^12,27–30^ although thermal artifacts may not be completely eliminated.^31^ We use the same chromophores as Pun *et al.*: tetracene as the high triplet-energy chromophore, and pentacene as the low triplet-energy chromophore. In our system, these chromophores are not covalently linked, but instead blended in an amorphous manner at various mass ratios, as depicted in Fig. 1. As with Pun *et al.*, we use the triisopropylsilylethnyl (TIPS) substituted form of these chromophores, TIPS-pentacene (TIPS-Pn) and TIPS-tetracene (TIPS-Tn), to aid with solution processibility. We primarily aim to discover the extent to which triplets generated from SF on TIPS-Tn are transferred to TIPS-Pn in the NPs, and thus the feasibility of an energy gradient assisting SF in this system. Conventionally, processes such as SF or energy transfer in the solid state would be studied on a femto or picosecond timescale using transient absorption spectroscopy and/or ultrafast emission spectroscopy. But these techniques can be challenging for polyacenes because of their low photostability.^31–34^ TIPS-Tn in particular photooxidizes rapidly in the solid state. This issue can be addressed somewhat for studies on films by measuring them under vacuum or in an inert environment, or for studies on solutions by purging with argon or nitrogen gas. However, for the NPs suspended in water these methods become either impractical or ineffective, and other complex methods have to be used to avoid degradation.^34–36^ Hence in this study, rather than using ultrafast spectroscopy, we study the NPs through characterizing their photodegradation. As we show, the photodegradation of the NPs over multiple hours can reveal a surprising level of detail on phenomena occurring on ultrafast timescales. By modeling the photodegradation, we show that TET from TIPS-Tn to TIPS-Pn does occur in these systems, and also leads to the suppression of TIPS-Tn photodegradation. However, modeling also indicates that singlet energy transfer from TIPS-Tn to TIPS-Pn also occurs, and in fact acts to reduce the efficiency of SF in TIPS-Tn. Our results suggest that specific and precise molecular arrangements will be necessary for an energy gradient to be beneficial to SF, or otherwise judicious choice of chromophore blends with energetics that can avoid singlet energy transfer.

## 2 Results and discussion

**Fig. 2:**
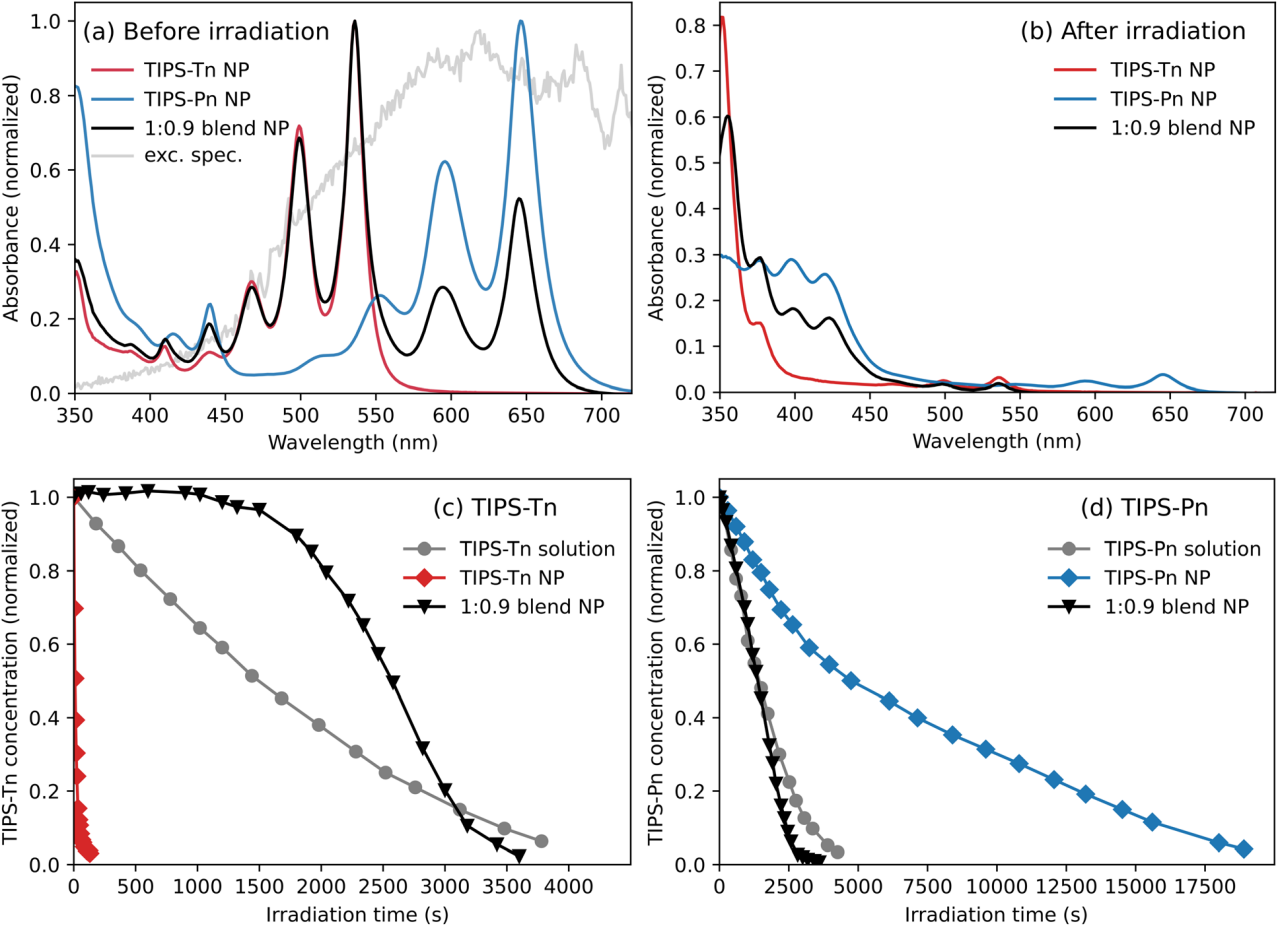
Steady-state absorption of neat TIPS-Tn, neat TIPS-Pn, and 1 : 0.9 TIPS-Tn:TIPS-Pn blend NPs suspended in water (a) before irradiation and (b) after 2.2, 130, and 60 min of irradiation for TIPS-Tn, TIPS-Pn, and the 1 : 0.9 blend, respectively. Photodegradation monitored as concentration *vs.* irradiation time of (c) TIPS-Tn for a 12 μM TIPS-Tn toluene solution, neat TIPS-Tn NPs, and 1 : 0.9 blend NPs; and (d) TIPS-Pn for a 28 μM TIPS-Pn toluene solution, neat TIPS-Pn NPs, and the same 1 : 0.9 blend NPs. The excitation spectrum used for photodegradation is shown in gray in (a).

TIPS-Tn:TIPS-Pn blend NPs were prepared with various mass ratios as described in the ESI (ESI Section S1.1).† The size of the NPs was kept between 60 and 90 nm, as determined from dynamic light scattering experiments (ESI Section S1.2†). The steady-state absorption spectra of neat TIPS-Tn NPs, neat TIPS-Pn NPs, and blend NPs with a 1 : 0.9 mass ratio are given in Fig. 2a. NPs were prepared using a reprecipitation technique, which we have previously shown forms polyacene NPs with amorphous morphologies.^12,27^ The spectra correspondingly resemble those of TIPS-Pn and TIPS-Tn in solution (Fig. S3†), and lack the strong electronic coupling features observed in crystalline phases.^25,27,37^ To study the photodegradation of the NPs, the suspensions were first saturated with oxygen and then irradiated with a known light source, with their absorption monitored over irradiation time. The details of the degradation procedure, including the light source, are given in the ESI Section S1.3.†Fig. 2a shows the samples before irradiation, and Fig. 2b shows the absorbance of the photodegraded samples, after 130 s for TIPS-Tn NPs, 130 min for TIPS-Pn NPs, and 60 min for the 1 : 0.9 TIPS-Tn:TIPS-Pn blend NPs. Some amounts of TIPS-Pn and TIPS-Tn still remain in Fig. 2b, but new absorption features have emerged in the region from 350 to 450 nm. These features correspond to endoperoxides, which are the primary product of the photooxidation of TIPS-Pn and TIPS-Tn.^32,38^ Specifically, for TIPS-Tn the absorption could be due to either or both of 5,12- or 6,11-endoperoxides, which form with similar selectivity,^32,39^ and for TIPS-Pn these features have been reported as the 5,14-endoperoxide.^32,33^ Apart from the additional light scattering observed in the NP spectra, the same absorption features are observed for both solution and NP spectra after irradiation (Fig. S3†), indicating that predominantly the same photodegradation products are formed in both phases. The photodegradation of TIPS-Tn and TIPS-Pn primarily occurs through a reaction with singlet oxygen (^1^O_2_), which is sensitized by the photoexcited states of these polyacenes.^34^ We have recently characterized the photodegradation of TIPS-Pn and TIPS-Tn in solution in detail, and showed that both S_1_ and T_1_ excitons of these chromophores are able to sensitize ^1^O_2_.^34^^1^O_2_ subsequently reacts with TIPS-Pn primarily in the S_1_ state, and TIPS-Tn in the T_1_ state, to give the product endoperoxides.^34^

Fig. 2c and d show the photodegradation of TIPS-Tn and TIPS-Pn, monitored as concentration *vs.* irradiation time. The concentrations are determined from absorption spectra as described in ESI Section S3.† In addition to the NP photodegradation, the photodegradation of TIPS-Pn and TIPS-Tn in toluene solutions is also shown. TIPS-Tn photodegrades to the endoperoxide products quickly in the neat NP suspension (*i.e.* in the solid state), but the photodegradation of TIPS-Tn in solution is substantially slower, as shown in Fig. 2c. A key difference between TIPS-Tn in solution and in NP form is the rate of SF. In the 12 μM TIPS-Tn solution, SF is diffusion limited and relatively slow, so triplets are instead predominantly formed through sensitization by oxygen over a timescale of tens of nanoseconds.^34^ In the solid state, SF is rapid, and triplets are formed within picoseconds. The faster degradation of TIPS-Tn in NP form hence correlates with a larger triplet population. For TIPS-Tn, this behavior is both due to the higher efficiency of TIPS-Tn triplets to sensitize singlet oxygen, and because the predominant degradation pathway occurs through the T_1_ state.^34^

While the photostability of TIPS-Tn in neat TIPS-Tn NPs is very low, a substantial improvement in the stability is observed upon blending with TIPS-Pn. Fig. 2c shows that the TIPS-Tn component in the 1 : 0.9 TIPS-Tn : TIPS-Pn blend NPs lasts significantly longer than in the neat TIPS-Tn NPs. If the photodegradation of TIPS-Tn is facilitated by the triplet state, then slower photodegradation in the blend NPs suggests a smaller TIPS-Tn triplet population. This observation could indicate fewer TIPS-Tn triplet excitons formed in the blend NPs due to a lower rate of SF, potentially because of dilution of TIPS-Tn chromophores by TIPS-Pn. Alternatively, slower photodegradation in the blend NPs could indicate that an alternative pathway has become available for the TIPS-Tn triplet excitons that competes with photodegradation, such as triplet energy transfer to TIPS-Pn.

For TIPS-Pn, photodegradation predominantly occurs through the excited singlet state,^34^ and SF acts as a competing pathway for oxygen sensitization and photodegradation, rather than facilitating it as with TIPS-Tn. Photodegradation is hence slower in NP form than in solution, as SF is faster in the NPs and the singlet exciton population is lower. In the 1 : 0.9 NP blend, the TIPS-Pn component degrades faster than in neat TIPS-Pn NPs, and even faster than TIPS-Pn in solution. This behavior could be partially due to a lower rate of SF in the blend due to dilution of the TIPS-Pn chromophores by TIPS-Tn, but it seems unlikely that the accelerated degradation could entirely be explained by this effect. In past studies of TIPS-Pn:polymethyl methacrylate (PMMA) composite NPs, TIPS-Pn still exhibited SF on the picosecond timescale even when diluted by 10 times the amount of PMMA.^12^ So in a 1 : 0.9 TIPS-Pn : TIPS-Tn blend, we expect SF to still be substantially faster than in solution. Alternatively, energy transfer from TIPS-Tn to TIPS-Pn could serve to increase the photodegradation rate of TIPS-Pn by increasing the population of excited states, and hence the population of states that interact with oxygen. Note that this will not affect the balance of S_1_ and T_1_ states in TIPS-Pn, but rather increase the amount of S_1_ and T_1_ states relative to S_0_. Hence energy transfer to TIPS-Pn would have an analogous effect to increasing the excitation power and rate constant experienced by TIPS-Pn.

**Fig. 3:**
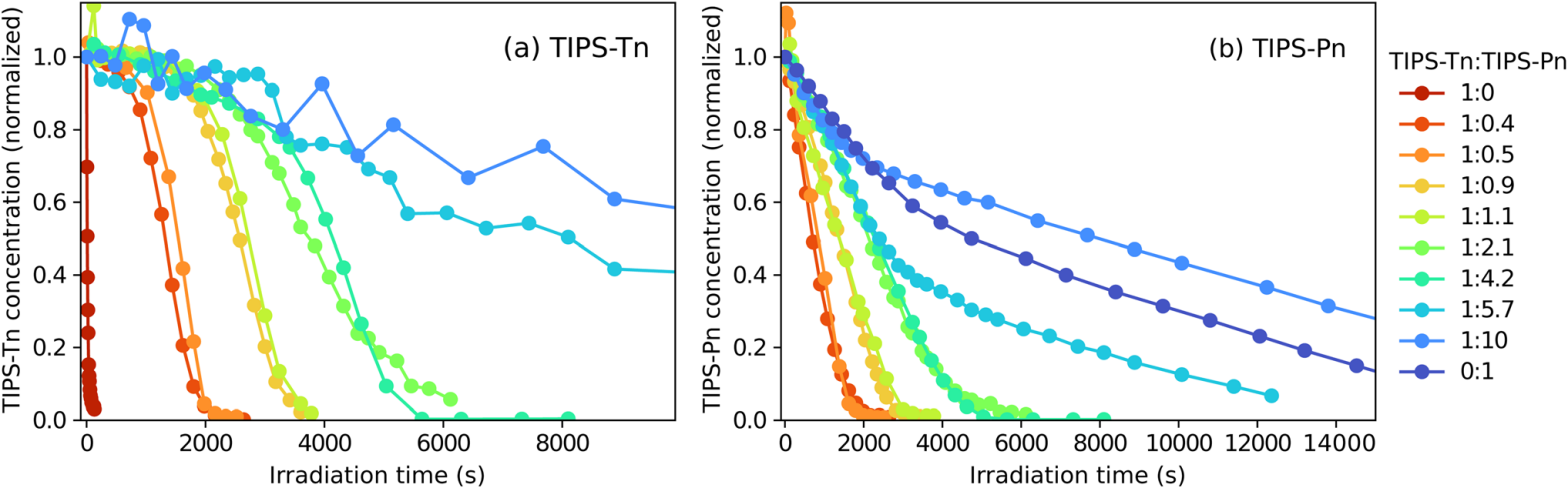
Photodegradation of TIPS-Tn : TIPS-Pn NPs at various mass ratios: concentration of (a) TIPS-Tn component and (b) TIPS-Pn component *vs.* irradiation. Concentrations are normalized to the initial data point.

To further demonstrate the relationship between photodegradation and the presence of TIPS-Tn or TIPS-Pn, we additionally irradiated a series of blend NPs of different TIPS-Pn : TIPS-Tn mass ratios, as shown in Fig. 3. A clear trend can be observed in the photodegradation of both chromophores. For TIPS-Tn, as the proportion of TIPS-Pn in the NP is increased, the degradation becomes slower. In the NPs with the highest proportion of TIPS-Pn (1 : 10 TIPS-Tn : TIPS-Pn) the photodegradation of TIPS-Tn is roughly 200 times slower than in the neat TIPS-Tn (1 : 0 TIPS-Tn : TIPS-Pn) NPs. Conversely, the photodegradation of TIPS-Pn in the NPs becomes faster with an increasing proportion of TIPS-Tn. There also appears to be a relationship between the shape of the decays in Fig. 3 for the two chromophores at each mass ratio. The TIPS-Tn concentration in the NP is relatively constant until a particular irradiation time, at which point it begins to rapidly decay. This turning point roughly corresponds to the irradiation time at which the TIPS-Pn concentration reaches zero. This result demonstrates that the presence of TIPS-Pn suppresses TIPS-Tn photodegradation, and only when the concentration of TIPS-Pn becomes close to zero does the TIPS-Tn begin to decay similarly to the neat NPs. This behavior is indicative of exciton transfer from TIPS-Tn to TIPS-Pn, rather than simply a decrease in the rate of TIPS-Tn SF, since both TIPS-Pn and its endoperoxides will dilute TIPS-Tn and decrease the SF rate, so dilution alone would not lead to this behavior.

It is evident that the photodegradation of TIPS-Tn and TIPS-Pn NPs is governed by both SF and exciton transfer between the two chromophores. In particular, the dependence of the TIPS-Tn photodegradation on the TIPS-Pn concentration suggests that triplet energy transfer may be occurring. Modeling the degradation data will therefore clarify the extent of triplet energy transfer, and by extension the effect of the triplet energy gradient in this system.

### 2.1 Modeling

The photodegradation of TIPS-Tn and TIPS-Pn in each blend NP was modeled using a system of differential equations. We began by first fitting the photodegradation of the neat NPs (1 : 0 and 0 : 1 TIPS-Tn : TIPS-Pn) to obtain the rate constants of photodegradation for TIPS-Pn and TIPS-Tn from the S_1_ and T_1_ states, respectively. These rate constants could then be applied to the blend systems, and the blend NP photodegradation fit to determine the extent of energy transfer.

**Fig. 4:**
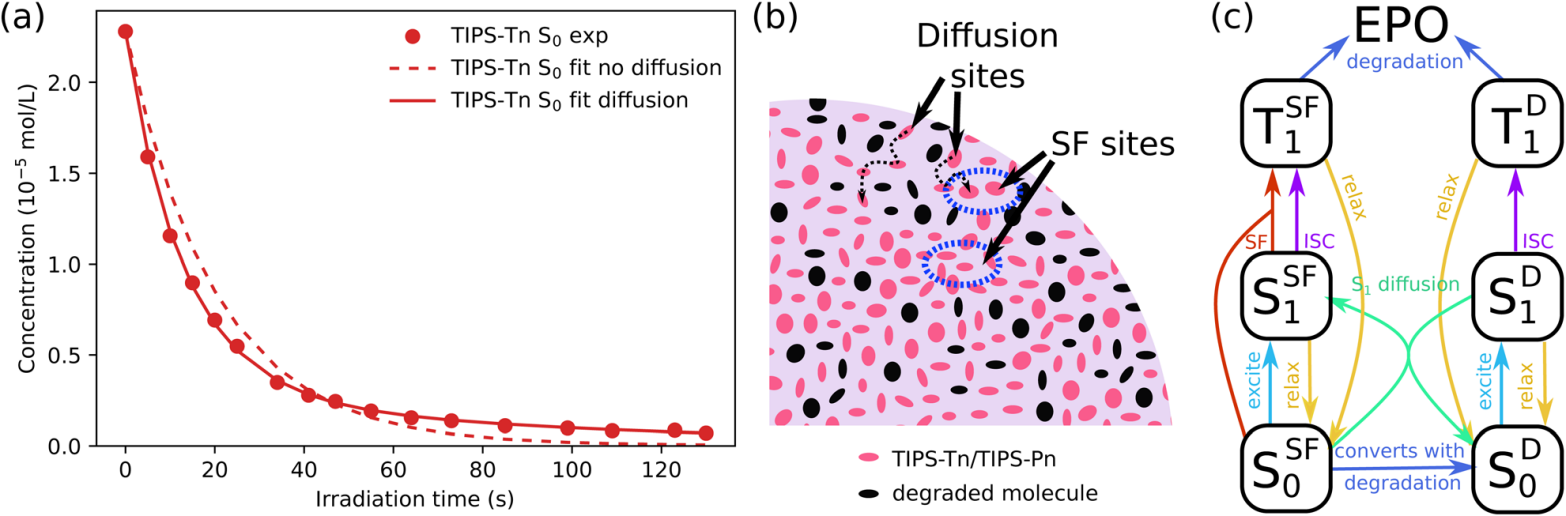
(a) Fits to the photodegradation of a sample of TIPS-Tn NPs with and without diffusion sites. Lack of exciton diffusion results in an overestimation of the photodegradation at late times. (b) Segment of a neat TIPS-Tn or TIPS-Pn NP illustrating how exciton diffusion becomes more significant as molecules in the NP photodegrade, which can be described by creating what we refer to as diffusion sites. (c) Photodegradation model including SF sites and diffusion sites used to fit the neat TIPS-Tn NP photodegradation data. EPO denotes endoperoxide.

Previously, we modeled the transient absorption and photodegradation data of neat TIPS-Tn and TIPS-Pn in solution in detail, but the photodegradation in NP form was only treated approximately.^34^ Here we fit the NP photodegradation more thoroughly. The main difference between models for solution and NP photodegradation can be demonstrated by first considering the photodegradation of neat TIPS-Tn NPs. As shown in detail in ESI Section S6.1,† we first fit the data for neat TIPS-Tn NPs using the same model as used for solution, but we found that the solution model overestimates the NP photodegradation at late times (Fig. 4a, dashed lines). This behavior can be explained by considering the differences between SF in the solid state and solution. In solution, SF is a result of collisions between molecules. As the number of molecules in solution is depleted through photodegradation, fewer collisions occur, resulting in less triplet formation through SF, and thus less degradation over time. This behavior is easily captured by describing SF with a second-order term in the kinetic model (see Section S6.1†), for which the rate of SF depends on the concentration of ground-state molecules and so becomes slower as the ground-state concentration decreases. In a solid-state system, SF occurs between fixed neighboring molecules rather than *via* collisions, so solely using the second-order term is insufficient to describe the deceleration of SF. As illustrated in Fig. 4b, as the population of TIPS-Tn decreases in the NP due to photodegradation, molecules lose their nearest neighbors and can no longer undergo SF. Excitons on these molecules either have to migrate away or remain trapped until they relax to the ground state or are sensitized to triplets by some other means (*e.g.* intersystem crossing (ISC)). Hence triplet formation, and subsequent photodegradation, slows down much more than the solution model can account for. It is thus necessary to include exciton diffusion in the photodegradation model for NPs.

*In lieu* of the necessary information to fit a full diffusion model, a common simpler method is to split molecules into two populations: molecules that are able to undergo SF, which we call SF sites and denote with the superscript SF (S^SF^_0/1_ or T^SF^_1_), and molecules that cannot, which we call diffusion sites and denote with the superscript D (S^D^_0/1_ or T^D^_1_). Excitons on diffusion sites are able to migrate to SF sites, as indicated in Fig. 4b (SF sites are circled in blue). This method of describing diffusion has previously been employed to model ultrafast TA data in amorphous pentacene and tetracene derivatives.^12,40^ We have also previously used more thorough Monte Carlo simulations to describe diffusion-limited SF in amorphous NPs, but this level of detail is unnecessary here, and conclusions from the Monte Carlo simulations were consistent with those of the simpler model.^41^ We considered the NPs before degradation to initially consist entirely of SF sites. As the NPs degrade, some SF sites become isolated and are converted into diffusion sites. The full model is shown in Fig. 4c, and the mathematical expression can be found in the ESI (Section S6.1.2†). By including diffusion sites, we were able to fit the photodegradation of TIPS-Tn well (Fig. 4a, full line). The model was fit individually to five different replicates of the photodegradation of neat TIPS-Tn NPs, and diffusion was found to improve the fit in each case. The fit quality was quantified by the root mean squared error (RMSE) of the fit of the normalized concentration, which across five samples is 0.039 without the diffusion population, and decreases to 0.009 with the diffusion population (RMSE_T_, eqn S23†). Using this model we found the average fitted degradation rate constant from the triplet state is (4.8 ± 3) × 10^6^ s^−1^ (time constant of 210 ± 15 ns), which is similar to that obtained from the photodegradation measured in toluene solution (time constant of 196 ns). Fitting also indicates that initially, for each degradation event on a SF site, there is a 22% chance that the remaining neighboring molecule will no longer be able to undergo SF and become classed as a diffusion site. This value is consistent with each molecule having 4–5 nearest neighbors.

While including diffusion sites is sufficient to describe neat TIPS-Tn NP photodegradation, describing neat TIPS-Pn photodegradation is more complicated, and is described in detail in ESI Section S6.2.† Briefly, two populations with different photodegradation rate constants from the singlet state are required. We attribute this to the packing in the TIPS-Pn NPs leading to some TIPS-Pn molecules being closer or more accessible to oxygen than others. For example, molecules at the surface of the NP may experience faster photodegradation than those in the NP core. Modeling suggests that 24% of molecules in the TIPS-Pn NPs have a photodegradation rate constant of (7 ± 1) × 10^7^ s^−1^ (time constant of 14 ns), and 76% of molecules had a slower degradation rate constant of (1.27 ± 0.03) × 10^7^ s^−1^ (time constant of 80 ns). It is unclear why multiple photodegradation rate constants are needed for TIPS-Pn, but not TIPS-Tn. Potentially because TIPS-Tn photodegrades through a different mechanism (*i.e.* through the triplet state), it is less sensitive to deviations in morphology or oxygen proximity. Regardless, given the photodegradation rate constants for TIPS-Tn and TIPS-Pn taken from the neat NPs systems, we were then able to fit the blend NP systems.

**Fig. 5:**
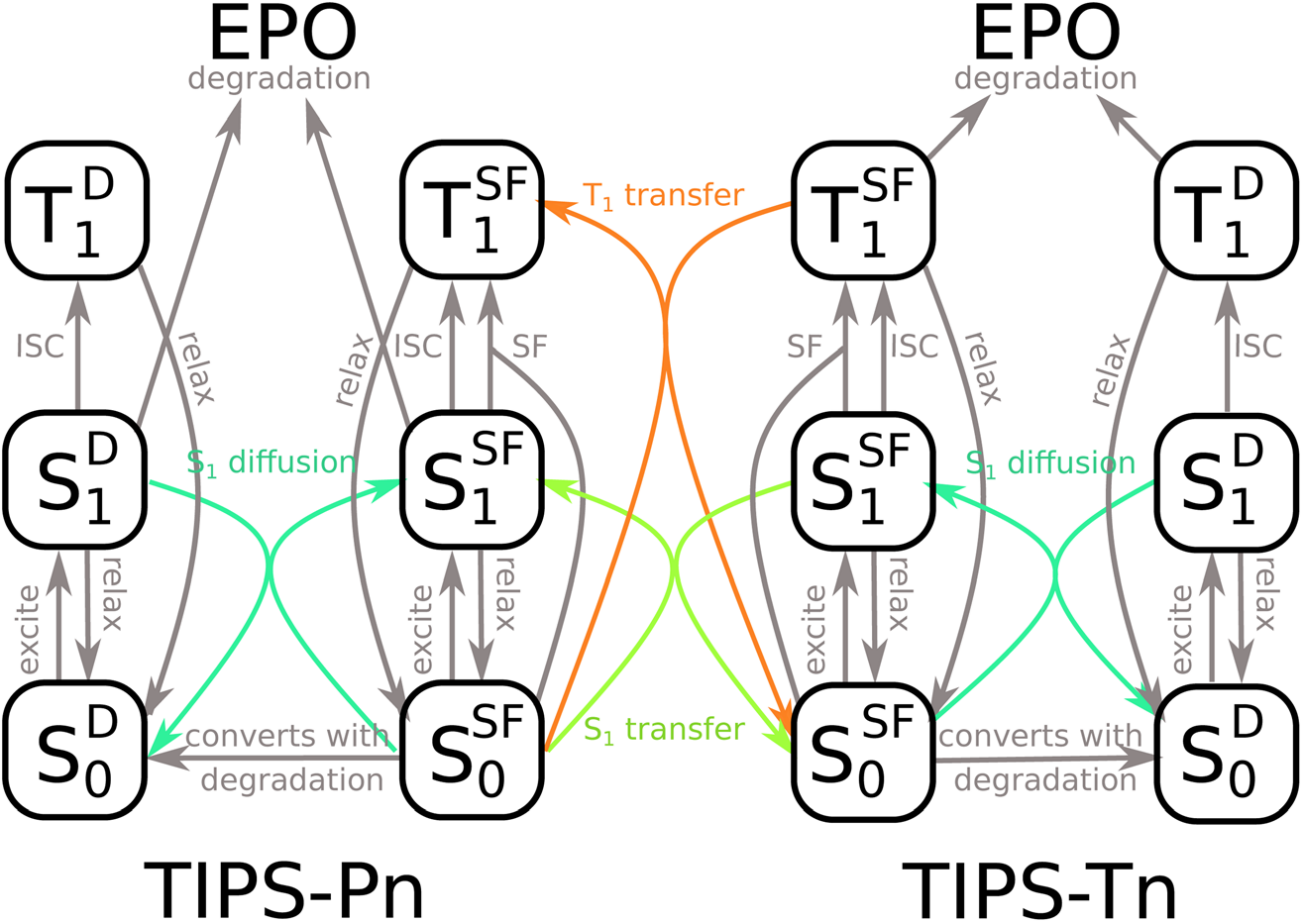
Model used to fit the photodegradation of TIPS-Tn:TIPS-Pn blend NPs. Processes in gray have known rate constants, taken from the neat systems. Only the processes in color are fit to the blend photodegradation data.

The model used to fit blend NP photodegradation is shown in Fig. 5, with the full mathematical model given in ESI Section S6.3.† There are two main differences between the blend model and the models used for neat NPs. The first is the addition of singlet and triplet energy transfer from TIPS-Tn to TIPS-Pn. The second is a non-zero initial population of diffusion sites. For neat NPs, we assume that all molecules begin as SF sites, and that diffusion sites are only created by the degradation of neighboring sites. In the blend NPs, TIPS-Tn molecules can be diluted by TIPS-Pn and *vice versa*, leading to sites at which SF cannot occur before the NPs have begun to photodegrade (singlet heterofission between TIPS-Tn and TIPS-Pn is possible, but we do not explicitly include this process in the model, as is discussed below). Therefore, as well as fitting the rate constants of energy transfer from TIPS-Tn to TIPS-Pn, we also fit the initial amount of diffusion sites and rate constants of diffusion. Note that though they occur through the same mechanism (*i.e.* Förster resonance energy transfer (FRET)), we exclusively use the term “diffusion” to describe energy transfer between molecules of the same type, while singlet exciton transfer between TIPS-Tn and TIPS-Pn is referred to as SET. Triplet diffusion (*i.e.* TET between molecules of the same type) is neglected, which we discuss below. This model incorporates many different processes, which may lead to concerns regarding the sensitivity of fitting it to the photodegradation data. However, we emphasize that only a few parameters are actually unknown. All the processes indicated in gray in Fig. 5 were constrained to values determined from the neat systems (Table S2†). The only parameters that were varied to fit the data are the rate constants of SET and TET, the rates of singlet exciton diffusion, and the proportion of diffusion sites present before photodegradation. Note that we fixed the rate constants of SF at SF sites in the blend NPs as identical to that of the neat NPs. Any decrease in rate or efficiency of SF as one type of molecule becomes diluted by the other was described as SF becoming more diffusion limited, rather than the rate constant of SF at SF sites changing. In other words, the identity of SF sites was the same in neat and blend NPs, and only the number of SF sites and the rate at which they are accessed were changed. This approach has previously been found to describe the effect of dilution on SF in amorphous solid-state systems well.^12,42^ This model also does not necessarily assume any particular morphology (*e.g.* phase separated or intermixed). If the NPs had large domains of TIPS-Tn or TIPS-Pn, the model would simply fit larger proportions of SF sites, and smaller proportions of exciton transfer from TIPS-Tn to TIPS-Pn. The blend fitting is explained in more detail in ESI Section S6.3.†

**Fig. 6:**
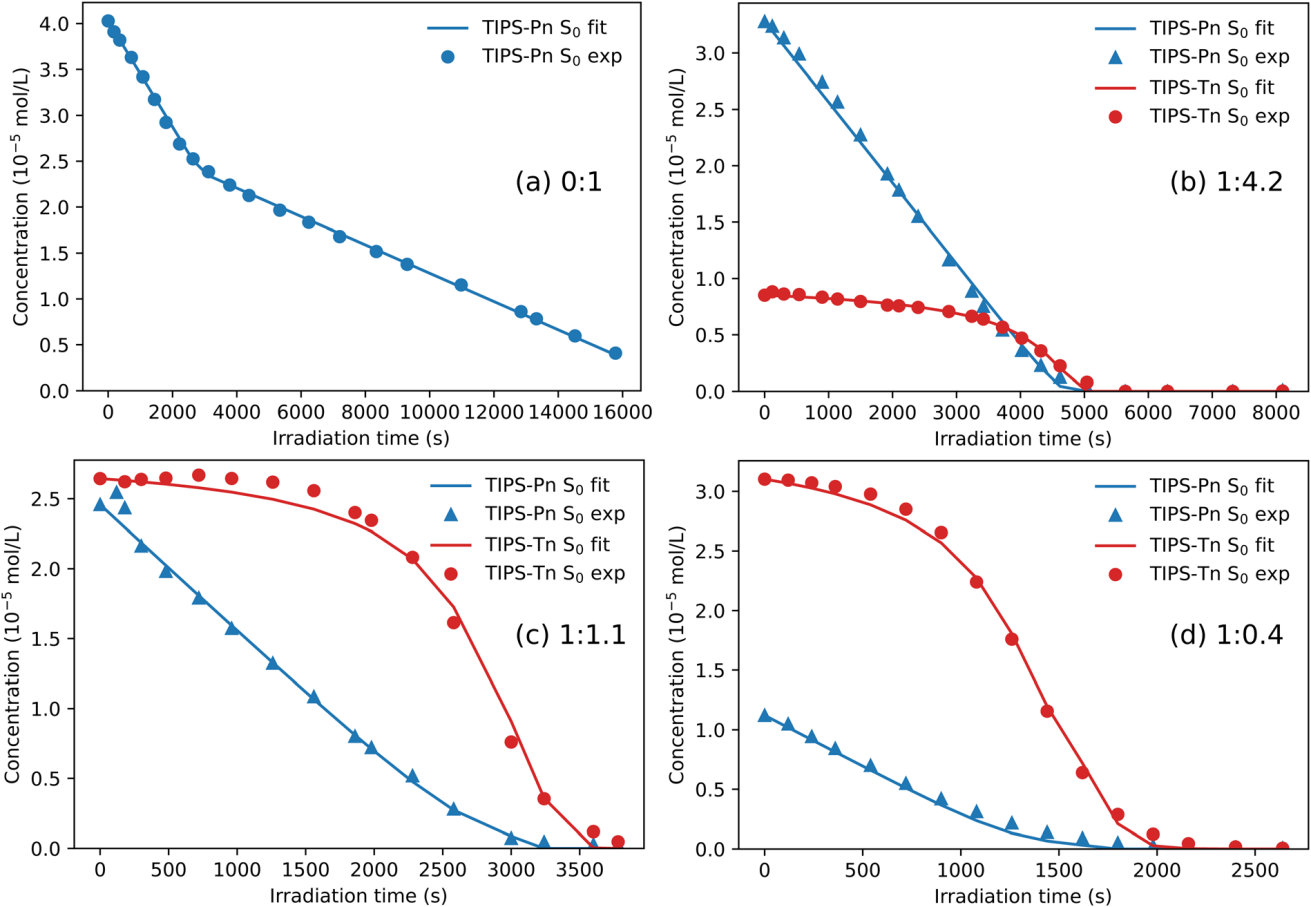
Fit to the photodegradation of blend TIPS-Tn : TIPS-Pn NPs with mass ratio (a) 0 : 1, (b) 1 : 4.2, (c) 1 : 1.1, and (d) 1 : 0.4, with the addition of SET and TET from TIPS-Tn to TIPS-Pn. Fits to additional blend ratios are given in the ESI (Fig. S27).† Note that different time axes are used due to the various time scales for photodegradation.

As discussed above, we consider two possible explanations for the increased photostability of TIPS-Tn in the blend NPs. The first is that the TIPS-Tn triplet population, and hence photodegradation, is reduced by SET or TET from TIPS-Tn to TIPS-Pn. The second is that SF in TIPS-Tn becomes less efficient as the TIPS-Tn molecules become diluted by increasing amounts of TIPS-Pn, leading to slower TIPS-Tn triplet production, and therefore slower photodegradation. The shape of the TIPS-Tn degradation curves makes the latter unlikely, but to verify this conclusion we initially fit the blend systems with SET and TET rate constants fixed to zero (Fig. S24†). This attempt resulted in poor fits to the photodegradation data, suggesting that TIPS-Tn to TIPS-Pn energy transfer indeed occurs. We next considered whether solely SET or solely TET could explain the data, and fit with either the rate constants of SET or the rate constants of TET fixed to zero (Fig. S25 and S26†). Even by only including one energy transfer process, the fits are substantially improved from a diffusion-only model, but they still show some discrepancies, particularly in the fit to the TIPS-Tn component of the NPs (Fig. S25 and S26†). The best fits to the data are achieved when both SET and TET are allowed, indicating both processes must be occurring in this system. The fit quality was quantified by the total RMSE across all the samples fit, which was lowest when both SET and TET are included (ESI Table S3†). Additionally, the rate constants of SET and TET are able to be fit sensitively and consistently as non-zero values, further indicating that both processes are non-negligible (ESI Table S5†). The resulting fits are shown for 1 : 0.4, 1 : 1.1, and 1 : 4.2 TIPS-Tn:TIPS-Pn NPs in Fig. 6, with the remaining blend ratios given in the ESI (Fig. S27†).

**Fig. 7:**
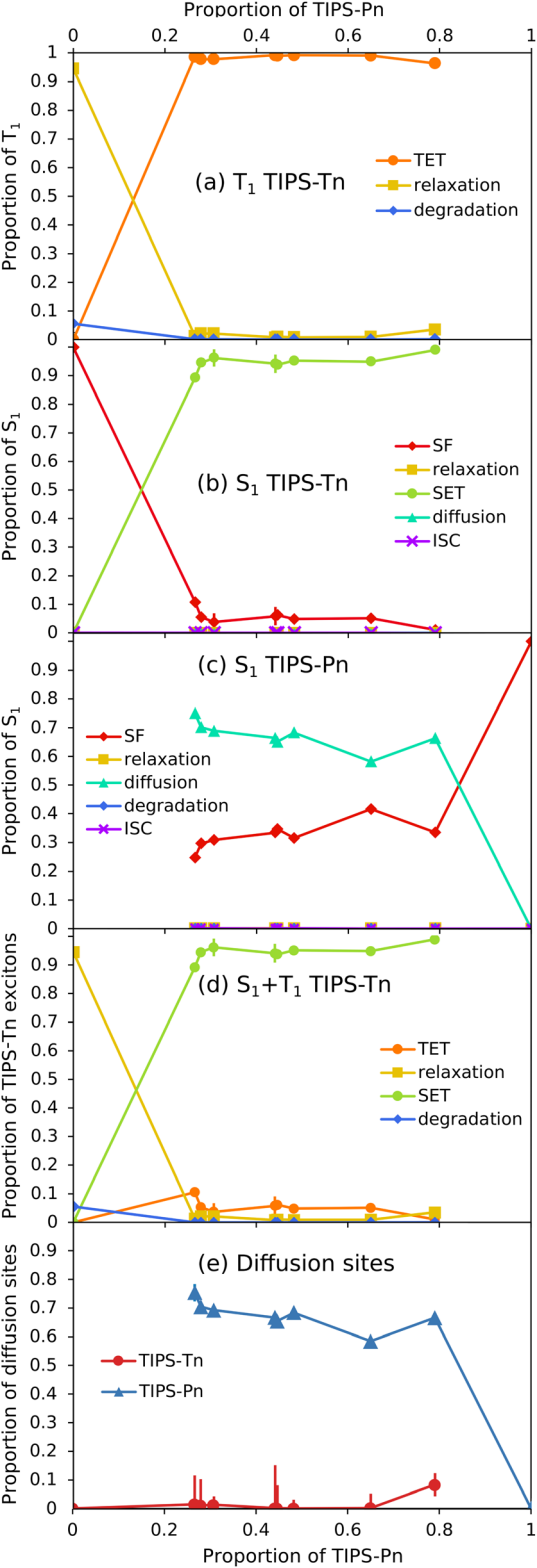
Relative importance of different excitonic processes from fitting the blend NP photodegradation. Shown are initial proportions of (a) TIPS-Tn T_1_, (b) TIPS-Tn S_1_, (c) TIPS-Pn S_1_, and (d) total TIPS-Tn (S_1_ + T_1_) excitons that undergo each process listed, *vs.* the proportion of TIPS-Pn in the NP (TIPS-Pn T_1_ is not shown as we assume 100% relaxation for this population). (e) Initial proportion of diffusion sites (*i.e.* before photodegradation) in the different blend NPs *vs.* the proportion of TIPS-Pn in the NP. Replicate experiments are included as individual data points, rather than averaged. Error bars are included in all plots as 1.645 times the standard error in the fitted value (*i.e.* a 90% confidence interval). Note that most error bars are smaller than the markers.

**Fig. 8:**
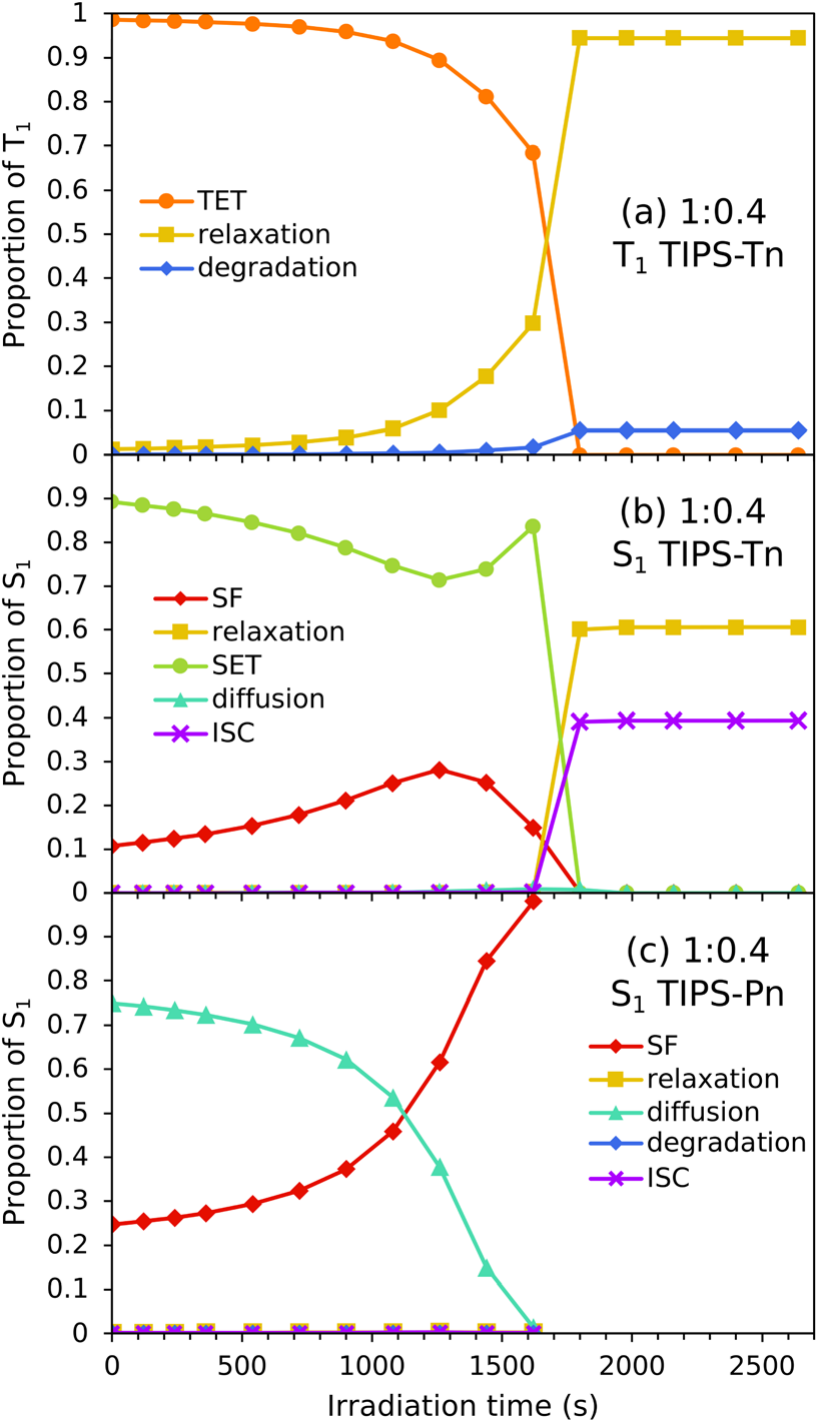
Change in relative importance of different processes for 1 : 0.4 TIPS-Pn : TIPS-Tn NPs over time as the NP degrades, for (a) TIPS-Tn T_1_, (b) TIPS-Tn S_1_, and (c) TIPS-Pn S_1_. Note that proportions of relaxation, degradation, and ISC are coincident (too small to resolve) in (c).

Now that modeling has established that TET from TIPS-Tn to TIPS-Pn does occur in this system (as the photodegradation cannot be fit well without it), we can use the fitted rate constants to quantify the extent of TET and other processes, and explain the photodegradation behavior (Fig. 7). For neat TIPS-Tn NPs, 94% of triplet excitons undergo relaxation to the ground state, and only 6% are quenched *via* photodegradation with oxygen (Fig. 7a). Despite this small proportion of degradation, the continuous excitation of NPs means that the neat TIPS-Tn NPs are completely degraded in a matter of minutes. In the blend NPs, the proportion of relaxation and degradation is much smaller, and most TIPS-Tn triplet excitons now undergo TET to TIPS-Pn. Even for the NPs with the smaller amount of TIPS-Pn (1 : 0.4, 27% TIPS-Pn) our modeling predicts 98% of TIPS-Tn triplet excitons undergo TET to TIPS-Pn. The proportions in Fig. 7a are determined using the initial (before degradation) concentrations of TIPS-Pn and TIPS-Tn in the NPs. As the molecules degrade, the concentrations of TIPS-Tn and TIPS-Pn change, and thus so do the rates and relative proportions of the bimolecular processes in the model. This behavior is demonstrated in Fig. 8, which shows how the proportion of each process changes over time for 1 : 0.4 blend NPs. For TIPS-Tn triplet excitons, TET to TIPS-Pn initially dominates, so there is little photodegradation of TIPS-Tn and the decrease in TIPS-Tn concentration is relatively slow. However, as the TIPS-Pn population decreases, TET to TIPS-Pn becomes slower and less efficient, and more relaxation and photodegradation occur. Once the TIPS-Pn is almost completely degraded, the amount of photodegradation and TET again reach the same proportions as in the neat NPs, and the TIPS-Tn population rapidly drops.

The modeling suggests that TET from TIPS-Tn to TIPS-Pn is very efficient in this system, which indicates that triplets resulting from TIPS-Tn SF can be effectively separated by the incorporation of a lower triplet energy chromophore, as is the intention of an energy-gradient design. However, modeling also shows that energy transfer does not only occur from the TIPS-Tn triplet state. The data cannot be fit well without also including SET from TIPS-Tn to TIPS-Pn. The proportions of each process that the TIPS-Tn singlet excitons undergo are shown as a function of TIPS-Pn content in Fig. 7b. For neat TIPS-Tn NPs, close to 100% of singlet excitons undergo SF, but when TIPS-Pn is introduced SET from TIPS-Tn to TIPS-Pn becomes predominant. For 1 : 0.4 TIPS-Tn : TIPS-Pn NPs (27% TIPS-Pn), 90% of TIPS-Tn singlet excitons initially (*i.e.* before degradation) undergo SET to TIPS-Pn, leaving only 10% to undergo SF. This grows to 99% SET for 1 : 4.2 blend NPs (80% TIPS-Pn). These results suggest that the yield of TIPS-Tn triplet excitons must be greatly reduced in these NPs. The change in the relative proportions of each process as the NPs degrade is shown for TIPS-Tn singlet excitons in 1 : 0.4 NPs in Fig. 8b. Initially, predominantly SET to TIPS-Pn occurs, but as the concentration of TIPS-Pn decreases the efficiency of SET decreases. Since TIPS-Tn molecules initially degrade much slower than the TIPS-Pn molecules in the NPs, the rate of TIPS-Tn SF decreases less than the rate of SET to TIPS-Pn, so SF becomes more prevalent over time. Eventually, the amount of SET to TIPS-Pn decreases enough that TIPS-Tn degradation becomes significant, and SF then decreases in efficiency. The balance of TIPS-Pn and TIPS-Tn degradation rates causes SET to slightly increase again around 1500 s. Once the TIPS-Pn concentration approaches zero, SET to TIPS-Pn becomes negligible, and SF causes the remainder of the TIPS-Tn SF sites to degrade (individual diffusion and SF populations are shown in Fig. S28†). Eventually, at late times, only TIPS-Tn diffusion sites remain, and therefore the dominant processes for TIPS-Tn singlet excitons become ISC and relaxation to the ground state.

The fate of excitons generated on TIPS-Pn is more straightforward. The dominant processes for singlet excitons on TIPS-Pn are shown as a function of TIPS-Pn content in Fig. 7c and as a function of irradiation time for 1 : 0.4 TIPS-Tn : TIPS-Pn NPs in Fig. 8c. The dominant processes for TIPS-Pn singlet excitons are either diffusion to other TIPS-Pn molecules or SF, and the relative ratio between them is governed by the relative amount of diffusion and SF sites in the NP. As the proportion of TIPS-Pn in the blend NPs increases, the number of SF sites increase, and diffusion sites decrease (Fig. 7e). Consequently, less diffusion occurs, and more SF (Fig. 7c). As singlet exciton diffusion in TIPS-Pn is slower and less competitive with photodegradation than SF, diffusion sites degrade faster than SF sites (see Fig. S29†). Hence for each blend NP sample the proportion of SF increases with irradiation time, and the proportion of diffusion decreases, as is shown for the 1 : 0.4 sample in Fig. 8c. The fate of excitons in the 1 : 4.2 TIPS-Tn : TIPS-Pn NPs is also shown as a function of irradiation time in the ESI, for which the behavior is largely the same, only with more efficient energy transfer from TIPS-Tn, and less TIPS-Pn diffusion, as would be expected (Fig. S31†).

The amount of TIPS-Pn singlet exciton diffusion is partly responsible for the trend in photodegradation observed in Fig. 3b: less TIPS-Pn in the NP means more TIPS-Pn diffusion sites, and faster photodegradation. However, the amount of SET from TIPS-Tn likely plays a larger role: more SET means more TIPS-Pn singlet excitons, and therefore faster TIPS-Pn photodegradation from the S_1_ state. This outcome is essentially equivalent to having a higher TIPS-Pn excitation rate. The trend in TIPS-Tn photodegradation in Fig. 3a is predominantly a result of changes in the TIPS-Pn concentration rather than any changes in the efficiency of SET or TET from TIPS-Tn to TIPS-Pn. At all mass ratios, both SET and TET are very efficient, and the increased level of these processes with increasing proportion of TIPS-Pn is minor (Fig. 7a and b). However, greater amounts of TIPS-Tn in the NP means more SET to TIPS-Pn, and more TIPS-Pn diffusion sites (hence less TIPS-Pn SF). More TIPS-Pn diffusion sites result in faster TIPS-Pn depletion, and therefore the rapid decay of TIPS-Tn occurs sooner. It would be interesting to monitor the photodegradation of even lower proportions of TIPS-Pn, to determine at what point the efficiency of TET from TIPS-Tn to TIPS-Pn begins to drop below the efficiencies observed here. The SET efficiency is slightly lower than that of TET (Fig. 7a and b), suggesting there may be a point of optimization where SET is inefficient, meaning TIPS-Tn SF is not inhibited, but TET to TIPS-Pn still occurs. This could explain why Wagner *et al.* were able to observe pentacene triplets at low pentacene doping concentrations in crystalline tetracene, after tetracene was excited.^26^ This investigation remains work for a future study.

Most parameters in the blend model used in this study could be fit sensitively, and many of the error bars in Fig. 7 are too small to be resolved. A notable exception to this rule is the proportion of diffusion sites in TIPS-Tn. Interestingly, the initial population of diffusion sites for TIPS-Tn is very small, even for NPs with high proportions of TIPS-Pn, for which we would expect the TIPS-Tn to be well diluted. It is possible that the TIPS-Tn and TIPS-Pn are not very intermixed, and that there are large TIPS-Tn domains rather than TIPS-Tn being evenly distributed. However, based on previously reported diffusion constants of amorphous pentacene and tetracene,^12,40,43^ the diffusion length in a pure domain (*i.e.* assuming singlet and triplet lifetimes measured in neat NPs) would be less than 2 nm (approximately twice the molecular diameters) for both singlet and triplet excitons (using 
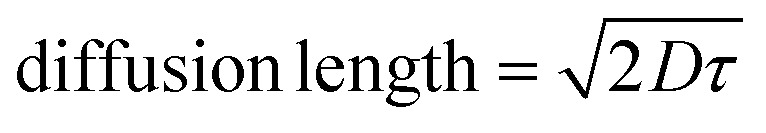
, where *D* is the diffusion constant and *τ* is the exciton lifetime). Given the NPs were around 60–90 nm in diameter, very few excitons would be able to be transferred from TIPS-Tn to TIPS-Pn in a largely phase separated structure. Given the large proportions (>90%) of SET and TET we observed, the molecules must have been well mixed such that very little exciton diffusion was necessary. Alternatively, the low initial population of TIPS-Tn diffusion sites may arise from diffusion sites having little impact on the TIPS-Tn photophysics. SET from TIPS-Tn to TIPS-Pn is consistently much faster than TIPS-Tn SF, so completely turning SF off for a portion of molecules by including a population of diffusion sites would only have a small impact on the fit. Hence the sensitivity of the initial TIPS-Tn diffusion site population is low, and the error bars are larger than for other parameters. The lack of TIPS-Tn diffusion sites could also be explained by singlet heterofission (S_1,Tn_ + S_0,Pn_ → T_1,Tn_ + T_1,Pn_), which we have neglected in this model, but has previously been reported in tetracene/pentacene blend films.^25^ It is possible that there is no initial TIPS-Tn diffusion population because even when surrounded by TIPS-Pn molecules, TIPS-Tn is still able to undergo SF with TIPS-Pn. Because TET is so efficient, singlet heterofission would be indistinguishable from SF on TIPS-Tn and subsequent TET using these data, so we do not attempt to differentiate between them.

Because there are negligible amounts of diffusion sites in TIPS-Tn, the rate constants of TIPS-Tn singlet exciton diffusion were also fit with very low sensitivity and have large errors (Tables S4 and S5†). Any diffusion sites that form over time as the NP degrades (Fig. S28†) are dominated by SET, which also reduces the sensitivity of the diffusion rate constant. We note that there are some discrepancies in the trends of the fitting parameters with TIPS-Pn content, and therefore in the proportions in Fig. 7. These variations come from random error in the photodegradation data, rather than the sensitivity of the fitted parameters. Specifically, we suspect this variability comes from variations in the excitation source power, since the relative TIPS-Pn content is able to be very precisely determined spectroscopically, and the oxygen content is expected to be in excess. This error can also be seen in the replicate experiments for 1 : 0.4 (27 and 28% TIPS-Pn) and 1 : 0.9 (44 and 45% TIPS-Pn) NPs, which were fit independently and are shown as individual data points in Fig. 7. While the error in the fitted rate constants and proportions for each sample is small, there is some variability in the values between the different samples. Despite this variability, the overall proportions of SET and TET are reproducibly greater than 90%.

The model used in this study is relatively simple, but fits the data sufficiently well, so no additional complexity could be added without reducing the fitting sensitivity. As discussed above, some singlet heterofission may occur between TIPS-Tn and TIPS-Pn, but because of the high efficiency of TET to TIPS-Pn, it would lead to identical outcomes as homofission and subsequent TET. Note that heterofission cannot be invoked to explain the data instead of TET, as it would leave 50% of triplets on TIPS-Tn, and therefore overestimate the photodegradation. The model also does not distinguish between triplet pairs and free triplet excitons, and assumes that both react with oxygen or can be transferred to TIPS-Pn with the same efficiency. This assumption may be somewhat imprecise, but the fitted parameters can still be considered as an average for the two populations. We also neglect triplet exciton diffusion within domains of the same material in this model. Triplet diffusion may very subtly change the populations of SF and diffusion sites (*e.g.* TIPS-Tn triplets generated on SF sites could migrate to diffusion sites, making this population degrade faster), and thus may change the relative amounts of SF and diffusion sites required for TIPS-Tn. However, neglecting triplet diffusion will not change the proportions of SET and TET between TIPS-Tn and TIPS-Pn, which is our main concern. Additionally, the TIPS-Tn singlet exciton diffusion rate constant is fit as very slow relative to the other processes, so we would also expect triplet exciton diffusion to be slow, and likely negligible compared to the rate of TET. The triplet exciton diffusion constant has previously been measured in amorphous TIPS-Pn as 1.33 × 10^−7^–1 × 10^−6^ cm^2^ s^−1^.^43^ The longest TET time constant that was fit in our study was 450 ps, so even if the diffusion constant in amorphous TIPS-Tn was an order of magnitude faster than TIPS-Pn (*i.e.* 10^−5^ cm^2^ s^−1^), the triplet would only be able to diffuse ∼1 nm in the time it takes for TET to occur, which is approximately the diameter of the molecule. Finally, the concentration of excitons generated by irradiation in the photodegradation experiments is lower than that of previous transient absorption experiments of TIPS-Tn and TIPS-Pn NPs, for which no exciton–exciton annihilation was observed. We thus rule out any exciton–exciton annihilation occurring in the data here. Charges on pentacene and tetracene chromophores have previously been shown to have distinct spectral features.^44–46^ These features have not been observed in prior studies of oxygenated solutions or NPs (ruling out charge transfer with oxygen),^12,34^ nor in pentacene/tetracene blend films,^25^ so we do not expect charge transfer to be playing a significant role in the system studied here. While it is tempting to include triplet pairs, heterofission, and triplet exciton diffusion to the model, the fits are already sufficient without them, so no extra unknown parameters could be included with any sensitivity. A different approach, such as Monte Carlo simulations of a molecular-scale model, may be able to account for these factors, but it is far beyond the scope of this work, and would offer little additional insight.

As discussed above, our results are consistent with an intermixed blend NP morphology, rather than a largely phase separated or core–shell structure, which was to be expected given the NP preparation procedure and similar chemical structure of the molecules (ESI Section S1.1†). We can also rule out a core–shell-like structure by considering the effect it would have on photodegradation. If TIPS-Tn were primarily located at the surface of the NPs (*i.e.* the shell), then the TIPS-Pn photodegradation should become slower in the blend NPs, rather than faster as is observed. If TIPS-Tn were primarily in the core of the NPs, then TIPS-Tn photodegradation may be suppressed by a protective shell of TIPS-Pn, but, this would not explain the phenomenon of TIPS-Pn in blend NPs degrading faster than in neat NPs. A TIPS-Tn core would also not explain the shape of the photodegradation curve with time, particularly the rapid decrease in the TIPS-Tn population once TIPS-Pn has degraded (Fig. 3). If the reason for TIPS-Tn photodegradation being suppressed were merely shielding from oxygen by TIPS-Pn, then the TIPS-Pn endoperoxide products should also be able to shield the TIPS-Tn core and the rapid decrease would not be observed. It could be argued that the conversion to endoperoxides breaks the NPs apart and exposes the TIPS-Tn, but we would then expect the degree of scattering in the solution to increase, which it does not (Fig. 2b).

The observation of significant SET to TIPS-Pn is consistent with the results of Zeiser *et al.* on crystalline tetracene/pentacene films.^25^ We can now conclude that the energy gradient is ineffective for a tetracene/pentacene chromophore system in two different morphologies, crystalline and amorphous. However, Zeiser *et al.* observed exclusively SET when tetracene was excited in the crystalline films, while our model suggests that a very small but non-negligible amount of TIPS-Tn SF and TET to TIPS-Pn was able to occur in the amorphous NPs. This finding indicates that there are some molecular arrangements where SF and TET are favored over SET. Different crystalline arrangements of these chromophores may therefore be more favorable, which could be achieved by alternative fabrication techniques,^47,48^ or through the addition of different side groups on tetracene and pentacene chromophores.^13,49^ We note that engineering specific molecular arrangements between pentacene and tetracene will likely not be an easily implemented design principle. Alternatively, a more efficient energy-gradient system could be engineered by selecting chromophores for which SET is energetically unfavorable (whilst maintaining favorable TET). The energy requirements for energy-efficient SF, *E*(S_1_) ≈ 2 × *E*(T_1_), leads to a trade-off between maximizing TET relative to SET, and minimizing energy losses in SF. If the S_1_ energy of one SF chromophore is higher to avoid SET, the T_1_ energy would also need to be higher to maintain *E*(S_1_) ≈ 2 × *E*(T_1_), but this may then prohibit TET. The most efficient energy-gradient system may be one in which SF is slightly endoergic for one chromophore, and slightly exoergic for the other, such that S_1_ and T_1_ energies are slightly lower and slightly higher, respectively, for the first chromophore with respect to the second. For example, rubrene (*E*(S_1_) = 2.23 eV, *E*(T_1_) = 1.14 eV)^50,51^ and 9,10-bis(phenylethynyl)anthracene (BPEA) (*E*(S_1_) = 2.40 eV, *E*(T_1_) = 1.11 eV)^50,52^ satisfy these requirements. Alternatively, SET could also be reduced in systems with less spectral overlap between donor emission and acceptor absorption (decreasing the rate of FRET).

## 3 Conclusions

We prepared amorphous suspensions of TIPS-Tn:TIPS-Pn blend NPs to study the effect of a triplet energy gradient in a disordered solid-state system of singlet-fission chromophores, in which many molecular arrangements are sampled. The photodegradation of these NP suspensions, which occurs over a time scale of hours, was able to reveal insight on ultrafast processes such as singlet fission and energy transfer. While these processes are overly fast to be directly observed in this study, their products (*i.e.* singlet or triplet excitons) significantly impact the rate of photodegradation, allowing us to quantify the level at which they occur. We found that TIPS-Tn degrades rapidly in neat NP suspensions, but when TIPS-Pn is added this degradation is suppressed by reducing the TIPS-Tn triplet population. Modeling showed that the reduction in the triplet population must be due to both TET and SET from TIPS-Tn to TIPS-Pn. TET was shown to be very efficient in this system, even in NPs with only ∼30% TIPS-Pn. This behavior is beneficial for energy-gradient driven SF: after SF on TIPS-Tn, triplets are transferred to TIPS-Pn, ideally helping them to separate and reducing the chances of recombination. However, SET is also very efficient, and acts to suppress SF on TIPS-Tn, so only a very small proportion of TIPS-Tn triplets are produced in the first place. SF is still able to occur on TIPS-Pn, but TET from TIPS-Pn to TIPS-Tn is absent, and there is no energy gradient to assist the separation of TIPS-Pn triplets. Ultimately, the energy gradient impedes SF in this system rather than assisting it. Our results indicate that chromophore pairs must be carefully arranged or selected to switch off SET for the energy gradient approach to be beneficial in enhancing SF. Engineering precise molecular arrangements that allow TET but suppress SET may not be easily achievable, so selecting different chromophores for which SET is energetically unfavorable will likely be a more fruitful approach.

## Data availability

Data and code are available at https://doi.org/10.6084/m9.figshare.27151443.

## Author contributions

A. N. S. conceptualized the project, carried out the experimental investigation, developed the methodology, performed the formal analysis and wrote the original draft. J. M. d. l. P. developed the methodology and collected preliminary data. D. M. H. conceptualized and supervised the project. T. W. K. conceptualized and supervised the project. All authors reviewed and edited the manuscript.

## Conflicts of interest

There are no conflicts to declare.

## Supplementary Material

SC-016-D4SC06702A-s001
