# Supplementary material for: Photodegradation reveals that singlet energy transfer impedes energy-gradient-driven singlet fission in polyacene blends
Source: Chem Sci. 2025 Jan 14;16(7):3246–58. doi: 10.1039/d4sc06702a (PMC11744680; doi:10.1039/d4sc06702a)
Supplement: SC-016-D4SC06702A-s001 [file SC-016-D4SC06702A-s001.pdf]

Supplementary Information (SI) for Chemical Science.
This journal is © The Royal Society of Chemistry 2025

Supporting Information: Photodegradation Reveals that Singlet Energy Transfer Impedes Energy-gradient-driven Singlet Fission in Polyacene Blends

Alexandra N. Stuart,[*,†,‡] Jessica M. de la Perrelle,[†] David M. Huang,[*,†] and Tak W. Kee[*,†]

†*Department of Chemistry, The University of Adelaide, Adelaide, South Australia 5005, Australia*
‡*Current address: School of Chemistry, The University of Sydney, Sydney, New South Wales 2006, Australia*

E-mail: alexandra.stuart@adelaide.edu.au; david.huang@adelaide.edu.au; tak.kee@adelaide.edu.au
Phone: +61 (0)9351-4424; +61 (0)8313-5580; +61 (0)8313-5314

Contents

S1 Experimental Details **S2**
S1.1 Nanoparticle Preparation S2
S1.2 Nanoparticle Characterization S3
S1.3 Photodegradation Experiment S3

S2 Photodegradation Data **S6**
S2.1 Spectrophotometer Data S6
S2.2 Colorimeter Data S6

S3 Deconvolution and Basis Spectra Fitting **S10**

S4 Excitation Rate Calculations **S19**

S5 Reproducibility **S22**

S6 Kinetic Modeling **S23**
S6.1 Neat TIPS-Tn NP Degradation S23
S6.1.1 Model 1 . . . . . S23
S6.1.2 Model 2 . . . . . S25
S6.2 Neat TIPS-Pn NP Degradation S27
S6.2.1 Model 3 . . . . . S27
S6.2.2 Model 4 . . . . . S29
S6.3 TIPS-Tn:TIPS-Pn Blend NP Degradation S31

S1 Experimental Details

S1.1 Nanoparticle Preparation

NPs of various mass ratios of 5,12-bis(triisopropylsilylethynyl)tetracene (TIPS-Tn) (Lumtec) and 6,13-bis(triisopropylsilylethynyl)pentacene (TIPS-Pn) (Ossila) were prepared using the reprecipitation technique. Materials dissolved in a good solvent were rapidly injected into a poor solvent, causing them to aggregate (Figure S1). All NPs were prepared in the dark to prevent degradation, using only 700 nm light for visibility, which was not absorbed by either molecule.

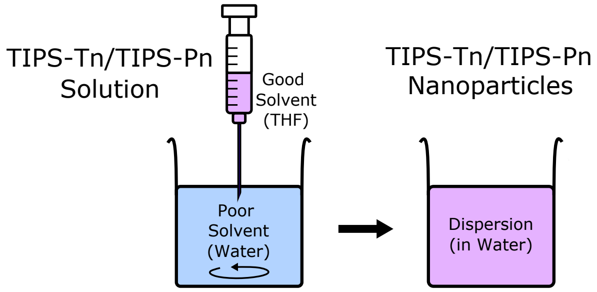

Figure S1: Nanoparticle preparation using the reprecipitation method.

The required proportion of TIPS-Tn and TIPS-Pn were dissolved in freshly distilled tetrahydrofuran (THF) (RCI Labscan) to a total concentration of 0.4 mg/mL ([TIPS-Tn]+[TIPS-Pn]=0.4 mg/mL). 3 mL aliquots of the THF solution were then injected into 15 mL of water (vigorously stirring) using a glass

syringe with a 0.55 mm needle diameter. This step was repeated with fresh water until the THF solution was used up. The fractions were then combined and THF and excess water removed under reduced pressure, until the desired concentration was reached ([TIPS-Tn] + [TIPS-Pn] $\approx$ 0.12 mg/mL). The blend NP suspensions were finally filtered through a 200 nm hydrophilic syringe filter (Sartorius Minisart NML). All water used in NP experiments was purified using an 18.2 MΩ cm Millipak Milli-Q IQ-7000 Water Purification System fitted with a 0.22 µm filter.

In the absence of templating agents such as PVA or other contaminants,[1–3] polyacene nanoparticles are formed with amorphous morphologies.[1,4,5] The absorption spectra (Figure S3) thus resemble those of TIPS-Pn and TIPS-Tn in solution, and lack the strong electronic coupling features observed in crystalline phases.[6–8] As discussed in the main text discussion, our results are consistent with these chromophores being well mixed, rather than a phase separated or core-shell like structure. The formation of large neat domains of chromophores would generally require some structural rearrangement to occur, which we would expect to be accompanied by an increase in crystallinity and interchromophore coupling, which is not evident in the absorption spectra of the blend NPs. Core-shell nanoparticles are more generally produced using a miniemulsion preparation technique,[9,10] which is more conducive to structural rearrangement. In this case, it is differences in chemical structure or solvent affinity that can cause one molecule to be more prevalent in the NP core than the other. Both because of the preparation procedure, and because TIPS-Tn and TIPS-Pn are very similar in chemical structure, we would not expect any rearrangement to a core-shell or phase-separated morphology to occur here.

S1.2 Nanoparticle Characterization

The size of the blend NPs was determined through dynamic light scattering (DLS) experiments (Malvern, Zetasizer Nano ZSP) with a 633 nm laser source and backscattering angle of 173°. The resulting NP diameters and polydispersity indices are given in Table S1. Previous work has shown that NP size affects the rates of singlet fission and other non-radiative decay processes, so NP diameters were kept between 60 and 90 nm, where differences due to size are minimal.[5] NPs were also only used if the polydispersity index was less than 0.3 (indicating the size distribution is relatively narrow).

Steady-state UV–visible absorption spectra were obtained with a Cary Varian 1E UV–visible spectrophotometer using a 1 cm path length quartz cuvette (Starna Cells 21-Q-10). Concentrations of TIPS-Tn and TIPS-Pn were determined from spectrophotometer data using a molar absorptivity of $\epsilon_{\mathrm{TIPS-Pn}}$(646 nm) = 16944 Lmol^{-1}cm^{-1} for TIPS-Pn and $\epsilon_{\mathrm{TIPS-Tn}}$(535 nm) = 23965 Lmol^{-1}cm^{-1} for TIPS-Tn (determined from neat NPs with known concentrations). The resulting molar and mass ratios determined from these concentrations are given in Table S1.

S1.3 Photodegradation Experiment

Before each photodegradation experiment, O_2 was bubbled through samples in a 1 cm quartz cuvette in the dark for at least two hours. The cuvettes were then sealed and the full absorption spectrum recorded on a Cary Varian IE UV-visible spectrophotometer, before being transferred to the photodegradation setup in Figure S2.

In the photodegradation experiments, a PASCO PS-3215 wireless colorimeter was used to record the absorbance of the samples over irradiation time. The colorimeter was used rather than a spectrophotometer in order to keep the position of the sample relative to the light source consistent, and to minimize light exposure between irradiation times.

The photodegradation setup consisted of a Xe light source with a 73 mWcm^{-2} spot with 1.3 cm radius (spectrum shown in Section S4), a sealed light-proof box kept at a fixed position relative to the light source with a small aperture to irradiate the sample, and a shutter to block the light when the sample was not being irradiated (i.e. when collecting colorimeter spectra). A magnetic stirrer was placed inside the box, along with a thermometer to record the temperature during the experiment. A small slot in the

Table S1: Fraction of TIPS-Pn for each TIPS-Tn:TIPS-Pn blend NP by mass and molar concentrations determined from steady-state absorption, as well as the nanoparticle diameter and polydispersity index as determined from DLS. The standard error in NP diameter is the two times the standard error in the mean calculated from triplets measurements of each sample.

mass ratio TIPS-Tn:TIPS-Pn	mole ratio TIPS-Tn:TIPS-Pn	mass fraction of TIPS-Pn	molar fraction of TIPS-Pn	NP diameter (nm)	polydispersity index
1:0.0	1:0	0.00	0.00	69.0 ± 0.4	0.107
1:0.4	1:0.4	0.29	0.27	69 ± 1	0.117
1:0.5	1:0.5	0.33	0.31	62 ± 2	0.258
1:0.9	1:0.8	0.47	0.45	58 ± 1	0.277
1:1.1	1:1	0.51	0.49	66 ± 3	0.234
1:2.1	1:1.9	0.68	0.66	67.1 ± 0.7	0.176
1:4.2	1:4.0	0.81	0.80	89.9 ± 0.9	0.061
1:5.7	1:5.3	0.85	0.84	62.5 ± 0.6	0.121
1:10.0	1:9.2	0.91	0.90	78.3 ± 0.8	0.107
0:1.0	0:1.0	1.00	1.00	64.2 ± 0.7	0.085

lid of the box accommodated a lever which was able to be moved up and down from outside the sealed box, to raise the cuvette from the colorimeter into the light path, and vice versa.

The photodegradation procedure was as follows: the PASCO colorimeter was calibrated with toluene or water, then placed in the box at a fixed distance from the light source in each experiment. The cuvette was attached to a lever and then sealed inside the box with the shutter closed. The lever was used to lower the cuvette into the colorimeter to record the spectrum at zero irradiation time, and then raise the cuvette out of the colorimeter into a fixed position in the light path. The shutter was then opened and sample irradiated for the appropriate time interval, then closed and the nanoparticle spectrum recorded again, repeating until approximately 5–10% of the original absorption remained. After the final colorimeter spectrum, the full absorbance spectrum of the degraded sample was also recorded on the spectrophotometer to assist with the data fitting and colorimeter correction. To determine the reproducibility of the experiment, seven and nine samples were prepared and degraded for neat TIPS-Pn and neat TIPS-Tn NPs, respectively (reproducibility reported previously[11]). Additionally, two replicates (with individually prepared samples) were degraded for 1:0.4 TIPS-Tn:TIPS-Pn NPs, and three replicates for 1:0.9 NPs (reproducibility in Section S5).

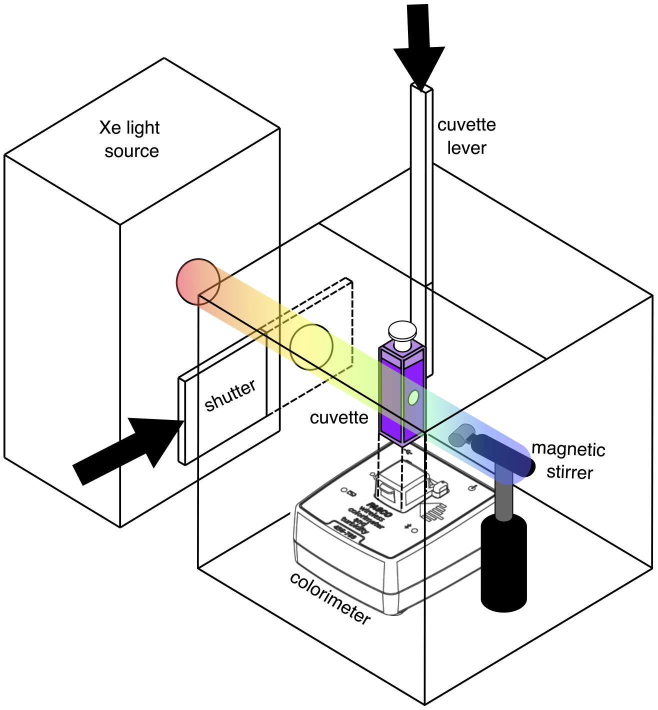

Figure S2: Schematic of the photodegradation experiment setup.

S2 Photodegradation Data

S2.1 Spectrophotometer Data

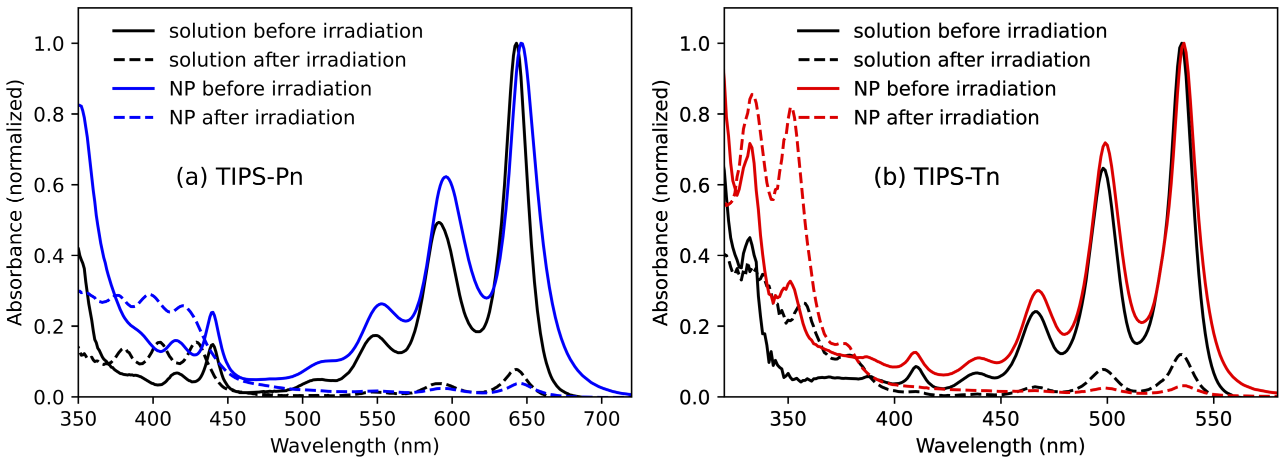

Figure S3: Steady-state absorption of (a) TIPS-Pn in toluene solution and neat TIPS-Pn NPs suspended in water and (b) TIPS-Tn in toluene solution and neat TIPS-Tn NPs suspended in water. Spectra are shown before irradiation (sold lines) and after 130, 73, 2.2, and 63 min of irradiation for TIPS-Pn NPs, TIPS-Pn solution, TIPS-Tn NPs, and TIPS-Tn solution, respectively (dashed lines).

S2.2 Colorimeter Data

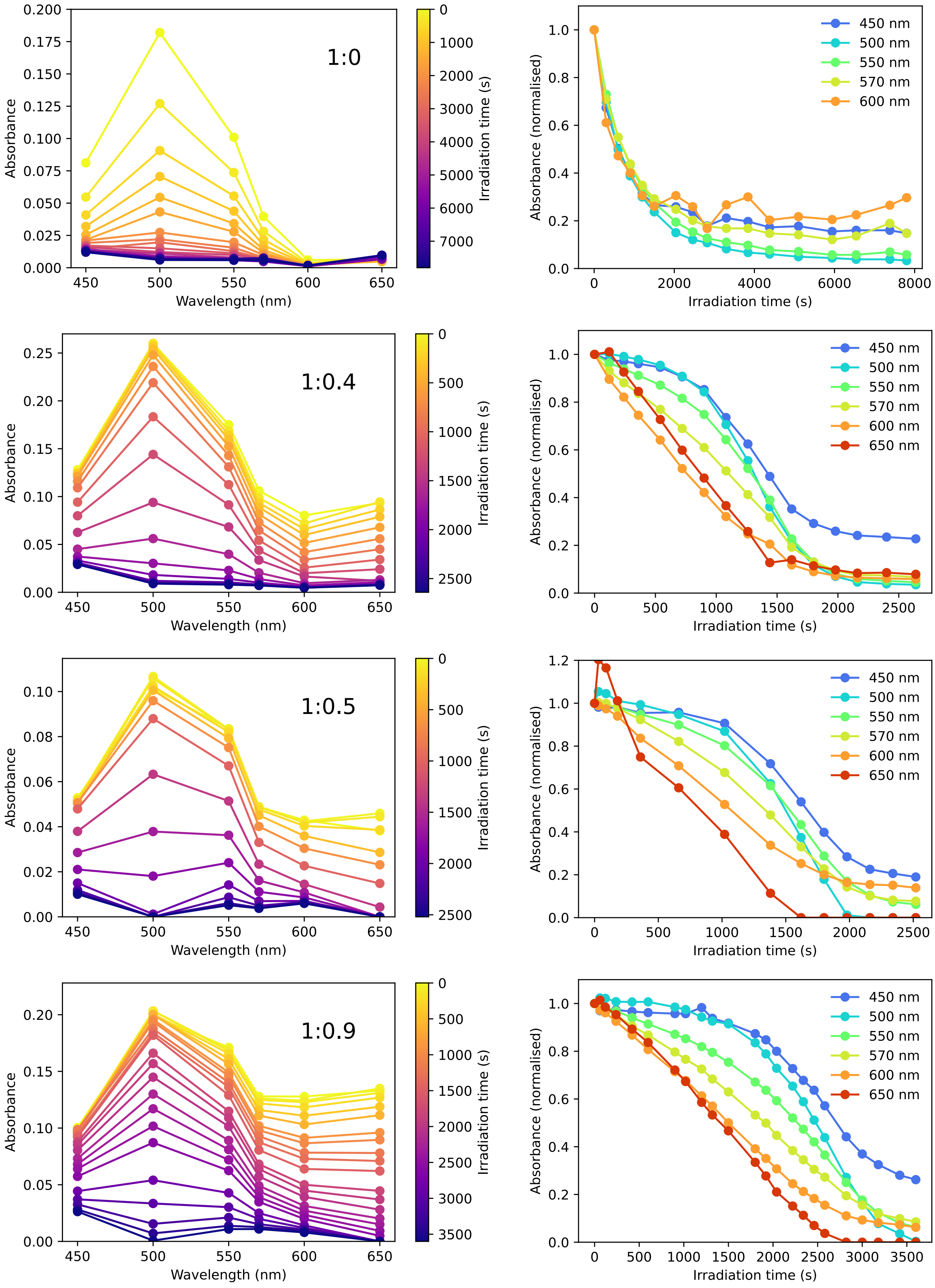

Figure S4: Photodegradation of blend NPs, with the TIPS-Tn:TIPS-Pn mass ratio indicated. Plots on the left show the absorbance as a function of wavelength, and the plots of the right show absorbance as a function of time. Absorbances plot as a function of time are normalized to the absorption at time zero. Wavelengths at which the absorption is zero (>570 nm for TIPS-Tn and < 500 nm for TIPS-Pn) are omitted.

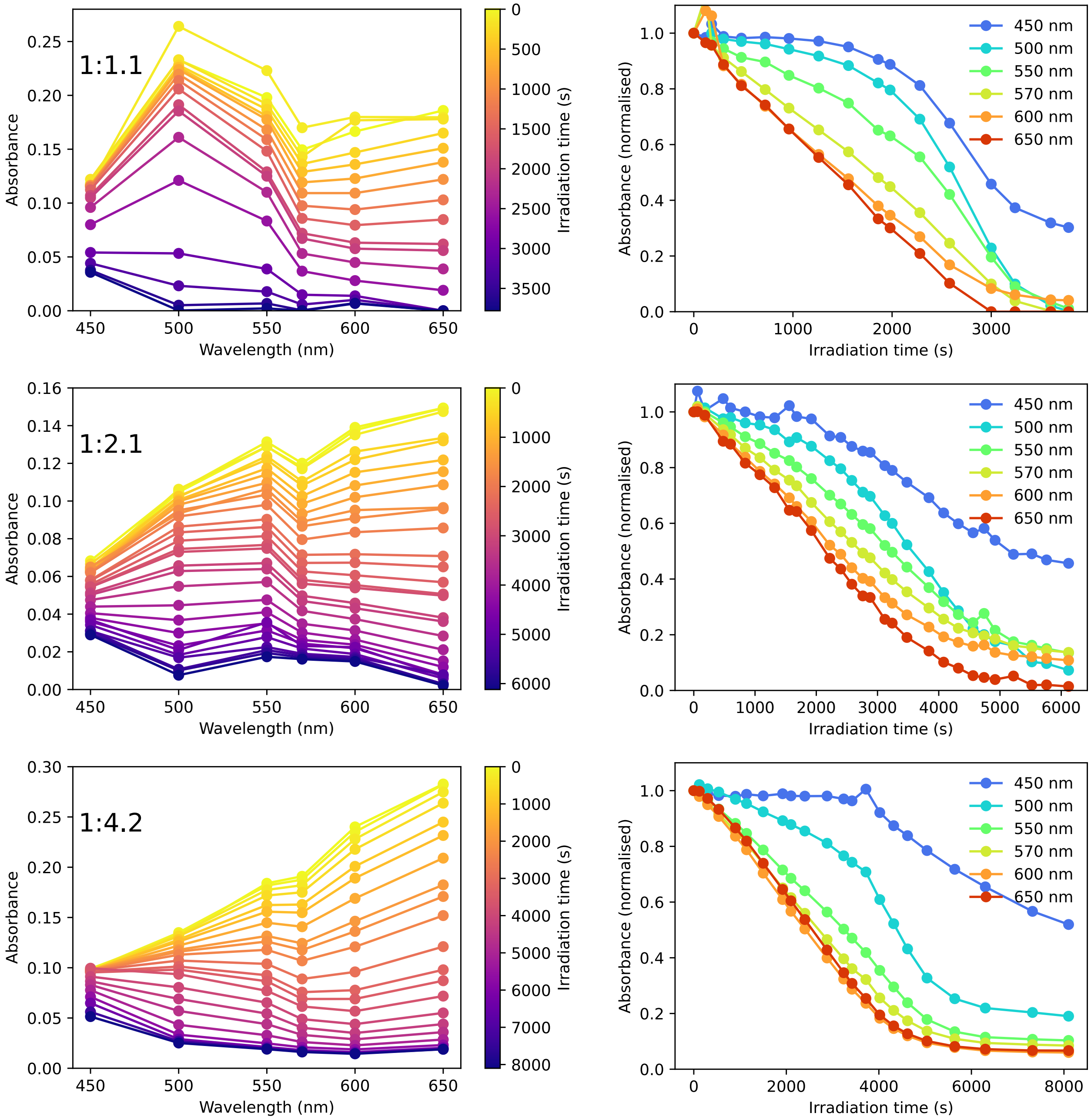

Figure S4: (Continued) Photodegradation of blend, with the TIPS-Tn:TIPS-Pn mass ratio indicated is indicated. Plots on the left show the absorbance as a function of wavelength, and the plots of the right show absorbance as a function of time. Absorbances plot as a function of time are normalized to the absorption at time zero. Wavelengths at which the absorption is zero (>570 nm for TIPS-Tn and < 500 nm for TIPS-Pn) are omitted.

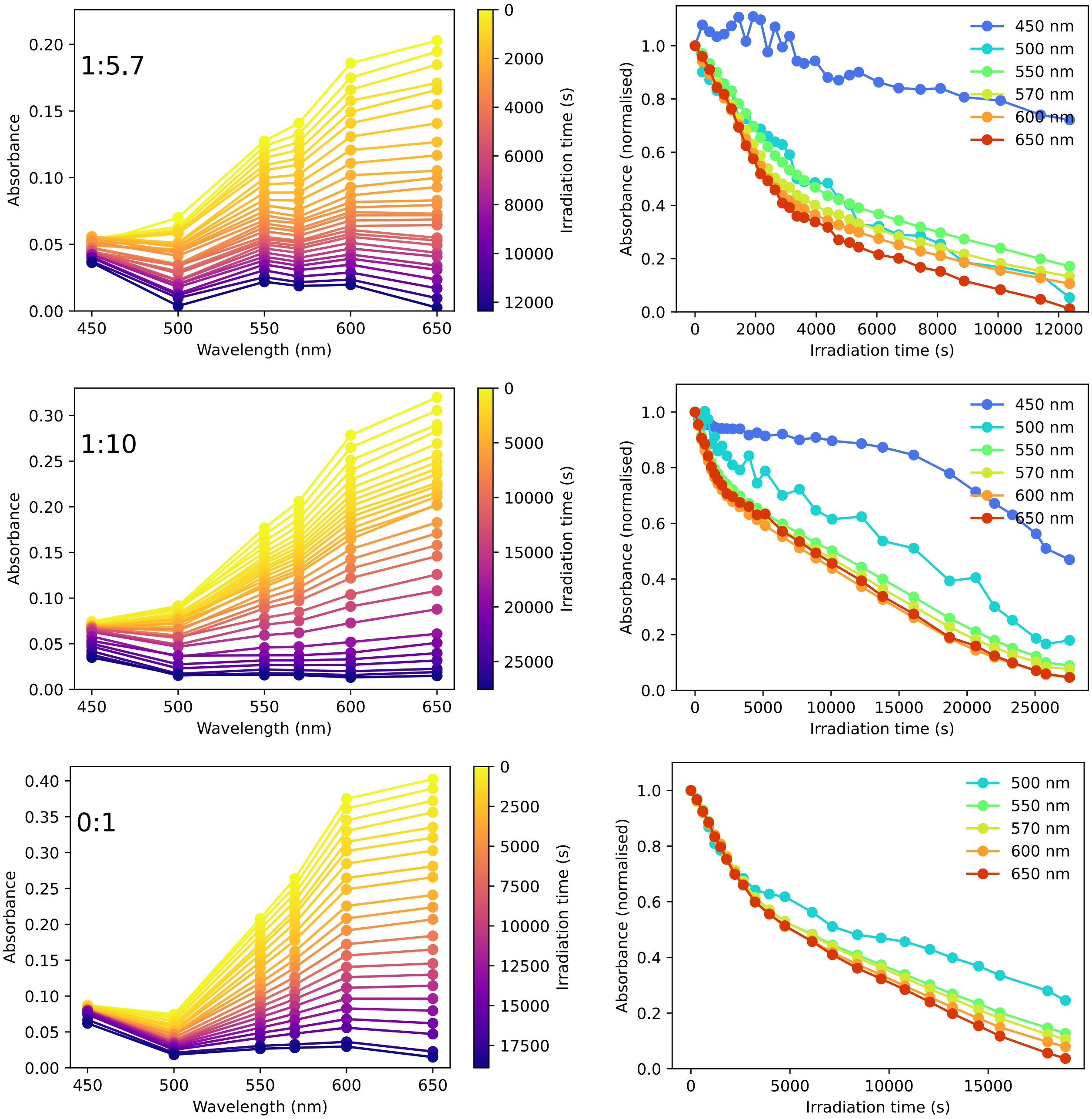

Figure S4: (Continued) Photodegradation of blend NPs, with the TIPS-Tn:TIPS-Pn mass ratio indicated. Plots on the left show the absorbance as a function of wavelength, and the plots of the right show absorbance as a function of time. Absorbances plot as a function of time are normalized to the absorption at time zero. Wavelengths at which the absorption is zero (>570 nm for TIPS-Tn and < 500 nm for TIPS-Pn) are omitted.

S3 Deconvolution and Basis Spectra Fitting

TIPS-Tn, TIPS-Pn, and their respective degradation products all have overlapping absorptions in the visible spectrum, so the change in concentration of each with irradiation time cannot be tracked using a single wavelength. To determine the photodegradation reaction kinetics, the spectra must be deconvoluted into their respective components, as described below.

To determine the amount of TIPS-Pn and TIPS-Tn initially in a blend NP, the neat TIPS-Pn NP spectrum and neat TIPS-Tn NP spectrum were recorded on the spectrophotometer, as in Figure S5a. The concentrations of these neat samples were used to determine the extinction coefficients of TIPS-Tn and TIPS-Pn at each wavelength λ, $\epsilon_{\mathrm{Tn}}(\lambda)$ and $\epsilon_{\mathrm{Pn}}(\lambda)$. Each TIPS-Tn:TIPS-Pn blend NP sample could then be fit with a linear combination of the two basis spectra to determine the concentration of TIPS-Pn and TIPS-Tn present using

$$A_{\mathrm{NP}}(\lambda) = \epsilon_{\mathrm{Pn}}(\lambda) c_{\mathrm{Pn}} l + \epsilon_{\mathrm{Tn}}(\lambda) c_{\mathrm{Tn}} l, \tag{S1}$$

where $A_{\mathrm{NP}}(\lambda)$ is the absorbance of the NP at wavelength λ (as recorded by the spectrophotometer), c_{Pn} and c_{Tn} is the concentration of TIPS-Pn or TIPS-Tn, respectively, and l is the pathlength of the cuvette (in all cases here this is 1 cm). Figure S6a shows that a linear combination of the neat NP spectra was able to reproduce the blend NP spectra well, indicating that no additional features arise (e.g from electronic coupling between TIPS-Pn and TIPS-Tn) and the two components are able to be treated as spectroscopically independent.

The neat NP samples were also used to determine the colorimeter basis spectra, $\epsilon_{\mathrm{Pn,\,colorimeter}}(\lambda)$ and $\epsilon_{\mathrm{Tn,\,colorimeter}}(\lambda)$ as shown in Figure S5b. Note that the colorimeter records just 6 wavelengths, each of which has a 40 nm FWHM, so the shape and magnitude of $\epsilon_{\mathrm{Pn,\,colorimeter}}(\lambda)$ and $\epsilon_{\mathrm{Tn,\,colorimeter}}(\lambda)$ are different to $\epsilon_{\mathrm{Pn}}(\lambda)$ and $\epsilon_{\mathrm{Tn}}(\lambda)$. The colorimeter basis spectra should be considered as scaling factors between concentration and colorimeter response, rather than the actual molar absorptivities of the sample.

Figure S6 shows an example of these basis spectra fit to 1:0.9 mass ratio TIPS-Tn:TIPS-Pn NP sample before and after 86 minutes of irradiation (the final time point of the photodegradation experiment for this sample). After irradiation there is an additional component in the NP spectra due to degradation products and the scattering of the NPs, as shown in Figure S6b. For these spectra a best fit of the individual TIPS-Pn and TIPS-Tn components is performed ensuring the residuals resemble a scattering

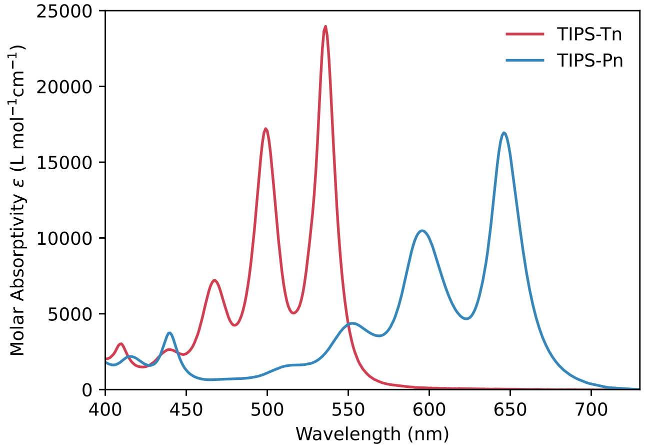

(a) Spectrophotometer

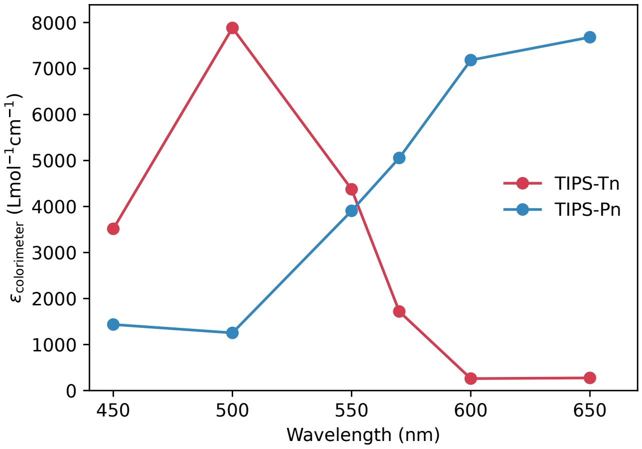

(b) Colorimeter

Figure S5: TIPS-Pn and TIPS-Tn basis spectra, as determined from the absorbance of neat TIPS-Pn and neat TIPS-Tn NPs recorded by the (a) Cary spectrophotometer and (b) the PASCO colorimeter. The colorimeter has a 40 nm FWHM, so the shape and magnitude of the colorimeter spectra is not the same as the spectrophotometer.

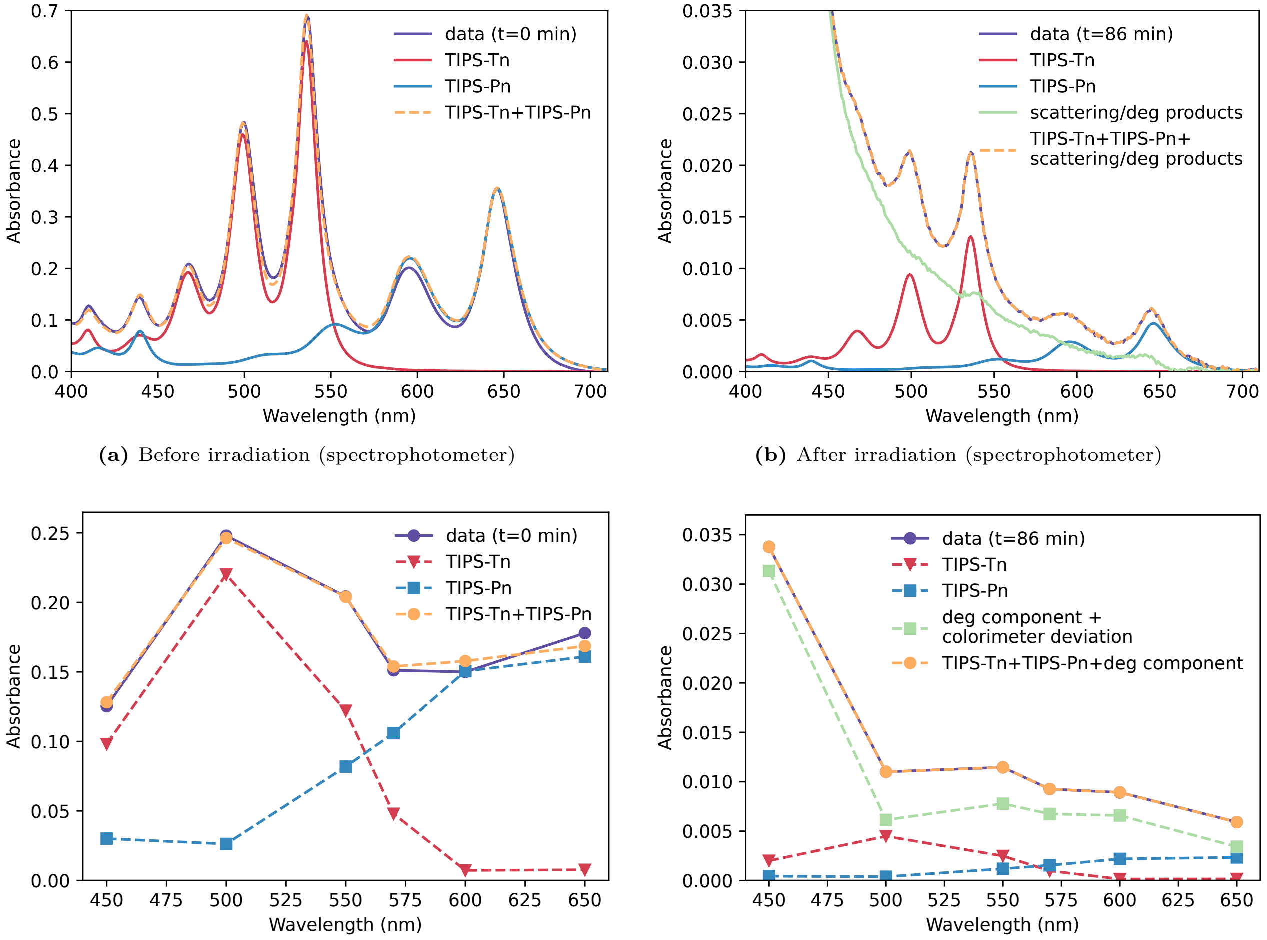

(a) Before irradiation (spectrophotometer)

(b) After irradiation (spectrophotometer)

(c) Before irradiation (colorimeter)

(d) After irradiation (colorimeter)

Figure S6: (a,b) Spectrophotometer and (c,d) colorimeter absorption spectra of a 1:0.9 (mass ratio) sample of TIPS-Tn:TIPS-Pn NPs (a,c) before and (b,d) after 86 minutes of irradiation, and the constituent spectra (TIPS-Pn, TIPS-Tn, and degradation product). The orange dashed lines are the linear combination of TIPS-Pn and TIPS-Tn basis spectra fit to the experimental blend data. The after irradiation spectra contains components due to light scattering and degradation products, and in the case of the colorimeter, also the error or deviation in the response at low absorbances. This particular sample is the 1:0.9 replicate with a TIPS-Tn concentration of 25 mM (Figure S14).

function (light green curve in Figure S6b). Degradation products would also be present in the residuals, but their absorbance is predominantly in the UV (<450 nm), so has little impact on these fits. Scattering is also present in the before irradiation spectrum, but is less significant relative to the NP absorbance and is accounted for by the neat NP basis spectra.

The colorimeter spectra can also be fit as shown in Figures S6c and d. It becomes difficult to fit the TIPS-Pn and TIPS-Tn components to the colorimeter spectrum as the absorbance becomes low (i.e. Figure S6d). As with the spectrophotometer data there are additional components due to degradation products and scattering, but the colorimeter additionally has a lower resolution and a non-linear response at low concentrations, which leads to further discrepancies or deviations in shape. To account for these extra factors and aid in resolving the colorimeter spectra, the amount of TIPS-Tn and TIPS-Pn present after irradiation in the colorimeter spectrum is fixed to the amount determined from the spectrophotometer data, p; i.e. for component m with spectrophotometer absorbance $A_{m,\text{spectrophotometer}}(\lambda, t)$ and

equivalent colorimeter absorbance $A_{m,\,\text{colorimeter}}(\lambda, t)$ at wavelength λ and irradiation time t,

$$p = \frac{A_{m\,\text{spectrophotometer}}(\lambda, t)}{A_{m,\,\text{spectrophotometer}}(\lambda, 0\text{ min})}. \tag{S2}$$

Hence, for the final data point of the experiment, which in the case of the 1:0.9 sample in Figure S6 is at $t = 86$ min,

$$p = \frac{A_{m\,\text{spectrophotometer}}(\lambda, 86\text{ min})}{A_{m,\,\text{spectrophotometer}}(\lambda, 0\text{ min})} = \frac{A_{m\,\text{colorimeter}}(\lambda, 86\text{ min})}{A_{m,\,\text{colorimeter}}(\lambda, 0\text{ min})},$$
$$\therefore A_{m,\,\text{colorimeter}}(\lambda, 86\text{ min}) = p \times A_{m,\,\text{colorimeter}}(\lambda, 0\text{ min}). \tag{S3}$$

For the 1:0.9 sample here, the spectrophotometer spectra show the proportion remaining (p) at 86 min is 0.015 for TIPS-Pn and 0.02 for TIPS-Tn. Hence

$$A_{\text{Pn},\,\text{colorimeter}}(\lambda, 86\text{ min}) = 0.015 \times A_{\text{Pn},\,\text{colorimeter}}(\lambda, 0\text{ min}),$$
$$A_{\text{Tn},\,\text{colorimeter}}(\lambda, 86\text{ min}) = 0.02 \times A_{\text{Tn},\,\text{colorimeter}}(\lambda, 0\text{ min}),$$

which is the magnitude of the spectra shown in Figure S6d. The degradation component (which is a combination of the degradation product absorption, scattering, and colorimeter error) is then determined to be

$$\begin{aligned} A_{\text{deg,colorimeter}}(\lambda, 86\text{ min}) = A_{\text{colorimeter}}(\lambda, 86\text{ min}) - A_{\text{Pn},\,\text{colorimeter}}(\lambda, 86\text{ min}) \\ - A_{\text{Tn},\,\text{colorimeter}}(\lambda, 86\text{ min}), \end{aligned} \tag{S4}$$

where $A_{\text{colorimeter}}(\lambda, 86\text{ min})$ is the observed colorimeter data at 86 min (purple circles in Figure S6d). The concentration of the degradation component is not important for the analysis. The amount of degradation product present should be equal to the amount of TIPS-Tn and TIPS-Pn that has degraded, so we set

$$c_{\text{deg}}(86\text{ min}) = c_{\text{Pn}}(0\text{ min}) + c_{\text{Tn}}(0\text{ min}) - c_{\text{Pn}}(86\text{ min}) - c_{\text{Tn}}(86\text{ min})$$

and use this concentration to determine a basis spectrum for this component,

$$\epsilon_{\text{deg},\,\text{colorimeter}}(\lambda) = \frac{A_{\text{deg},\,\text{colorimeter}}(\lambda, 86\text{ min})}{c_{\text{deg}}(86\text{ min}) \times l}, \tag{S5}$$

where l is the path length (1 cm). With these three components, TIPS-Tn, TIPS-Pn, and the degradation product, we can then fit the colorimeter spectra at each irradiation time point t using

$$\begin{aligned} A_{\text{colorimeter}}(\lambda, t) = &\epsilon_{\text{Pn},\,\text{colorimeter}}(\lambda)c_{\text{Pn}}(t)l + \epsilon_{\text{Tn},\,\text{colorimeter}}(\lambda)c_{\text{Tn}}(t)l \\ &+ \epsilon_{\text{deg},\,\text{colorimeter}}(\lambda)c_{\text{deg}}(t)l, \end{aligned} \tag{S6}$$

and determine the concentration of TIPS-Pn and TIPS-Tn, $c_{\text{Pn}}(t)$ and $c_{\text{Tn}}(t)$, over time. The fits to the 1:0.9 NP sample are shown in Figure S7, and the resulting concentration kinetics in Figure S8.

Figure S7 shows the three components, TIPS-Pn, TIPS-Tn, and degradation products, are able to reproduce the colorimeter spectra well at all stages of the degradation. The degradation product component includes both TIPS-Pn and TIPS-Tn degradation products, which do not necessarily form at the same rate, so the ratio of degradation products at 86 min (the time used to determine the shape of the spectrum) is not necessarily representative of the total degradation product shape at all times. For example, TIPS-Pn initially degrades faster, so the degradation product at early times should be predominantly TIPS-Pn derived, whereas at 86 min it is roughly equal between TIPS-Pn and TIPS-Tn. This may be

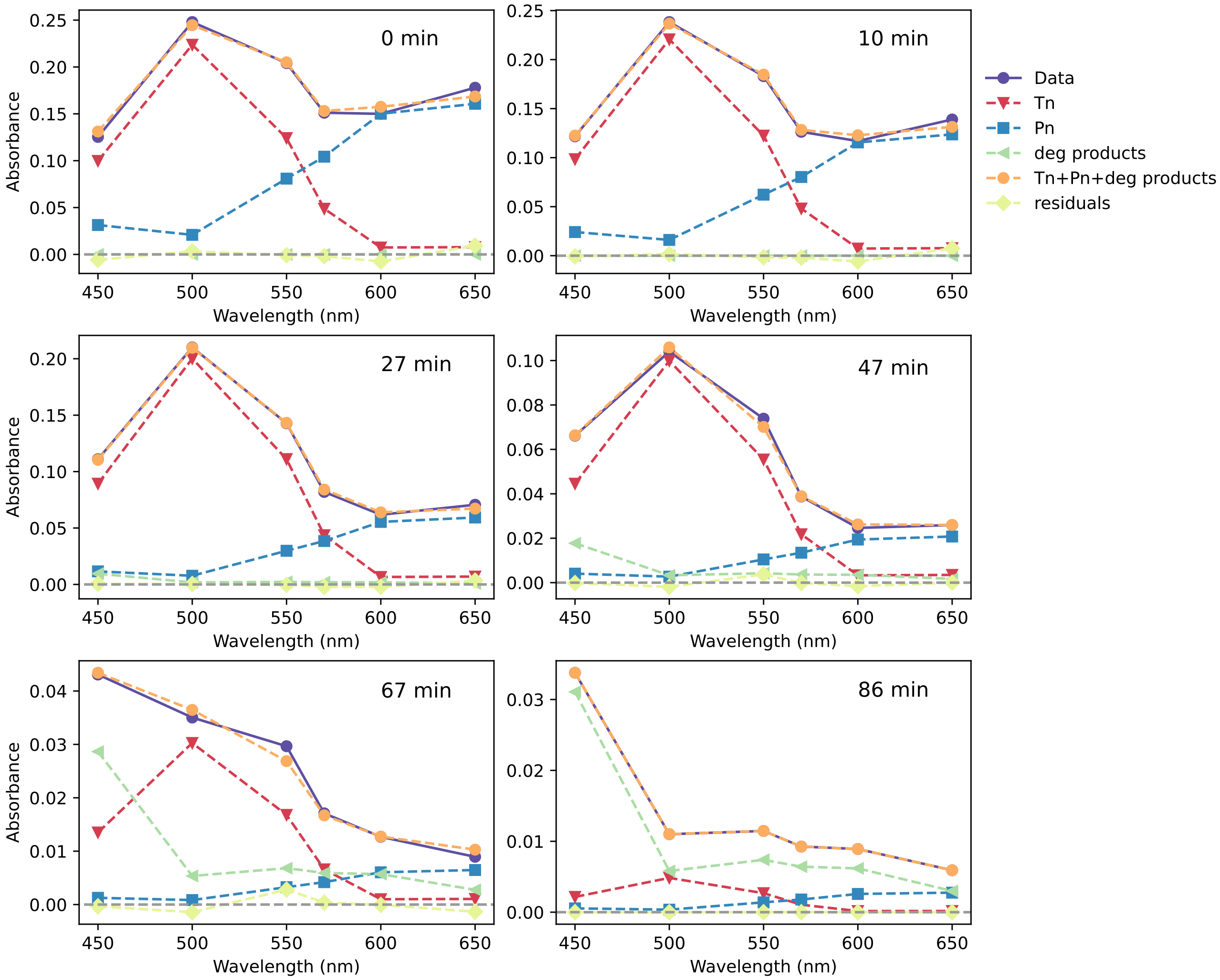

Figure S7: Fit of contributions of TIPS-Tn, TIPS-Pn, and degradation products to the colorimeter spectrum of a 1:0.9 TIPS-Tn:TIPS-Pn NP sample (the 25 mM replicate) for various irradiation times. Residuals are also shown. The TIPS-Tn and TIPS-Pn spectra are determined from neat samples, and the degradation spectrum is determined from the 86 min spectrum minus the remaining TIPS-Pn and TIPS-Tn components.

the origin of some of the discrepancies of the fits, but since both products endoperoxide absorptions are predominantly below 450 nm, and the overall fit quality is high, we conclude it is reasonable to treat the degradation products together.

The concentration kinetics in Figure S8 show the decay of TIPS-Pn and TIPS-Tn with irradiation time, and resultant increase in the amount of degradation product, which is artificially set at 86 min to equal the total amount of TIPS-Tn and TIPS-Pn that has degraded. The increase in the degradation product concentration over irradiation time roughly correlates with the decay of TIPS-Tn and TIPS-Pn. There are a few inconsistencies, which are explained by the fact that this component is not just due to degradation product absorption, but also has significant contributions due to scattering, and the error in the colorimeter response.

This method was used to fit the data for each blend NP sample. Where replicates exist (e.g. the 1:0.4 and 1:0.9 mass ratios), each replicate was fit individually. The fits are shown for select times in Figure S9 , and the resultant concentrations in Figure S10. Given the separated TIPS-Pn and TIPS-Tn concentration kinetics, the degradation can then be modeled to determine the rates of the photophysical processes in the system, as detailed in Sections S4 and S6.

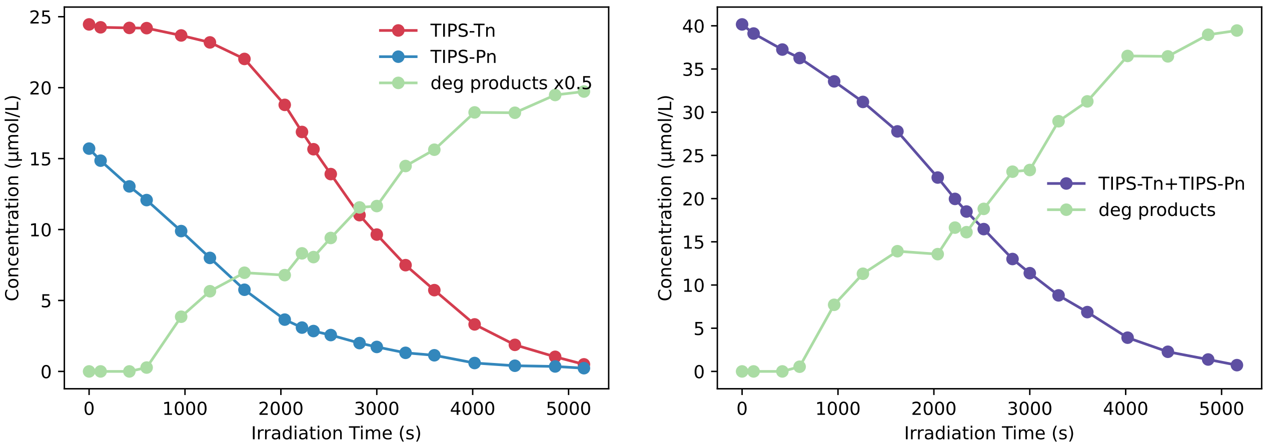

Figure S8: Fitted concentration of TIPS-Tn, TIPS-Pn, and degradation products over irradiation time to a sample of 1:0.9 TIPS-Tn:TIPS-Pn NPs (25 mM TIPS-Tn).

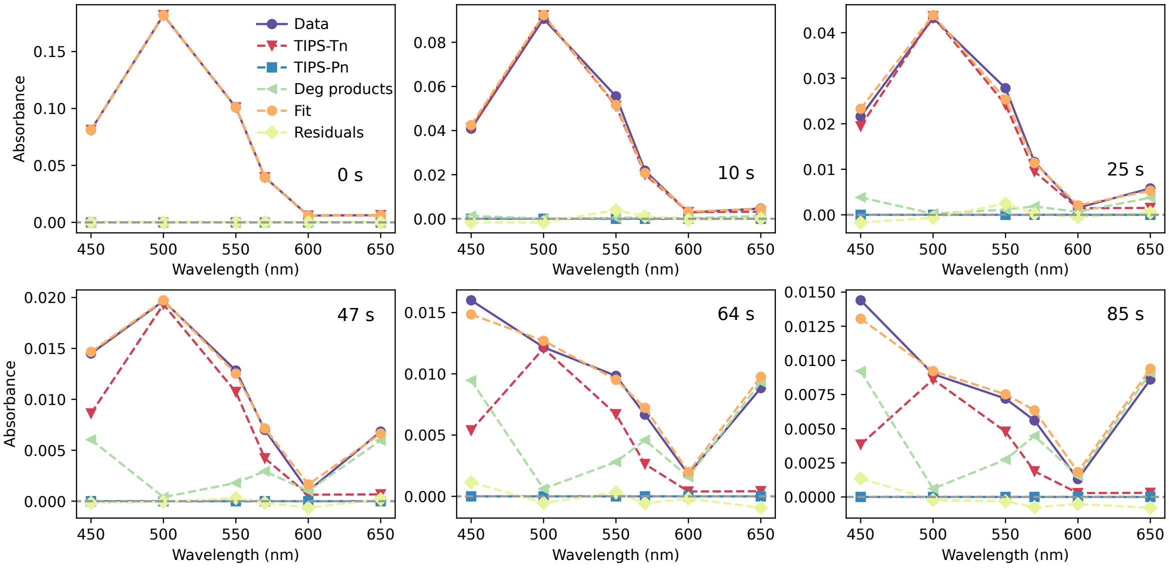

(a) 1:0 TIPS-Tn:TIPS-Pn

Figure S9: Fit of TIPS-Tn, TIPS-Pn, and degradation products to NP spectra at various TIPS-Tn:TIPS-Pn mass ratios for select irradiation times. One example fit is given for each NP mass ratio.

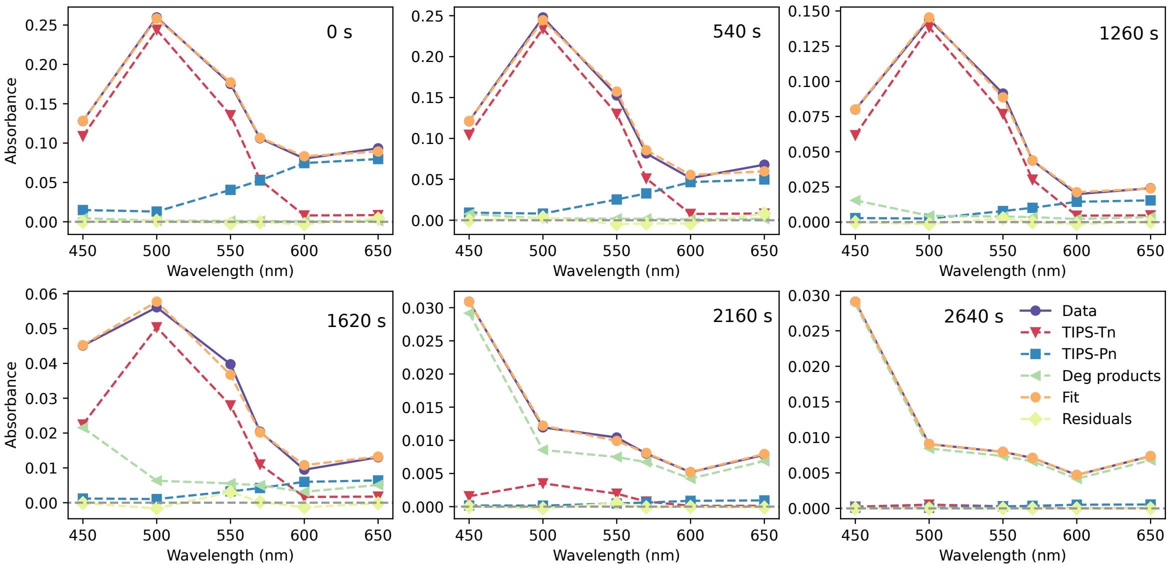

(b) 1:0.4 TIPS-Tn:TIPS-Pn

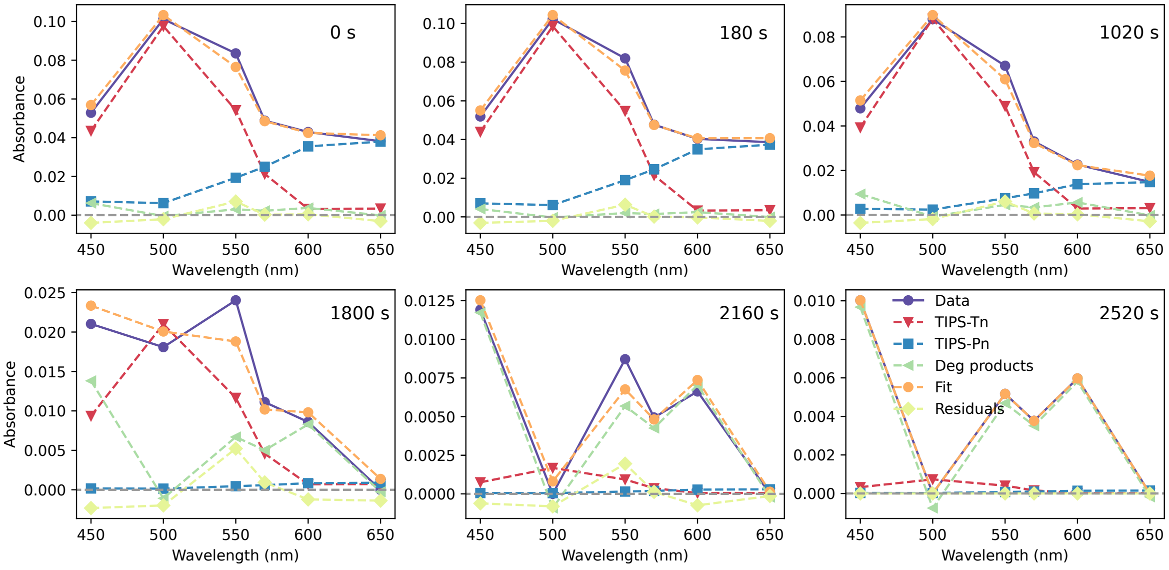

(c) 1:0.5 TIPS-Tn:TIPS-Pn

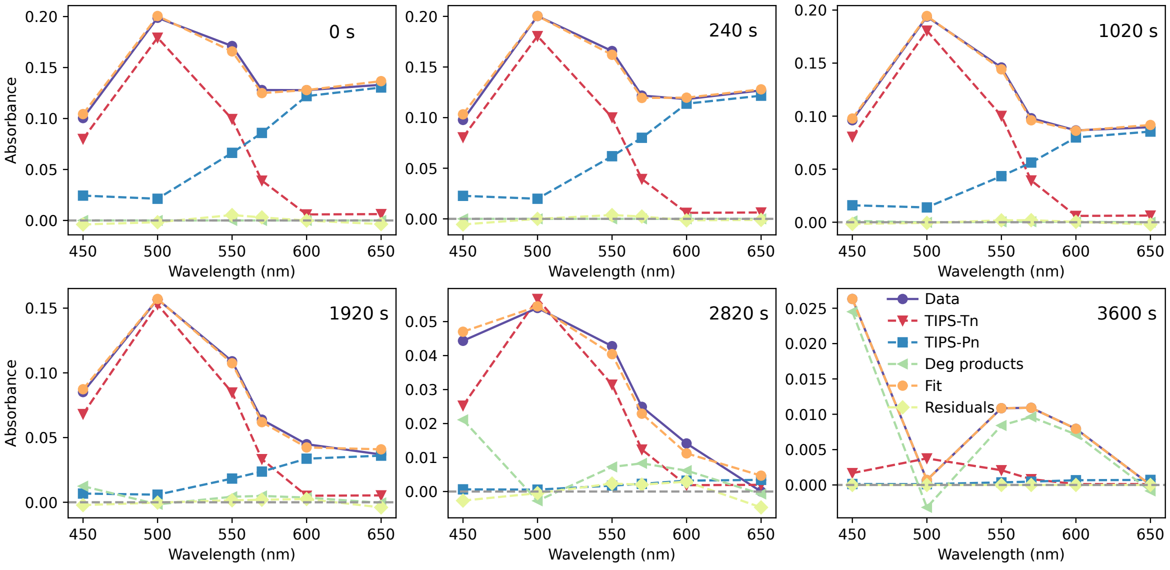

(d) 1:0.9 TIPS-Tn:TIPS-Pn

Figure S9: (Continued)

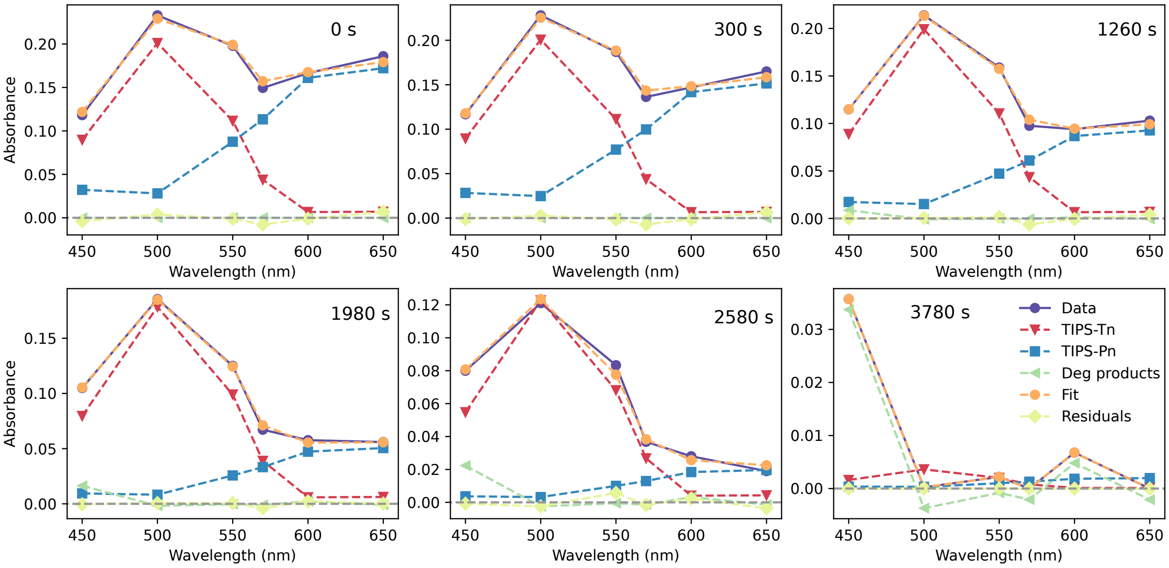

(e) 1:1.1 TIPS-Tn:TIPS-Pn

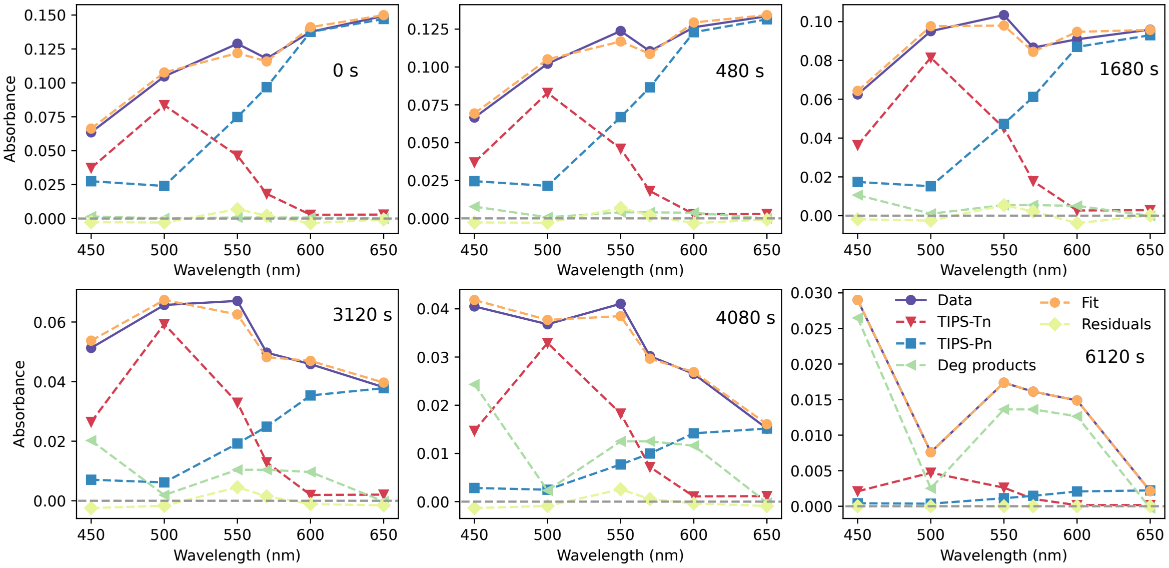

(f) 1:2.1 TIPS-Tn:TIPS-Pn

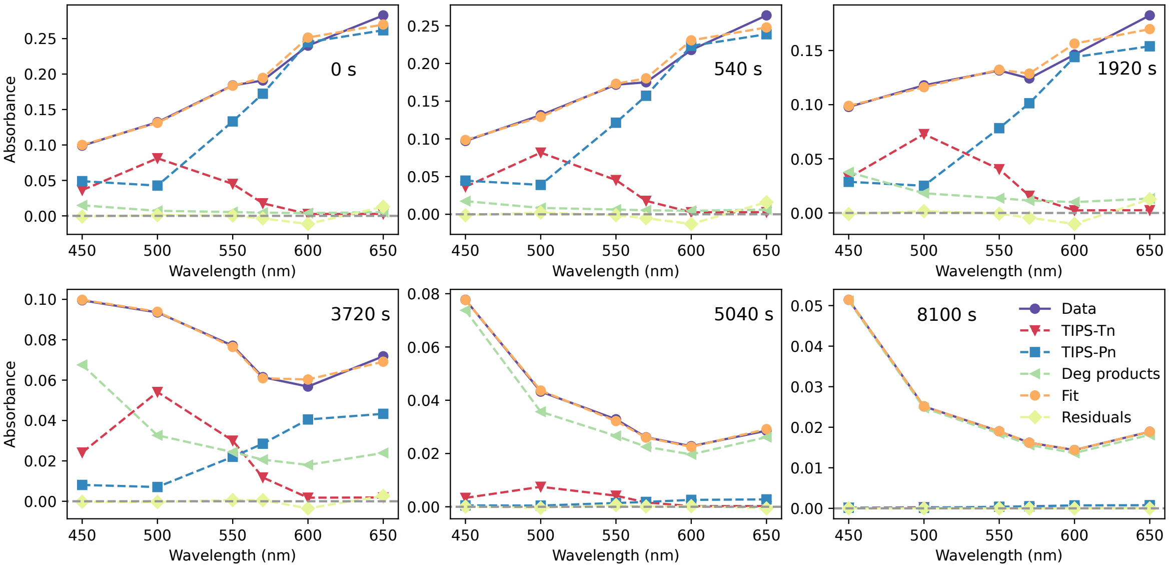

(g) 1:4.2 TIPS-Tn:TIPS-Pn

Figure S9: (Continued)

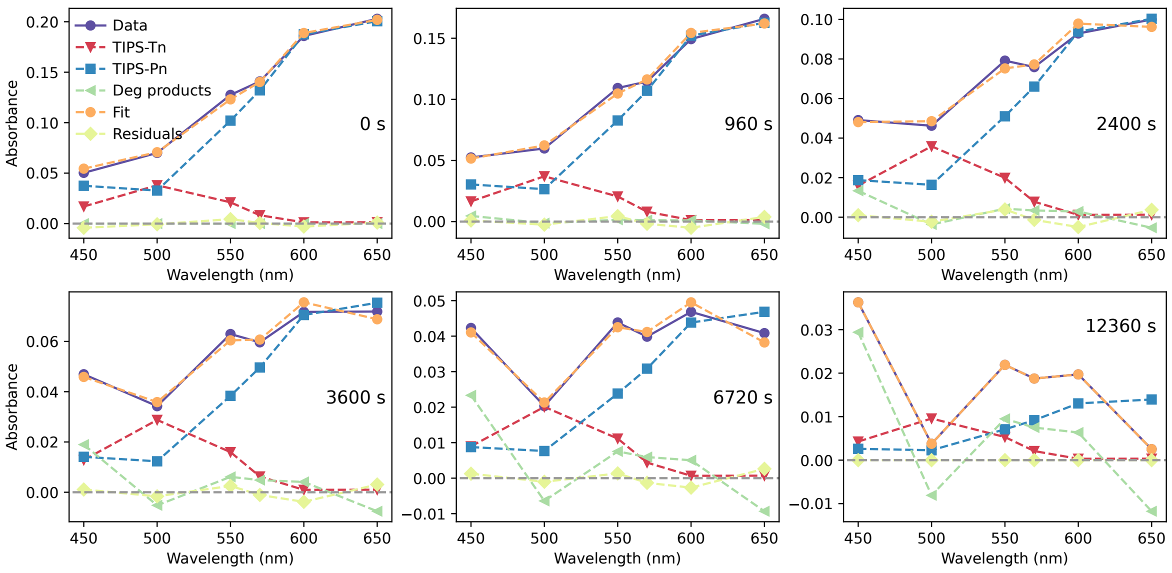

(h) 1:5.7 TIPS-Tn:TIPS-Pn

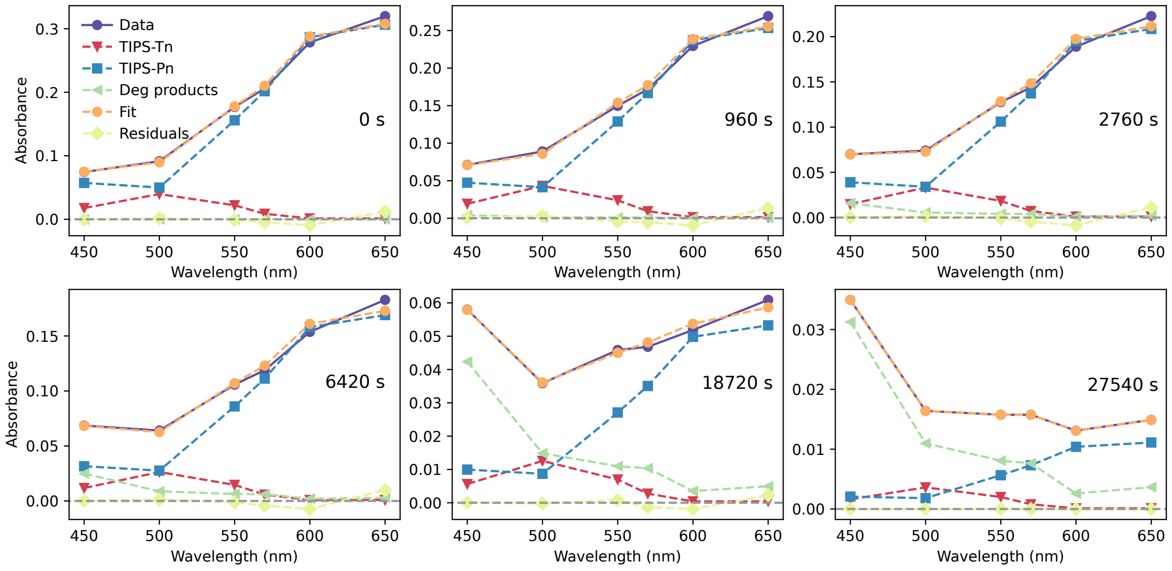

(i) 1:10.0 TIPS-Tn:TIPS-Pn

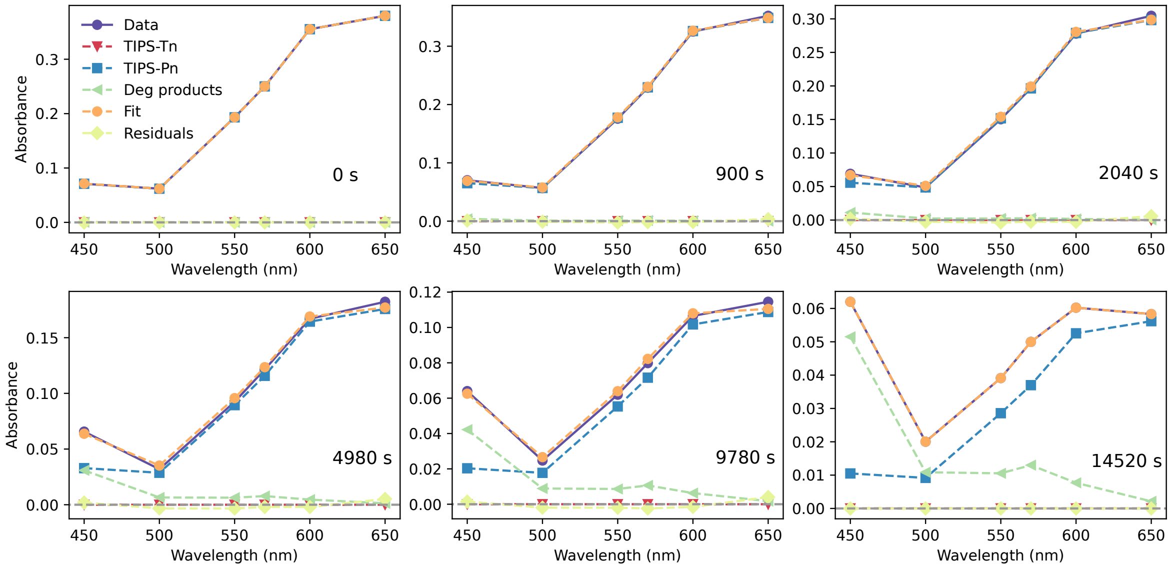

(j) 0:1 TIPS-Tn:TIPS-Pn

Figure S9: (Continued)

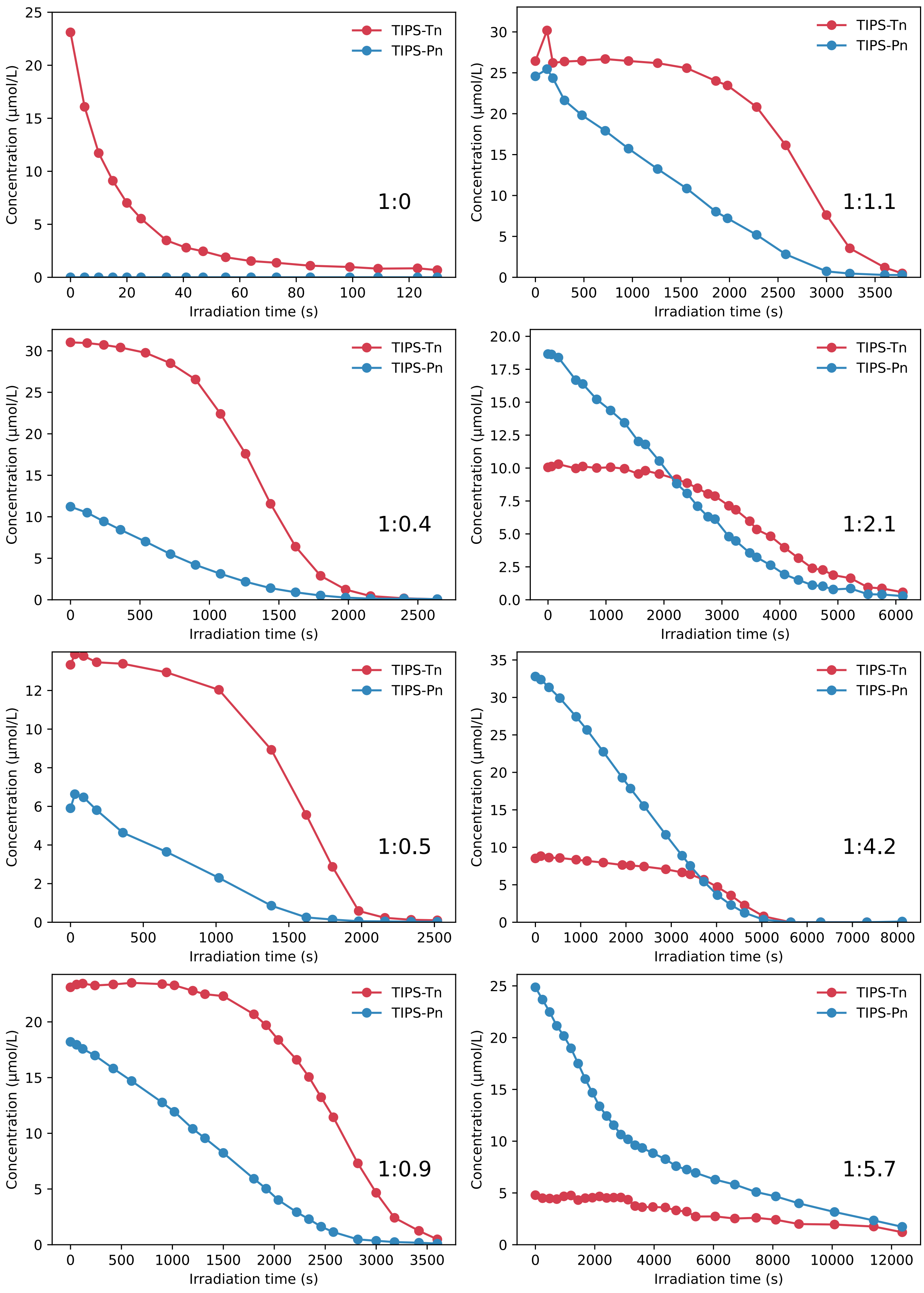

Figure S10: Fitted concentration of TIPS-Tn and TIPS-Pn vs irradiation time for select TIPS-Tn:TIPS-Pn blend NP samples. One example is shown for each mass ratio.

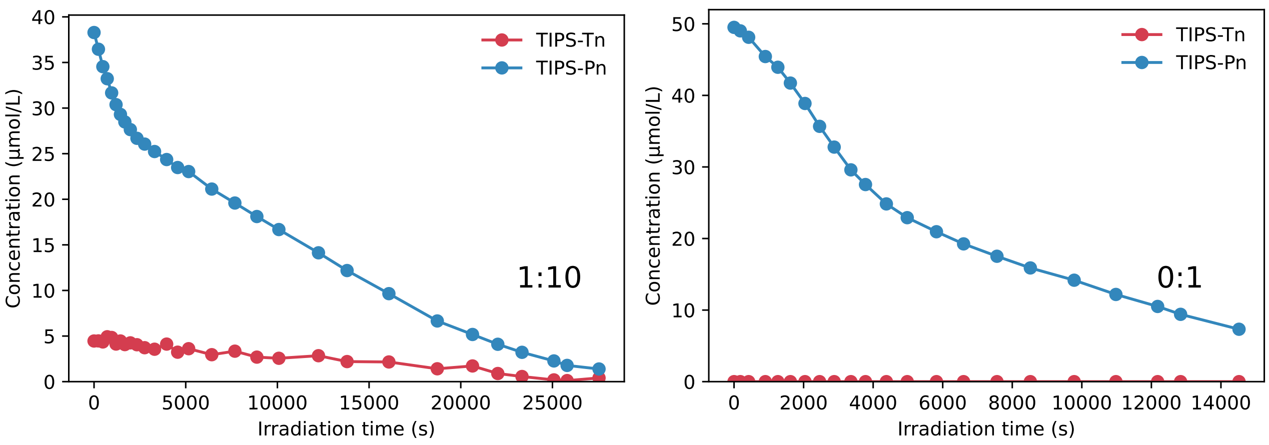

Figure S10: (Continued) Fitted concentration of TIPS-Tn and TIPS-Pn vs irradiation time for select TIPS-Tn:TIPS-Pn blend NP samples. One example is shown for each mass ratio.

S4 Excitation Rate Calculations

To model the photodegradation of TIPS-Tn and TIPS-Pn NPs, the excitation rate of the respective molecules was determined using the power of of the irradiation source and the absorbance of the sample. Samples in a 1 cm quartz cuvette were irradiated with a 1.3 cm radius spot from a Xenon lamp with spectrum given in Figure S12. Given the radius of the excitation spot and the size of the irradiated face of the cuvette, we calculated the excitation area, i.e. the overlap of the excitation spot with the cuvette, as indicated in Figure S11.

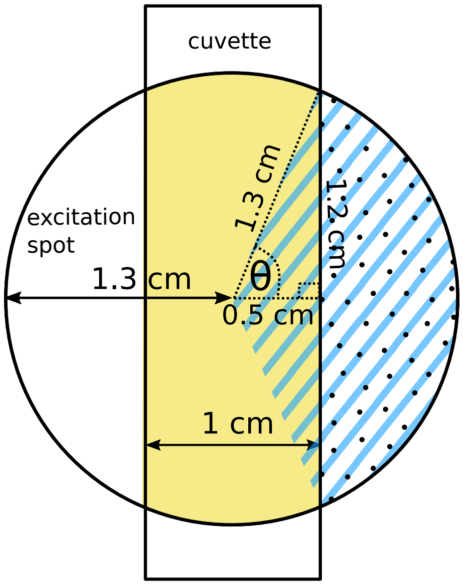

Figure S11: Excitation spot and cuvette overlap (yellow area)

If r is the radius of the excitation spot and θ is as indicated in Figure S11, then

$$\cos(\theta) = \frac{0.5\ \text{cm}}{1.3\ \text{cm}} \tag{S7}$$

$$\therefore \theta = 1.176\ \text{rad} \tag{S8}$$

$$\begin{aligned}\text{area of sector (stripes)} &= \frac{1}{2} \times (2\theta) \times r^2 \\ &= 1.176 \times 1.3^2 = 1.99\ \text{cm}^2 \end{aligned} \tag{S9}$$

$$\begin{aligned}\text{area of segment (spots)} &= \text{area of sector} - \frac{1}{2} \times 0.5 \times (2 \times 1.2) \\ &= 1.39\ \text{cm}^2 \end{aligned} \tag{S10}$$

$$\begin{aligned}\text{area of excitation spot} &= \pi r^2 = \pi \times 1.3^2 \\ &= 5.31\ \text{cm}^2 \end{aligned} \tag{S11}$$

$$\begin{aligned}\therefore \text{overlap area (yellow)} &= \text{area of excitation spot} - 2 \times \text{area of segment} \\ &= 2.53\ \text{cm}^2. \end{aligned} \tag{S12}$$

The power measured at the center of the sample on a 1 cm radius power meter was 230 mW, so

$$\begin{aligned}\text{power measured} &= 230 \pm 10\ \text{mW} \\ \text{power metre radius} &= 1\ \text{cm} \\ \text{power metre area} &= \pi \times \text{power metre radius}^2 = \pi\ \text{cm}^2 \\ \therefore \text{power intensity} &= 73.21\ \text{mW/cm}^2 \\ \therefore \text{power incident on sample} &= \text{power intensity} \times \text{overlap area} \\ &= 73.21\ \text{mW/cm}^2 \times 2.53\ \text{cm}^2 \\ &= 186\ \text{mW} = 0.186\ \text{J/s}. \end{aligned} \tag{S13}$$

Given the total incident power we can plot the excitation spectrum in terms of J/s/nm as in Figure S12:

$$\text{total incident energy}(\lambda) = \text{F}(\lambda) \times \frac{0.186}{\int \text{F}(\lambda)\text{d}\lambda}, \tag{S14}$$

where $\text{F}(\lambda)$ is the spectrum of the Xenon lamp (e.g. in counts or arbitrary units). Equation S14 is effectively setting the area under the Xenon lamp spectrum to be equal to the total power incident on the sample (0.186 J/s). The incident excitation energy was converted to the number of incident photons:

$$\text{photon energy}(\lambda) = \frac{hc}{\lambda} \tag{S15}$$

$$\text{number of incident photons}(\lambda) = \frac{\text{total incident energy}(\lambda)}{\text{photon energy}(\lambda)} \tag{S16}$$

Then, given the full absorbance spectrum of the sample (as measured by a spectrophotometer), the number of photons absorbed and hence the concentration of excitons generated per second, or the excitation

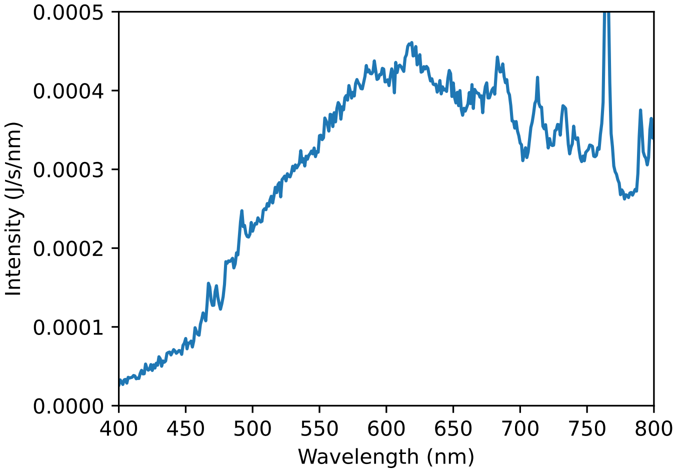

Figure S12: Excitation spectrum of Xenon lamp with a total incident power of 186 mW. The intensity plotted is equivalent to the total incident energy in Equation S14 (the area under the curve is 0.186 J/s).

rate, was calculated:

$$\text{photons absorbed}(\lambda) = (1 - 10^{-\text{absorbance}(\lambda)}) \times \text{no. incident photons}(\lambda) \tag{S17}$$

$$\text{total photons absorbed} = \sum_{\lambda} \text{photons absorbed}(\lambda) = \text{total excitons generated} \tag{S18}$$

$$\begin{aligned} \text{excitation rate} &= \frac{\text{total excitons generated}}{\text{excitation volume}} \\ &= \frac{\text{total excitons generated}}{\text{overlap area} \times \text{pathlength}} \\ &= \frac{\text{total excitons generated}}{N_{\mathrm{A}} \times 2.53 \times 10^{-3}} \ \text{mol/L/s} \end{aligned} \tag{S19}$$

The excitation rate constant k_{excite} (i.e. in units of /s) was determined as

$$\begin{aligned} \text{excitation rate} &= k_{\text{excite}} \times [\mathrm{S}_0](0) \\ \therefore k_{\text{excite}} &= \frac{\text{excitation rate}}{[\mathrm{S}_0](0)}, \end{aligned} \tag{S20}$$

where $[\mathrm{S}_0](0)$ is the initial ground state concentration of the NPs (i.e. at 0 irradiation time). For samples comprising both TIPS-Pn and TIPS-Tn, their excitation rates were calculated individually using their respective absorption components, yielding two individual excitation rates, one for TIPS-Pn and one for TIPS-Tn. We assumed here that every incident photon absorbed leads to an exciton generated. In reality a small amount of the absorbance spectrum is due to scattering, so these rate constants are slightly overestimated.

S5 Reproducibility

Several photodegradation experiments for neat TIPS-Tn and neat TIPS-Pn NPs at different concentrations and levels of oxygen were shown previously.[11] No trend with either oxygen content or NP concentration was observed, indicating oxygen was in excess for the photodegradation reactions. Replicates experiments were also carried out for 1:0.4 TIPS-Tn:TIPS-Pn NPs (Figure S13) and 1:0.9 TIPS-Tn:TIPS-Pn NPs (Figure S14). As with the neat NPs, the photodegradation of both blends is independent of concentration, and shows overall good reproducibility. The TIPS-Pn concentrations of the 23 µM 1:0.9 TIPS-Tn:TIPS-Pn NPs appear to be an outlier, which may have arisen from variation in excitation power, the NP size, NP morphology, and/or degree of scattering. This sample was excluded from the modeling in Section S6.3.

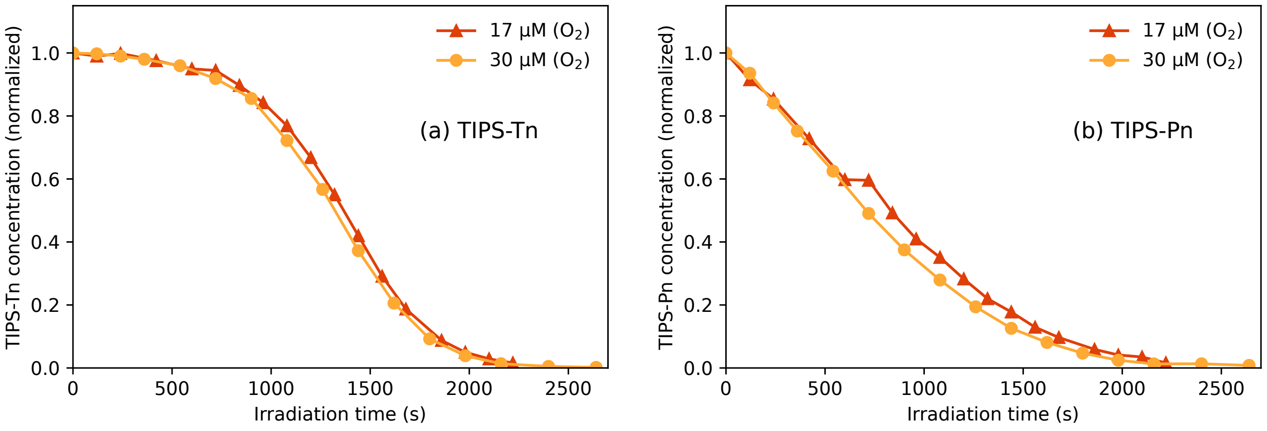

Figure S13: Normalized concentrations of (a) TIPS-Tn and (b) TIPS-Pn vs irradiation time in 1:0.4 TIPS-Tn:TIPS-Pn NPs at two different concentrations. The legend indicates the TIPS-Tn concentration.

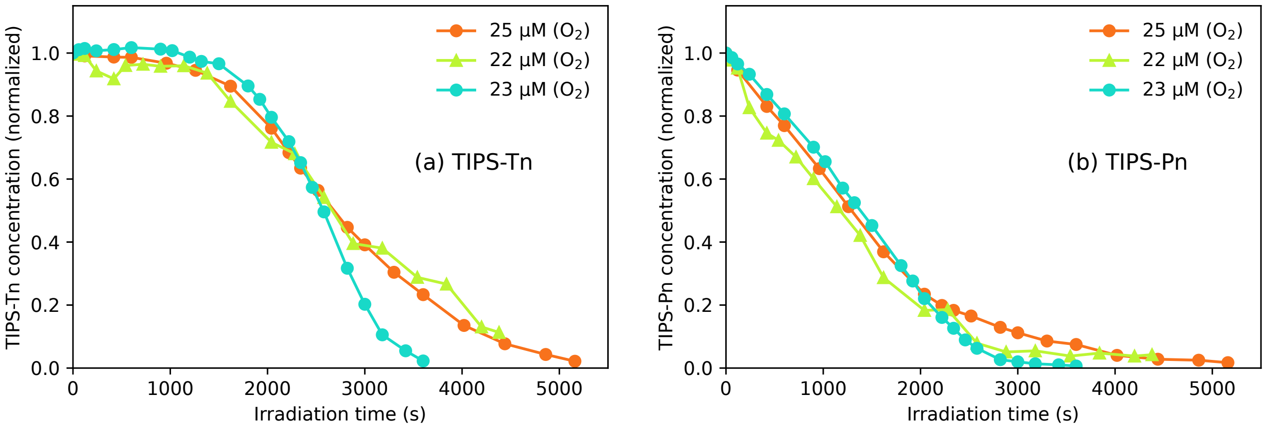

Figure S14: Normalized concentrations of (a) TIPS-Tn and (b) TIPS-Pn vs irradiation time in 1:0.9 TIPS-Tn:TIPS-Pn NPs at three different concentrations. The legend indicates the TIPS-Tn concentration.

S6 Kinetic Modeling

S6.1 Neat TIPS-Tn NP Degradation

S6.1.1 Model 1

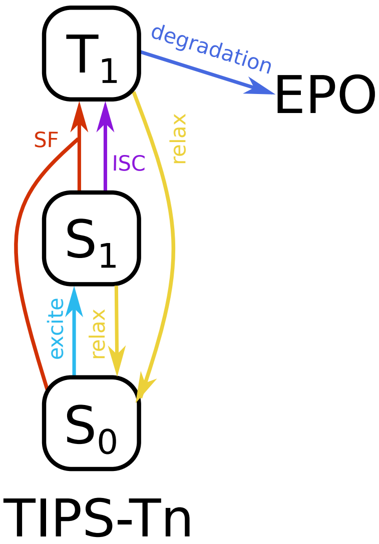

Figure S15: Model 1 for the photodegradation of neat TIPS-Tn NPs. EPO indicates endoperoxides or other products of photodegradation.

To describe the photodegradation of the neat TIPS-Tn NP system we first used the simple model shown in Figure S15, which we label Model 1. The ground-state molecules of TIPS-Tn (with concentration $[\mathrm{S_0}]$) are excited with an excitation rate constant of k_{excite} to the singlet excited state. The singlet excited-state population (with concentration $[\mathrm{S_1}]$) can either decay back to the ground state with rate constant $k_{\mathrm{S_1}}$, undergo SF with a neighboring molecule in the $\mathrm{S_0}$ state with rate constant k_{SF} to give two triplet excitons, or undergo ISC with rate constant k_{ISC} to give one triplet exciton. The triplet exciton population (with concentration $[\mathrm{T_1}]$) can decay to the $\mathrm{S_0}$ state with rate constant $k_{\mathrm{T_1}}$, or undergo a degradation reaction with rate constant k_{deg}. These processes can be represented by the following system of differential equations,

$$\frac{\mathrm{d}[\mathrm{S_0}]}{\mathrm{d}t} = -k_{\mathrm{excite}}[\mathrm{S_0}] - k_{\mathrm{SF}}[\mathrm{S_0}][\mathrm{S_1}] + k_{\mathrm{S_1}}[\mathrm{S_1}] + k_{\mathrm{T_1}}[\mathrm{T_1}], \tag{S21a}$$

$$\frac{\mathrm{d}[\mathrm{S_1}]}{\mathrm{d}t} = k_{\mathrm{excite}}[\mathrm{S_0}] - k_{\mathrm{SF}}[\mathrm{S_0}][\mathrm{S_1}] - k_{\mathrm{ISC}}[\mathrm{S_1}] - k_{\mathrm{S_1}}[\mathrm{S_1}], \tag{S21b}$$

$$\frac{\mathrm{d}[\mathrm{T_1}]}{\mathrm{d}t} = 2k_{\mathrm{SF}}[\mathrm{S_0}][\mathrm{S_1}] + k_{\mathrm{ISC}}[\mathrm{S_1}] - k_{\mathrm{T_1}}[\mathrm{T_1}] - k_{\mathrm{deg}}[\mathrm{T_1}]. \tag{S21c}$$

For neat TIPS-Tn NPs, the excitation rate, k_{excite}, can be calculated as described in Section S4. We have previously modeled the photophysics of neat TIPS-Tn NPs under $\mathrm{N_2}$ with transient absorption spectroscopy.[11] The decay of singlet excitons to the ground state, $k_{\mathrm{S_1}}$, was found to be to be $8.5\times10^{7}\,\mathrm{s^{-1}}$ (time constant 11.7 ns), and the decay of triplet excitons to the ground state, $k_{\mathrm{T_1}}$, was found to be $8.1\times10^{7}\,\mathrm{s^{-1}}$ (time constant 12.3 ns). In transient absorption experiments the ground-state population, $[\mathrm{S_0}]$, is effectively constant and SF is pseudo-first order. The pseudo-first order rate constant of SF in TIPS-Tn NPs was found to be $1.02\times10^{11}\,\mathrm{s^{-1}}$ (time constant of 9.8 ps). Since the ground-state population in the photodegradation experiments is not constant, SF can not be modeled as first order. The equivalent second order (bimolecular) SF rate can be determined using the time zero (undegraded) concentration,

$[S_0](0)$, using

$$k_{SF}[S_0](0) = 1.02 \times 10^{11}\ \mathrm{s}^{-1}$$
$$\therefore k_{SF} = \frac{1.02 \times 10^{11}}{[S_0](0)}\ \mathrm{Lmol^{-1}s^{-1}}. \tag{S22}$$

Finally, an upper bound of the rate constant of ISC was previously determined to be $5.1\times10^6\,\mathrm{s}^{-1}$ (time constant of 197 ns). However in the presence of oxygen, TIPS-Tn triplet excitons can also be formed through the sensitization of singlet oxygen.[11] Assuming oxygen is in excess (Section S5), this reaction can be described by a pseudo-first order process producing one triplet exciton from one singlet exciton, analogous to ISC. Hence rather than fix k_{ISC} in the modeling of photodegradation under oxygen, we allowed it to vary to account for both ISC and oxygen sensitization.

The remaining unknown rate constant in Model 1 is the degradation rate constant, k_{deg}. This value was also varied to allow the model to fit to the degradation data, resulting in the fit in Figure S16. The fitted degradation rate constant to the replicate in Figure S16 is $3.7\times10^6\,\mathrm{s}^{-1}$ (time constant 270 ns), and the ISC plus O_2 sensitization rate (k_{ISC}) is $7.6\times10^7\,\mathrm{s}^{-1}$ (time constant 13 ns).

Note that for all the modeling in this study, where replicates of photodegradation experiments existed, each replicate was fit individually. Fitting an average of the experimental replicates would not be reasonable as each has different initial concentrations and excitation powers. All kinetic models in this study are stiff, in that many of the processes included (e.g. SF) are significantly faster than the timescale the data is measured over. Stiff systems are unstable when using simple numerical integration methods, but such problems are prevalent in many areas (e.g. atmospheric chemistry), and many algorithms have been developed that are well suited to solve them.[12] To avoid instabilities that arise when fitting stiff systems we used an implicit Runge–Kutta method for the numerical integration. The integration and fitting was performed in Python, where the ODEs were solved numerically using the package `solve_ivp` with the method `Radau`, which is a fifth order implicit Runge–Kutta method of the Radau IIA family.[13,14] Residuals were minimized using the function `minimize` from the LMFIT package with the Nelder–Mead algorithm.[15,16]

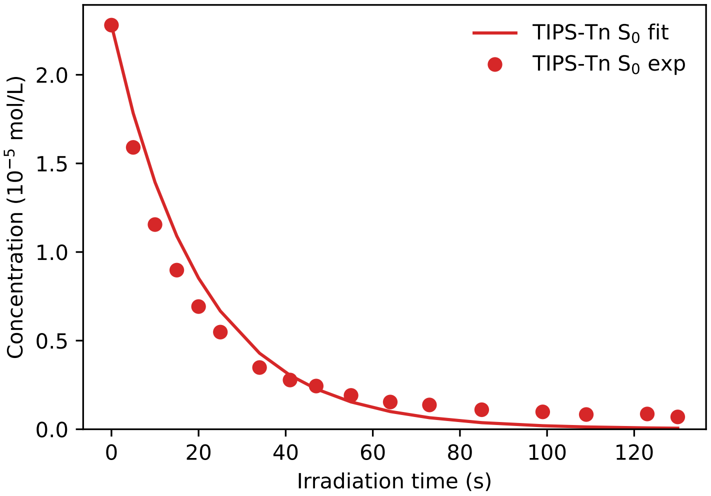

Figure S16: Fit of Model 1 to a replicate of the photodegradation of neat TIPS-Tn NPs.

The fit in Figure S16 overestimates the photodegradation of TIPS-Tn at late times. This discrepancy is consistent across each replicate of neat TIPS-Tn NPs. To quantify the difference in quality of fits between models we calculated the total root mean squared error ($\mathrm{RMSE_T}$) of the fit across all replicates, normalized to the initial TIPS-Tn concentration. For M replicate experiments, each with N_i number of

data points, the $\mathrm{RMSE_T}$ is

$$\mathrm{RMSE_T} = \sqrt{\frac{1}{M}\sum_{i=1}^{M}\left(\frac{1}{N_i}\sum_{j=1}^{N_i}\frac{(\mathrm{fit}_{i,j}-\mathrm{expt}_{i,j})^2}{\mathrm{expt}_{i,1}^2}\right)}, \tag{S23}$$

where $\mathrm{fit}_{i,j}$ and $\mathrm{expt}_{i,j}$ are the fitted and experimental concentrations of replicate i at data point j, respectively, and $\mathrm{expt}_{i,1}$ is the initial concentration of replicate i (i.e. at time zero). $\mathrm{RMSE_T}$ was 0.039 ± 0.009 across 5 replicate experiments ($M = 5$), indicating that Model 1 is not the best representation of the processes in this system. This result can be explained by considering that the NPs are in the solid state, rather than solution. In solution, SF is a result of collisions between molecules. As the population of TIPS-Tn is depleted through photodegradation in solution, fewer collisions occur, resulting in less SF, less triplet formation, and therefore less degradation over time. This behavior is expressed through the SF term, $k_{\mathrm{SF}}[\mathrm{S_0}][\mathrm{S_1}]$, which becomes smaller as $[\mathrm{S_0}]$ and $[\mathrm{S_1}]$ decrease. In a solid-state system, SF occurs between fixed neighboring molecules rather than via collisions between molecules, so solely using the second-order term $k_{\mathrm{SF}}[\mathrm{S_0}][\mathrm{S_1}]$ does not adequately describe the deceleration of SF. As the population of TIPS-Tn decreases in the NP due to photodegradation, molecules lose their nearest neighbors and can no longer undergo SF. Excitons on these molecules either have to migrate away or remain trapped until they relax to the ground state. Hence triplet formation, and therefore photodegradation, slows down much more than Model 1 accounts for. To rectify this deficiency, we used an alternate model that uses two populations of molecules to describe SF in the solid state, as indicated in Model 2 in Figure S17.

S6.1.2 Model 2

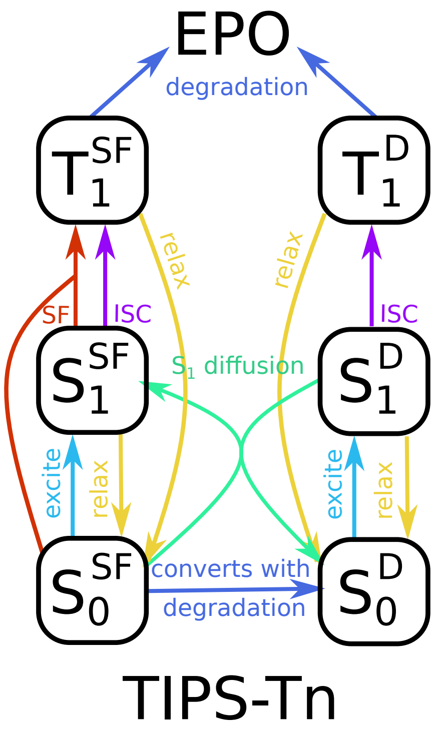

Figure S17: Model 2 for the degradation of neat TIPS-Tn NPs, including a diffusion population that is generated as SF sites degrade.

As TIPS-Tn molecules degrade, remaining molecules have fewer neighbors and become less able to undergo SF. Excitons on these molecules have to diffuse to other sites to undergo SF, or are otherwise

trapped and relax to the ground state. In lieu of the necessary information to fit a full diffusion model, a common, simpler method is to split molecules into two populations:[4,17] molecules that are able to undergo SF, which we call SF sites and denote with the superscript SF ($S_{0/1}^{SF}$ or T_1^{SF}), and molecules that cannot, which we call diffusion sites and denote with the superscript D ($S_{0/1}^{D}$ or T_1^{D}). Excitons on diffusion sites are able to migrate to SF sites with rate constant k_D. Note that this represents an average rate at which excitons migrate from diffusion sites to SF sites. Some excitons may migrate over just one molecule before they undergo SF, whereas others may migrate over 10s of molecules. This method of describing diffusion has been previously employed to model ultrafast TA data in amorphous pentacene and tetracene derivatives.[4,17] We have also used more thorough molecular-scale Monte Carlo simulations to describe diffusion-limited SF in these amorphous NPs in past studies, but this level of detail is unnecessary here, and conclusions from the Monte Carlo simulations were consistent with those of the simpler model.[18]

We consider the NPs prior to degradation to initially consist entirely of SF sites. As the NPs degrade, some SF sites become isolated and are converted into diffusion sites with probability $p[S_0^{SF}]$. This can be represented by the system of differential equations

$$\frac{d[S_0^{SF}]}{dt} = -k_{excite}[S_0^{SF}] + k_{S_1}[S_1^{SF}] + k_{T_1}[T_1^{SF}] - k_D[S_1^D][S_0^{SF}] - k_{SF}[S_0^{SF}][S_1^{SF}] - pk_{deg}[T_1^{SF}][S_0^{SF}] \quad (S24a)$$

$$\frac{d[S_0^{D}]}{dt} = -k_{excite}[S_0^{D}] + k_{S_1}[S_1^{D}] + k_{T_1}[T_1^{D}] + k_D[S_1^D][S_0^{SF}] + pk_{deg}[T_1^{SF}][S_0^{SF}], \quad (S24b)$$

$$\frac{d[S_1^{SF}]}{dt} = k_{excite}[S_0^{SF}] - k_{S_1}[S_1^{SF}] + k_D[S_1^D][S_0^{SF}] - k_{ISC}[S_1^{SF}] - k_{SF}[S_0^{SF}][S_1^{SF}], \quad (S24c)$$

$$\frac{d[S_1^{D}]}{dt} = k_{excite}[S_0^{D}] - k_{S_1}[S_1^{D}] - k_D[S_1^D][S_0^{SF}] - k_{ISC}[S_1^{D}], \quad (S24d)$$

$$\frac{d[T_1^{SF}]}{dt} = 2k_{SF}[S_0^{SF}][S_1^{SF}] + k_{ISC}[S_1^{SF}] - k_{T_1}[T_1^{SF}] - k_{deg}[T_1^{SF}], \quad (S24e)$$

$$\frac{d[T_1^{D}]}{dt} = k_{ISC}[S_1^{D}] - k_{T_1}[T_1^{D}] - k_{deg}[T_1^{D}]. \quad (S24f)$$

The fit of Model 2 to the neat TIPS-Tn photodegradation data is shown in Figure S18. The values k_{deg}, k_D, k_{ISC}, and p were allowed to vary to fit the data, and the other rate constants were fixed as described for Model 1.

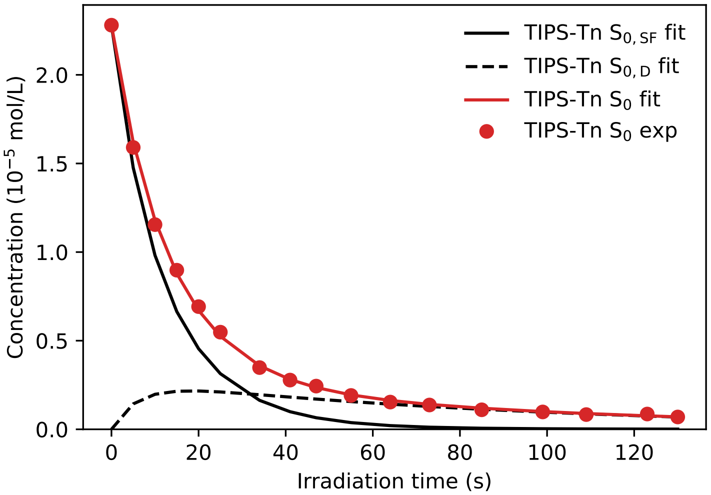

Figure S18: Fit of Model 2 to the photodegradation of a sample of neat TIPS-Tn NPs. The contributions of the SF site population and the diffusion site population are shown as full and dashed black lines, respectively, and the total S_0 population is in red.

Model 2 fits the photodegradation well. The contributions due to the SF and diffusion sites are also shown in Figure S18, where it can be seen that the diffusion sites become relatively more populated as the SF sites degrade to zero. The diffusion sites are relatively more long-lived, which accounts for the tail that Model 1 failed to describe. These sites are only able to reach the triplet state and subsequently photodegrade via ISC or singlet oxygen sensitization (k_{ISC}), which is significantly slower than SF. Models 1 and 2 were fit for the five replicate photodegradation experiments of neat TIPS-Tn NPs, with Model 2 fitting better in each case. For Model 2 (with diffusion) the $\mathrm{RMSE_T}$ was 0.009 ± 0.004, which is almost five times lower than the $\mathrm{RMSE_T}$ for Model 1 (without diffusion), which was 0.039 ± 0.009.

The fitted degradation rate constant using Model 2 is $(4.8 \pm 0.3) \times 10^6\,\mathrm{s^{-1}}$ (time constant of 210±15 ns), where the error was determined from the average fitted rate constant across the five different replicates. This psuedo-first order rate constant is similar to that previously measured for photodegradation in toluene solution, which had a maximum value of $5.1 \times 10^6\,\mathrm{s^{-1}}$ (time constant of 196 ns),[11] though it should be noted that this process was treated as bimolecular in solution, and was dependent on the singlet oxygen concentration, which we do not explicitly model for the NPs.

k_{ISC} is fit as $(6 \pm 1) \times 10^7\,\mathrm{s^{-1}}$ (time constant of 18±3 ns). This value includes both the intrinsic ISC of TIPS-Tn and the sensitization of T_1 by oxygen. The upper bound on the rate constant of ISC for TIPS-Tn in toluene under N_2 was previously found to be $5.1 \times 10^6\,\mathrm{s^{-1}}$ (time constant of 197 ns). If we take $k_{\mathrm{ISC}} = k_{\mathrm{ISC,N_2}} + k_1$, where k_1 is the rate constant of sensitization by oxygen, then the lower bound on k_1 is $5.2 \times 10^7\,\mathrm{s^{-1}}$, corresponding to a time constant of 19 ns. This value is similar to the 24 ns time constant measured for TIPS-Tn in toluene solution.[11]

The diffusion rate constant is fit as essentially negligible, with an initial (pseudo-first-order) value of $1.3 \times 10^{-3}\,\mathrm{s^{-1}}$ (time constant of $\sim$800 s). This indicates that despite their name, the singlet excitons on the diffusion sites do not diffuse, and instead either undergo ISC or decay to the ground state. These molecules are thus potentially better described as isolated, or trapped sites, rather than diffusion sites. This does not necessarily mean exciton diffusion does not occur in the NPs, merely that it is fast enough to not be rate limiting in the SF process.

Finally the constant p representing the probability of SF sites converting to diffusion sites was fit as $9000 \pm 3000\,\mathrm{M^{-1}}$. For a concentration of 2.38×10^{-5} M, the proportion is 0.22 ± 0.07. This indicates that initially, for each degradation event on a SF site, there is a 22% chance that the remaining neighboring molecule will no longer be able to undergo SF, and become a diffusion site.

Note that this is a simplified model of the photophysics in the system. In reality, there are additional decay channels through excimer formation,[19] the decay or recombination of the triplet pair intermediate,[20] and further interactions with oxygen.[11] All these process effectively act to speed up the return to the ground state, so they are equivalent here to having a faster triplet decay, or a larger excitation rate. This would in turn lead to a faster degradation rate. Hence is it possible that the degradation rate is being underestimated using the simple model here. However, if the same assumptions are made in the subsequent modeling of the blend systems, they are unlikely to effect the trends found there.

S6.2 Neat TIPS-Pn NP Degradation

S6.2.1 Model 3

To model the photodegradation of neat TIPS-Pn NPs, we initially used the same method as for TIPS-Tn, with the exception of photodegradation occurring through the singlet excited state, rather than triplet (Figure S19).[11] This can be expressed with the system of differential equations

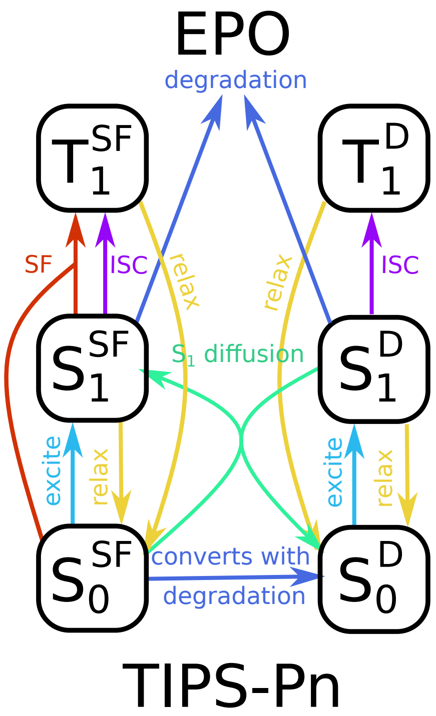

Figure S19: Model 3, for degradation of neat TIPS-Pn with diffusion.

$$\frac{\mathrm{d}[\mathrm{S}_0^{\mathrm{SF}}]}{\mathrm{d}t} = -k_{\mathrm{excite}}[\mathrm{S}_0^{\mathrm{SF}}] + k_{\mathrm{S}_1}[\mathrm{S}_1^{\mathrm{SF}}] + k_{\mathrm{T}_1}[\mathrm{T}_1^{\mathrm{SF}}] - k_{\mathrm{D}}[\mathrm{S}_1^{\mathrm{D}}][\mathrm{S}_0^{\mathrm{SF}}] - k_{\mathrm{SF}}[\mathrm{S}_0^{\mathrm{SF}}][\mathrm{S}_1^{\mathrm{SF}}] - pk_{\mathrm{deg}}[\mathrm{S}_1^{\mathrm{SF}}][\mathrm{S}_0^{\mathrm{SF}}], \quad \text{(S25a)}$$

$$\frac{\mathrm{d}[\mathrm{S}_0^{\mathrm{D}}]}{\mathrm{d}t} = -k_{\mathrm{excite}}[\mathrm{S}_0^{\mathrm{D}}] + k_{\mathrm{S}_1}[\mathrm{S}_1^{\mathrm{D}}] + k_{\mathrm{T}_1}[\mathrm{T}_1^{\mathrm{D}}] + k_{\mathrm{D}}[\mathrm{S}_1^{\mathrm{D}}][\mathrm{S}_0^{\mathrm{SF}}] + pk_{\mathrm{deg}}[\mathrm{S}_1^{\mathrm{SF}}][\mathrm{S}_0^{\mathrm{SF}}], \quad \text{(S25b)}$$

$$\frac{\mathrm{d}[\mathrm{S}_1^{\mathrm{SF}}]}{\mathrm{d}t} = k_{\mathrm{excite}}[\mathrm{S}_0^{\mathrm{SF}}] - k_{\mathrm{S}_1}[\mathrm{S}_1^{\mathrm{SF}}] + k_{\mathrm{D}}[\mathrm{S}_1^{\mathrm{D}}][\mathrm{S}_0^{\mathrm{SF}}] - k_{\mathrm{ISC}}[\mathrm{S}_1^{\mathrm{SF}}] - k_{\mathrm{SF}}[\mathrm{S}_0^{\mathrm{SF}}][\mathrm{S}_1^{\mathrm{SF}}] - k_{\mathrm{deg}}[\mathrm{S}_1^{\mathrm{SF}}], \quad \text{(S25c)}$$

$$\frac{\mathrm{d}[\mathrm{S}_1^{\mathrm{D}}]}{\mathrm{d}t} = k_{\mathrm{excite}}[\mathrm{S}_0^{\mathrm{D}}] - k_{\mathrm{S}_1}[\mathrm{S}_1^{\mathrm{D}}] - k_{\mathrm{D}}[\mathrm{S}_1^{\mathrm{D}}][\mathrm{S}_0^{\mathrm{SF}}] - k_{\mathrm{ISC}}[\mathrm{S}_1^{\mathrm{D}}] - k_{\mathrm{deg}}[\mathrm{S}_1^{\mathrm{D}}], \quad \text{(S25d)}$$

$$\frac{\mathrm{d}[\mathrm{T}_1^{\mathrm{SF}}]}{\mathrm{d}t} = 2k_{\mathrm{SF}}[\mathrm{S}_0^{\mathrm{SF}}][\mathrm{S}_1^{\mathrm{SF}}] + k_{\mathrm{ISC}}[\mathrm{S}_1^{\mathrm{SF}}] - k_{\mathrm{T}_1}[\mathrm{T}_1^{\mathrm{SF}}] \quad \text{(S25e)}$$

$$\frac{\mathrm{d}[\mathrm{T}_1^{\mathrm{D}}]}{\mathrm{d}t} = k_{\mathrm{ISC}}[\mathrm{S}_1^{\mathrm{D}}] - k_{\mathrm{T}_1}[\mathrm{T}_1^{\mathrm{D}}]. \quad \text{(S25f)}$$

Based on previous transient absorption studies on TIPS-Pn NPs,[4,21] we fixed the rate of S_1 decay, k_{S_1}, as $8.3 \times 10^7\ \mathrm{s}^{-1}$ (time constant of 12 ns), the rate of T_1 decay, k_{T_1}, as $6.7 \times 10^7\ \mathrm{s}^{-1}$ (time constant of 15 ns), and k_{SF} as $2.6 \times 10^{11}\ \mathrm{s}^{-1}$ (time constant of 4 ps). k_{excite} was calculated as described in Section S4. k_{deg}, k_{D}, k_{ISC}, and p were varied to fit the data, resulting in the fit in Figure S20.

As demonstrated by Figure S20, this model is unable to reproduce the TIPS-Pn photodegradation. As with TIPS-Tn, the modeling was repeated for multiple replicates of the TIPS-Pn degradation, and the discrepancy was consistent across all samples ($\mathrm{RMSE_T}$ across seven replicates was 0.09 ± 0.01). Since the photodegradation pathway is through the S_1 state for TIPS-Pn, the diffusion site population instantly goes to zero and is unable to build up any appreciable population. Consequently, the photodegradation fit by this model is very linear, and is unable to capture the change in slope in the experimental data.

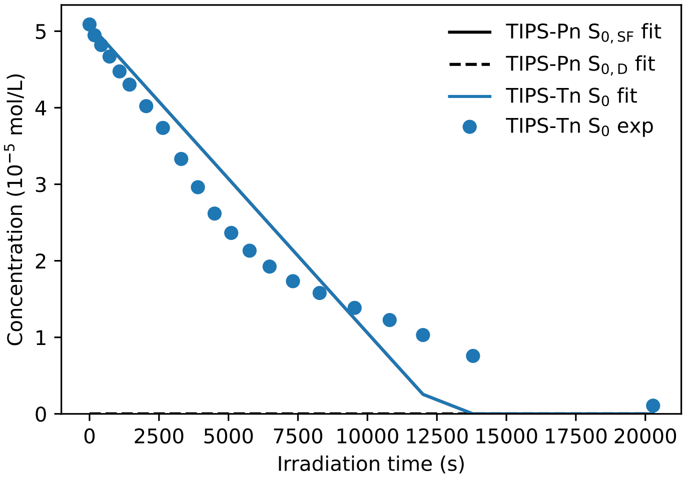

Figure S20: Fit of Model 3 to the photodegradation of a sample of neat TIPS-Pn NPs. The contributions of the SF site population and the diffusion site population are shown as solid and dashed black lines, respectively, and the total S_0 population is in blue. The diffusion population is zero for the entire timescale, so the total S_0 population is coincident with the SF population.

S6.2.2 Model 4

The TIPS-Pn photodegradation shows a change in slope over the irradiation time, which cannot be accounted for by diffusion. To reproduce this shape, we consider that there may be some disorder in the NPs such that some TIPS-Pn molecules are closer, or more accessible to oxygen than others. The rate of photodegradation, k_{deg}, may be better described by a broader distribution of rate constants, rather than a single value. To model this distribution in the simplest form, we split the molecules into two populations with independent photodegradation rates representing molecules that are closer to oxygen, and those that are further away. This treatment is somewhat analogous to modeling diffusion by splitting the population into SF and diffusion sites. The system is then described by two sets of Equations S25a–S25f, identical except for the rates of degradation, $k_{\text{deg},1}$ and $k_{\text{deg},2}$. We fit this to the data allowing $k_{\text{deg},1}$, $k_{\text{deg},2}$, k_{D}, k_{ISC}, and p to vary, along with the proportion of sites using each photodegradation rate constant. The resulting fit is given in Figure S21.

This method reproduces the photodegradation well. As with neat TIPS-Tn NPs, we fit the two possible models (Model 3 and Model 4) to each photodegradation replicate, and determined the difference in RMSE_{T}. For Model 3, RMSE_{T} was 0.09 ± 0.1, whereas for Model 4, RMSE_{T} was 0.012 ± 0.004, confirming that including a second population fits the neat TIPS-Pn photodegradation significantly better. However, some of the rate constants in Model 4 are fit with poor sensitivity. Without SF as a competitive pathway, photodegradation from the diffusion sites is rapid, and no appreciable diffusion site population is built up. The model is thus insensitive to the value of p and the diffusion rate constant. In other words, a diffusion site population is unnecessary for TIPS-Pn. Similarly, there is low sensitivity in the ISC rate constant, since a higher rate constant of ISC can be offset by a higher rate constant of photodegradation, resulting in the same quality of fit (because ISC just delays photodegradation by converting molecules to the triplet state, where they eventually relax to the ground state to be re-excited to the singlet excited state again). Therefore, to simplify the fitting, we constrained the proportion of diffusion sites and the diffusion rate constant to zero. Based on the similarity between the solution and NP ISC (plus oxygen sensitization) rate constants for TIPS-Tn, we constrained the TIPS-Pn ISC rate constant to the same as that measured for solution ($6.7 \times 10^7\,\text{s}^{-1}$, or time constant of 15 ns).

For the sample in Figure S21, 24% of molecules had a degradation rate constant of $7.9 \times 10^7\,\text{s}^{-1}$, and the remaining 76% had a degradation rate constant of $1.2 \times 10^7\,\text{s}^{-1}$. Potentially, the 76% of sites with a slower rate constant could represent an inner core population, and the 24% with a faster rate constant the

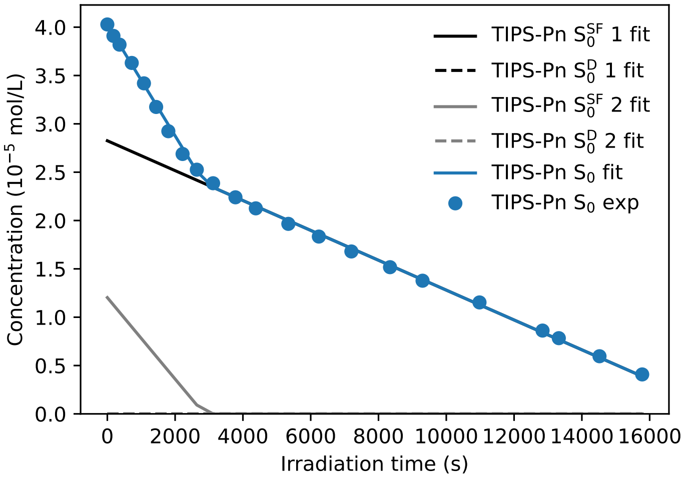

Figure S21: Fit of Model 4 to the photodegradation of neat TIPS-Pn NPs. The contributions of the SF site population and the diffusion site population are shown as solid and dashed lines, respectively. TIPS-Pn sites with degradation rates of $k_{\mathrm{deg},1}$ are denoted $\mathrm{S}_0^{\mathrm{SF/D}}$ 1 (shown in black), and degradation rates of $k_{\mathrm{deg},2}$ are denoted $\mathrm{S}_0^{\mathrm{SF/D}}$ 2 (shown in grey). The total S_0 population (sum of diffusion sites and SF sites for both degradation populations) is in blue. Note that the dashed lines (diffusion populations) are close to zero.

population of molecules on surface, indicated by the blue and purple circles in Figure S22, respectively. This would correspond to a surface layer approximately 1–2 TIPS-Pn molecules deep, depending on the NP density. Across 7 different neat TIPS-Pn samples, the average proportion of the faster degradation rate constant was 0.25 ± 0.05. The average faster degradation rate constant was $(7 \pm 1) \times 10^7\,\mathrm{s}^{-1}$ (time constant of 14 ns), and the average slower degradation rate constant was $(1.27 \pm 0.03) \times 10^7\,\mathrm{s}^{-1}$ (time constant of 80 ns).

It is unclear why multiple photodegradation rate constants are needed to describe TIPS-Pn, but not TIPS-Tn. Potentially because TIPS-Tn photodegrades much faster, and through the triplet state, it is less sensitive to deviations in morphology or proximity to oxygen.

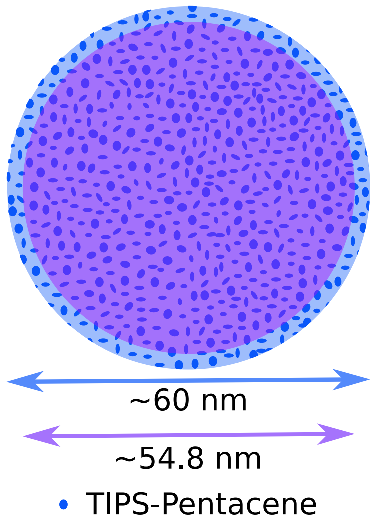

Figure S22: Cross section of the potential surface and core sites in TIPS-Pn NPs. The inner purple circle represents 76% of the NP volume, and outer blue remaining 26% of surface sites. TIPS-Pn molecules are to scale (shown in various orientations) but the density of molecules is not necessarily accurate.

S6.3 TIPS-Tn:TIPS-Pn Blend NP Degradation

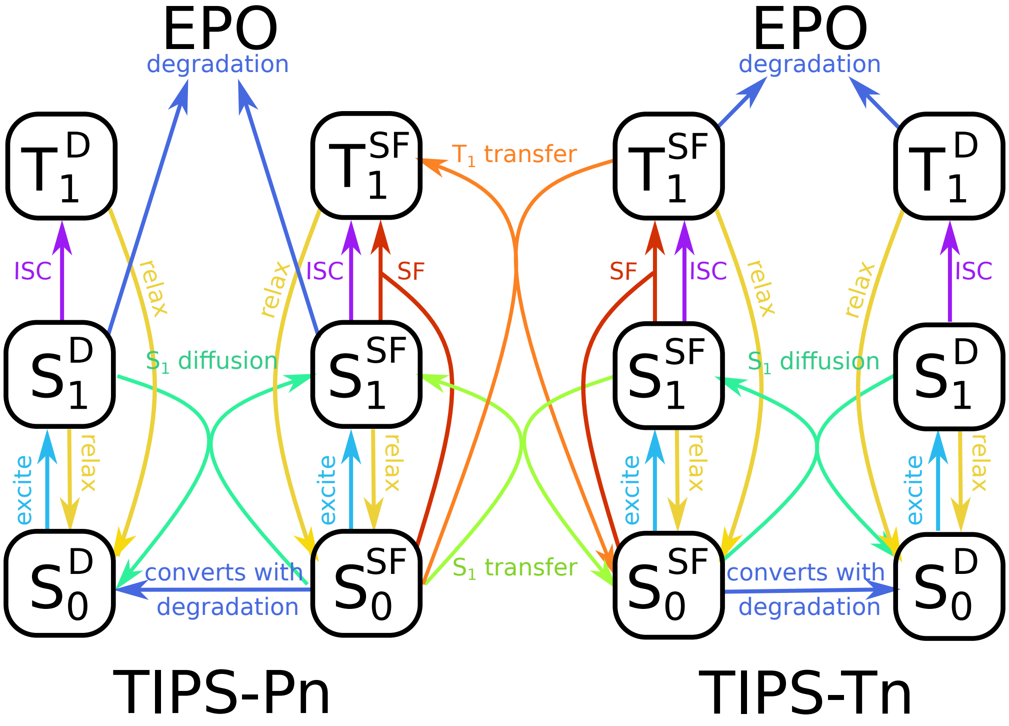

Figure S23: Kinetic model for the photodegradation of blend TIPS-Tn:TIPS-Pn NPs with diffusion. For clarity singlet and triplet energy transfer between TIPS-Tn and TIPS-Pn are only shown between SF site populations, but the model also includes transfer between diffusion sites. EPO represents the product endoperoxide(s).

With previously fitted photodegradation rate constants for TIPS-Tn and TIPS-Pn (in addition to known SF, relaxation, ISC, and excitation rate constants), we can now consider modeling the blend NPs, in which TIPS-Tn and TIPS-Pn are mixed together. In the blends two additional pathways become available: singlet-energy transfer (SET) from TIPS-Tn to TIPS-Pn with rate constant k_{SET}, and triplet-energy transfer (TET) from TIPS-Tn to TIPS-Pn with rate constant k_{TET}. The model for the blend NPs is shown in Figure S23, and can be described by the system of differential equations

$$\begin{aligned}\frac{\mathrm{d}[\mathrm{S}^{\mathrm{SF}}_{0,\mathrm{Tn}}]}{\mathrm{d}t} = &-k_{\mathrm{excite,Tn}}[\mathrm{S}^{\mathrm{SF}}_{0,\mathrm{Tn}}] + k_{\mathrm{S_1,Tn}}[\mathrm{S}^{\mathrm{SF}}_{1,\mathrm{Tn}}] + k_{\mathrm{T_1,Tn}}[\mathrm{T}^{\mathrm{SF}}_{1,\mathrm{Tn}}] - k_{\mathrm{D,Tn}}[\mathrm{S}^{\mathrm{D}}_{1,\mathrm{Tn}}][\mathrm{S}^{\mathrm{SF}}_{0,\mathrm{Tn}}] \\ &- k_{\mathrm{SF,Tn}}[\mathrm{S}^{\mathrm{SF}}_{0,\mathrm{Tn}}][\mathrm{S}^{\mathrm{SF}}_{1,\mathrm{Tn}}] - pk_{\mathrm{deg,Tn}}[\mathrm{T}^{\mathrm{SF}}_{1,\mathrm{Tn}}][\mathrm{S}^{\mathrm{SF}}_{0,\mathrm{Tn}}] + k_{\mathrm{TET}}[\mathrm{T}^{\mathrm{SF}}_{1,\mathrm{Tn}}]\left([\mathrm{S}^{\mathrm{D}}_{0,\mathrm{Pn}}] + [\mathrm{S}^{\mathrm{SF}}_{0,\mathrm{Pn}}]\right) \\ &+ k_{\mathrm{SET}}[\mathrm{S}^{\mathrm{SF}}_{1,\mathrm{Tn}}]\left([\mathrm{S}^{\mathrm{D}}_{0,\mathrm{Pn}}] + [\mathrm{S}^{\mathrm{SF}}_{0,\mathrm{Pn}}]\right),\end{aligned} \tag{S26a}$$

$$\begin{aligned}\frac{\mathrm{d}[\mathrm{S}^{\mathrm{D}}_{0,\mathrm{Tn}}]}{\mathrm{d}t} = &-k_{\mathrm{excite,Tn}}[\mathrm{S}^{\mathrm{D}}_{0,\mathrm{Tn}}] + k_{\mathrm{S_1,Tn}}[\mathrm{S}^{\mathrm{D}}_{1,\mathrm{Tn}}] + k_{\mathrm{T_1,Tn}}[\mathrm{T}^{\mathrm{D}}_{1,\mathrm{Tn}}] + k_{\mathrm{D,Tn}}[\mathrm{S}^{\mathrm{D}}_{1,\mathrm{Tn}}][\mathrm{S}^{\mathrm{SF}}_{0,\mathrm{Tn}}] \\ &+ pk_{\mathrm{deg,Tn}}[\mathrm{T}^{\mathrm{SF}}_{1,\mathrm{Tn}}][\mathrm{S}^{\mathrm{SF}}_{0,\mathrm{Tn}}] + k_{\mathrm{TET}}[\mathrm{T}^{\mathrm{D}}_{1,\mathrm{Tn}}]\left([\mathrm{S}^{\mathrm{D}}_{0,\mathrm{Pn}}] + [\mathrm{S}^{\mathrm{SF}}_{0,\mathrm{Pn}}]\right) \\ &+ k_{\mathrm{SET}}[\mathrm{S}^{\mathrm{D}}_{1,\mathrm{Tn}}]\left([\mathrm{S}^{\mathrm{D}}_{0,\mathrm{Pn}}] + [\mathrm{S}^{\mathrm{SF}}_{0,\mathrm{Pn}}]\right),\end{aligned} \tag{S26b}$$

$$\begin{aligned}\frac{\mathrm{d}[\mathrm{S}^{\mathrm{SF}}_{1,\mathrm{Tn}}]}{\mathrm{d}t} = &\, k_{\mathrm{excite,Tn}}[\mathrm{S}^{\mathrm{SF}}_{0,\mathrm{Tn}}] - k_{\mathrm{S_1,Tn}}[\mathrm{S}^{\mathrm{SF}}_{1,\mathrm{Tn}}] + k_{\mathrm{D,Tn}}[\mathrm{S}^{\mathrm{D}}_{1,\mathrm{Tn}}][\mathrm{S}^{\mathrm{SF}}_{0,\mathrm{Tn}}] - k_{\mathrm{ISC,Tn}}[\mathrm{S}^{\mathrm{SF}}_{1,\mathrm{Tn}}] \\ &- k_{\mathrm{SF,Tn}}[\mathrm{S}^{\mathrm{SF}}_{0,\mathrm{Tn}}][\mathrm{S}^{\mathrm{SF}}_{1,\mathrm{Tn}}] - k_{\mathrm{SET}}[\mathrm{S}^{\mathrm{SF}}_{1,\mathrm{Tn}}]\left([\mathrm{S}^{\mathrm{D}}_{0,\mathrm{Pn}}] + [\mathrm{S}^{\mathrm{SF}}_{0,\mathrm{Pn}}]\right),\end{aligned} \tag{S26c}$$

$$\frac{\mathrm{d}[\mathrm{S}^{\mathrm{D}}_{1,\mathrm{Tn}}]}{\mathrm{d}t} = k_{\mathrm{excite,Tn}}[\mathrm{S}^{\mathrm{D}}_{0,\mathrm{Tn}}] - k_{\mathrm{S_1,Tn}}[\mathrm{S}^{\mathrm{D}}_{1,\mathrm{Tn}}] - k_{\mathrm{D,Tn}}[\mathrm{S}^{\mathrm{D}}_{1,\mathrm{Tn}}][\mathrm{S}^{\mathrm{SF}}_{0,\mathrm{Tn}}] - k_{\mathrm{ISC,Tn}}[\mathrm{S}^{\mathrm{D}}_{1,\mathrm{Tn}}]$$

$$-k_{\mathrm{SET}}[\mathrm{S}^{\mathrm{D}}_{1,\mathrm{Tn}}]\left([\mathrm{S}^{\mathrm{D}}_{0,\mathrm{Pn}}]+[\mathrm{S}^{\mathrm{SF}}_{0,\mathrm{Pn}}]\right), \tag{S26d}$$

$$\frac{\mathrm{d}[\mathrm{T}^{\mathrm{SF}}_{1,\mathrm{Tn}}]}{\mathrm{d}t} = 2k_{\mathrm{SF,Tn}}[\mathrm{S}^{\mathrm{SF}}_{0,\mathrm{Tn}}][\mathrm{S}^{\mathrm{SF}}_{1,\mathrm{Tn}}] + k_{\mathrm{ISC,Tn}}[\mathrm{S}^{\mathrm{SF}}_{1,\mathrm{Tn}}] - k_{\mathrm{T_1,Tn}}[\mathrm{T}^{\mathrm{SF}}_{1,\mathrm{Tn}}] - k_{\mathrm{deg,Tn}}[\mathrm{T}^{\mathrm{SF}}_{1,\mathrm{Tn}}] - k_{\mathrm{TET}}[\mathrm{T}^{\mathrm{SF}}_{1,\mathrm{Tn}}]\left([\mathrm{S}^{\mathrm{D}}_{0,\mathrm{Pn}}]+[\mathrm{S}^{\mathrm{SF}}_{0,\mathrm{Pn}}]\right), \tag{S26e}$$

$$\frac{\mathrm{d}[\mathrm{T}^{\mathrm{D}}_{1,\mathrm{Tn}}]}{\mathrm{d}t} = k_{\mathrm{ISC,Tn}}[\mathrm{S}^{\mathrm{D}}_{1,\mathrm{Tn}}] - k_{\mathrm{T_1,Tn}}[\mathrm{T}^{\mathrm{D}}_{1,\mathrm{Tn}}] - k_{\mathrm{deg,Tn}}[\mathrm{T}^{\mathrm{D}}_{1,\mathrm{Tn}}] - k_{\mathrm{TET}}[\mathrm{T}^{\mathrm{D}}_{1,\mathrm{Tn}}]\left([\mathrm{S}^{\mathrm{D}}_{0,\mathrm{Pn}}]+[\mathrm{S}^{\mathrm{SF}}_{0,\mathrm{Pn}}]\right), \tag{S26f}$$

$$\frac{\mathrm{d}[\mathrm{S}^{\mathrm{SF}}_{0,\mathrm{Pn}}]}{\mathrm{d}t} = -k_{\mathrm{excite,Pn}}[\mathrm{S}^{\mathrm{SF}}_{0,\mathrm{Pn}}] + k_{\mathrm{S_1,Pn}}[\mathrm{S}^{\mathrm{SF}}_{1,\mathrm{Pn}}] + k_{\mathrm{T_1,Pn}}[\mathrm{T}^{\mathrm{SF}}_{1,\mathrm{Pn}}] - k_{\mathrm{D,Pn}}[\mathrm{S}^{\mathrm{D}}_{1,\mathrm{Pn}}][\mathrm{S}^{\mathrm{SF}}_{0,\mathrm{Pn}}] - k_{\mathrm{SF,Pn}}[\mathrm{S}^{\mathrm{SF}}_{0,\mathrm{Pn}}][\mathrm{S}^{\mathrm{SF}}_{1,\mathrm{Pn}}] - pk_{\mathrm{deg,Pn}}[\mathrm{S}^{\mathrm{SF}}_{1,\mathrm{Pn}}][\mathrm{S}^{\mathrm{SF}}_{0,\mathrm{Pn}}] - k_{\mathrm{TET}}\left([\mathrm{T}^{\mathrm{D}}_{1,\mathrm{Tn}}]+[\mathrm{T}^{\mathrm{SF}}_{1,\mathrm{Tn}}]\right)[\mathrm{S}^{\mathrm{SF}}_{0,\mathrm{Pn}}] + k_{\mathrm{SET}}\left([\mathrm{S}^{\mathrm{D}}_{1,\mathrm{Tn}}]+[\mathrm{S}^{\mathrm{SF}}_{1,\mathrm{Tn}}]\right)[\mathrm{S}^{\mathrm{SF}}_{0,\mathrm{Pn}}], \tag{S26g}$$

$$\frac{\mathrm{d}[\mathrm{S}^{\mathrm{D}}_{0,\mathrm{Pn}}]}{\mathrm{d}t} = -k_{\mathrm{excite,Pn}}[\mathrm{S}^{\mathrm{D}}_{0,\mathrm{Pn}}] + k_{\mathrm{S_1,Pn}}[\mathrm{S}^{\mathrm{D}}_{1,\mathrm{Pn}}] + k_{\mathrm{T_1,Pn}}[\mathrm{T}^{\mathrm{D}}_{1,\mathrm{Pn}}] + k_{\mathrm{D,Pn}}[\mathrm{S}^{\mathrm{D}}_{1,\mathrm{Pn}}][\mathrm{S}^{\mathrm{SF}}_{0,\mathrm{Pn}}] + pk_{\mathrm{deg,Pn}}[\mathrm{S}^{\mathrm{SF}}_{1,\mathrm{Pn}}][\mathrm{S}^{\mathrm{SF}}_{0,\mathrm{Pn}}] - k_{\mathrm{TET}}\left([\mathrm{T}^{\mathrm{D}}_{1,\mathrm{Tn}}]+[\mathrm{T}^{\mathrm{SF}}_{1,\mathrm{Tn}}]\right)[\mathrm{S}^{\mathrm{D}}_{0,\mathrm{Pn}}] - k_{\mathrm{SET}}\left([\mathrm{S}^{\mathrm{D}}_{1,\mathrm{Tn}}]+[\mathrm{S}^{\mathrm{SF}}_{1,\mathrm{Tn}}]\right)[\mathrm{S}^{\mathrm{D}}_{0,\mathrm{Pn}}], \tag{S26h}$$

$$\frac{\mathrm{d}[\mathrm{S}^{\mathrm{SF}}_{1,\mathrm{Pn}}]}{\mathrm{d}t} = k_{\mathrm{excite,Pn}}[\mathrm{S}^{\mathrm{SF}}_{0,\mathrm{Pn}}] - k_{\mathrm{S_1,Pn}}[\mathrm{S}^{\mathrm{SF}}_{1,\mathrm{Pn}}] + k_{\mathrm{D,Pn}}[\mathrm{S}^{\mathrm{D}}_{1,\mathrm{Pn}}][\mathrm{S}^{\mathrm{SF}}_{0,\mathrm{Pn}}] - k_{\mathrm{ISC,Pn}}[\mathrm{S}^{\mathrm{SF}}_{1,\mathrm{Pn}}] - k_{\mathrm{SF,Pn}}[\mathrm{S}^{\mathrm{SF}}_{0,\mathrm{Pn}}][\mathrm{S}^{\mathrm{SF}}_{1,\mathrm{Pn}}] - k_{\mathrm{deg,Pn}}[\mathrm{S}^{\mathrm{SF}}_{1,\mathrm{Pn}}] + k_{\mathrm{SET}}\left([\mathrm{S}^{\mathrm{D}}_{1,\mathrm{Tn}}]+[\mathrm{S}^{\mathrm{SF}}_{1,\mathrm{Tn}}]\right)[\mathrm{S}^{\mathrm{SF}}_{0,\mathrm{Pn}}], \tag{S26i}$$

$$\frac{\mathrm{d}[\mathrm{S}^{\mathrm{D}}_{1,\mathrm{Pn}}]}{\mathrm{d}t} = k_{\mathrm{excite,Pn}}[\mathrm{S}^{\mathrm{D}}_{0,\mathrm{Pn}}] - k_{\mathrm{S_1,Pn}}[\mathrm{S}^{\mathrm{D}}_{1,\mathrm{Pn}}] - k_{\mathrm{D,Pn}}[\mathrm{S}^{\mathrm{D}}_{1,\mathrm{Pn}}][\mathrm{S}^{\mathrm{SF}}_{0,\mathrm{Pn}}] - k_{\mathrm{ISC,Pn}}[\mathrm{S}^{\mathrm{D}}_{1,\mathrm{Pn}}] - k_{\mathrm{deg,Pn}}[\mathrm{S}^{\mathrm{D}}_{1,\mathrm{Pn}}] + k_{\mathrm{SET}}\left([\mathrm{S}^{\mathrm{D}}_{1,\mathrm{Tn}}]+[\mathrm{S}^{\mathrm{SF}}_{1,\mathrm{Tn}}]\right)[\mathrm{S}^{\mathrm{D}}_{0,\mathrm{Pn}}], \tag{S26j}$$

$$\frac{\mathrm{d}[\mathrm{T}^{\mathrm{SF}}_{1,\mathrm{Pn}}]}{\mathrm{d}t} = 2k_{\mathrm{SF,Pn}}[\mathrm{S}^{\mathrm{SF}}_{0,\mathrm{Pn}}][\mathrm{S}^{\mathrm{SF}}_{1,\mathrm{Pn}}] + k_{\mathrm{ISC,Pn}}[\mathrm{S}^{\mathrm{SF}}_{1,\mathrm{Pn}}] - k_{\mathrm{T_1,Pn}}[\mathrm{T}^{\mathrm{SF}}_{1,\mathrm{Pn}}] + k_{\mathrm{TET}}\left([\mathrm{T}^{\mathrm{D}}_{1,\mathrm{Tn}}]+[\mathrm{T}^{\mathrm{SF}}_{1,\mathrm{Tn}}]\right)[\mathrm{S}^{\mathrm{SF}}_{0,\mathrm{Pn}}] \tag{S26k}$$

$$\frac{\mathrm{d}[\mathrm{T}^{\mathrm{D}}_{1,\mathrm{Pn}}]}{\mathrm{d}t} = k_{\mathrm{ISC,Pn}}[\mathrm{S}^{\mathrm{D}}_{1,\mathrm{Pn}}] - k_{\mathrm{T_1,Pn}}[\mathrm{T}^{\mathrm{D}}_{1,\mathrm{Pn}}] + k_{\mathrm{TET}}\left([\mathrm{T}^{\mathrm{D}}_{1,\mathrm{Tn}}]+[\mathrm{T}^{\mathrm{SF}}_{1,\mathrm{Tn}}]\right)[\mathrm{S}^{\mathrm{D}}_{0,\mathrm{Pn}}]. \tag{S26l}$$

This system is considerably larger and more complicated than those for the neat NP systems, but as we discuss below, most of these parameters were constrained (as reported in Table S2) and only six were actually varied to fit the data. We initially only used one TIPS-Pn population, but for larger TIPS-Pn proportions (e.g. 1:5.7 and 1:10 TIPS-Tn:TIPS-Pn) two TIPS-Pn populations would be needed, as discussed below.

Table S2: Fixed rate constants used for modeling the photodegradation of blend NPs. Values were determined from neat systems, and errors are from an average over five and seven samples for TIPS-Tn and TIPS-Pn, respectively. Where errors are reported the average value was fixed for the blend NP systems.

	$k_{\mathrm{S_1}}$ ($\mathrm{s^{-1}}$)	$k_{\mathrm{T_1}}$ ($\mathrm{s^{-1}}$)	k_{SF} ($\mathrm{s^{-1}M^{-1}}$)	k_{ISC} * ($\mathrm{s^{-1}}$)	k_{deg} ($\mathrm{s^{-1}}$)	p ($\mathrm{M^{-1}}$)
TIPS-Tn	8.5×10^{7}	8.1×10^{7}	1.0×10^{11}	$(6\pm1)\times10^{7}$	$(4.8\pm0.3)\times10^{6}$	9000 ± 3000
TIPS-Pn	6.7×10^{7}	6.7×10^{7}	2.6×10^{11}	6.7×10^{7}	$(1.27\pm0.03)\times10^{7}$ †	0

* Rate of intersystem crossing plus the rate of triplet sensitization by oxygen.
† For 1:0.4 to 1:4.2 blend NPs. 1:5.7 and 1:10 NPs would also use a $7.2\times10^{7}\ \mathrm{s^{-1}}$ degradation rate constant.

In addition to the extra energy transfer pathways present in the blend NPs, the photophysics may also be altered by the dilution of TIPS-Tn chromophores by TIPS-Pn, and vice versa. This dilution will decrease the amount of SF sites in the NP, and increase the amount of diffusion sites. This dilution is analogous to the TIPS-Pn/polymethyl methacrylate (PMMA) blend NPs we have modeled previously, in which increasing the proportion of PMMA resulted in diffusion-limited SF, which we modeled by including fewer TIPS-Pn SF sites and more diffusion sites.[4] Therefore, we considered that the blend NPs are able to begin with a non-zero population of diffusion sites, which are then also able to increase over time as the NP photodegrades. We expect all rate constants (other than those for SET and TET from TIPS-Tn to TIPS-Pn) to be identical to those in the neat NP models, and that any changes in the efficiency of SF can be explained by the amount of diffusion in the NPs. The exception to this is the rate constant of diffusion itself: since diffusion was negligible in the neat NP modeling, we allowed the rate constant to vary for the blend NPs. Thus, only six parameters were fit to the data in the blend NP modeling: the rate constants of SET and TET from TIPS-Tn to TIPS-Pn, k_{SET} and k_{TET}, the rate constant of diffusion within the neat materials, $k_{\mathrm{D,Pn}}$ and $k_{\mathrm{D,Tn}}$, and the initial proportions of diffusion sites, $[\mathrm{S}^{\mathrm{D}}_{0,\mathrm{Pn}}](0)/\left([\mathrm{S}^{\mathrm{D}}_{0,\mathrm{Pn}}](0)+[\mathrm{S}^{\mathrm{SF}}_{0,\mathrm{Pn}}](0)\right)$ and $[\mathrm{S}^{\mathrm{D}}_{0,\mathrm{Tn}}](0)/\left([\mathrm{S}^{\mathrm{D}}_{0,\mathrm{Tn}}](0)+[\mathrm{S}^{\mathrm{SF}}_{0,\mathrm{Tn}}](0)\right)$.

As the proportion of TIPS-Pn in the blend NPs is increased, two trends may arise. One, SF in TIPS-Tn becomes less efficient as the TIPS-Tn becomes diluted by TIPS-Pn, whilst SF in TIPS-Pn becomes more efficient. Two, energy transfer from TIPS-Tn to TIPS-Pn becomes more efficient. Both of these trends will lead to slower TIPS-Tn degradation as the proportion of TIPS-Pn increases. The question arises of whether the model is sensitive enough to distinguish between the two. To test the sensitivity, we initially fit the model with energy transfer processes from TIPS-Tn to TIPS-Pn turned off, i.e. $k_{\mathrm{SET}} = k_{\mathrm{TET}} = 0$, to see if diffusion-limited SF (involving only molecules of the same type) alone could explain the experimental data. The resultant fit is shown in Figure S24 for the 1:0.4 and 1:1.1 NPs as an example. The fits are poor, so it is clear that diffusion-limited SF cannot solely explain the photodegradation.

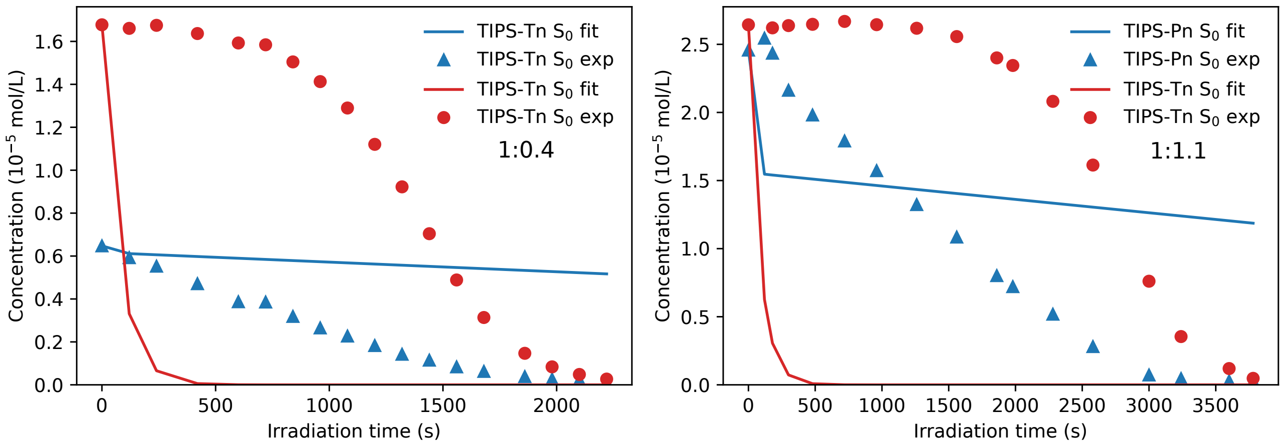

Figure S24: Fit of the blend model to the photodegradation of a sample of 1:0.4 and 1:1 TIPS-Pn:TIPS-Tn NPs, with no singlet or triplet energy transfer from TIPS-Tn to TIPS-Pn ($k_{\mathrm{SET}} = k_{\mathrm{TET}} = 0$).

We next tested if SET from TIPS-Tn to TIPS-Tn alone could explain the trends in photodegradation, and repeated the fits with k_{TET} fixed to zero, and k_{SET} allowed to vary. The resultant fits are in Figure S25. The fits, particularly for TIPS-Tn at low TIPS-Pn mass ratio are poor, indicating SET alone cannot fit the data, and TET is necessary. Similarly, Figure S26 shows fits with TET only, and k_{SET} fixed to zero. These fits are better than the SET only fits, but are still significantly worse than when both processes are included, as discussed next.

Finally, Figure S27 shows fits in which all processes are included (diffusion, SET, and TET). These fits are much better than when k_{SET} and/or k_{TET} are constrained to zero, indicating that both processes

are necessary to describe the data. The RMSE of each blend NP sample ($\mathrm{RMSE_b}$) was calculated as

$$\mathrm{RMSE_b} = \sqrt{\frac{1}{2N}\sum_{j=1}^{N}\left(\frac{(\mathrm{fit}_{\mathrm{Pn},j} - \mathrm{expt}_{\mathrm{Pn},j})^2}{\mathrm{expt}_{\mathrm{Pn},1}^{2}} + \frac{(\mathrm{fit}_{\mathrm{Tn},j} - \mathrm{expt}_{\mathrm{Tn},j})^2}{\mathrm{expt}_{\mathrm{Tn},1}^{2}}\right)} \tag{S27}$$

where N is the number of data points in the data set, $\mathrm{fit}_{\mathrm{Tn/Pn},j}$ and $\mathrm{expt}_{\mathrm{Tn/Pn},j}$ are the fitted and experimental concentrations of TIPS-Tn/TIPS-Pn at point j, respectively, and $\mathrm{expt}_{\mathrm{Tn/Pn},1}$ is the experimental concentration of TIPS-Tn/TIPS-Pn at the initial data point (i.e. time zero). The $\mathrm{RMSE_b}$ are shown in Table S3 for each variation of the blend model. For every sample the error is lowest for the case that both SET and TET are included. Additionally, the average $\mathrm{RMSE_b}$ across all samples is lowest for non-zero SET and TET.

Table S3: Root mean squared error ($\mathrm{RMSE_b}$, Equation S27) of fits of different models to the blend NP photodegradation data.

TIPS-Tn:TIPS-Pn	Proportion of TIPS-Pn	No energy transfer ($k_{\mathrm{TET}} = k_{\mathrm{SET}} = 0$)	SET only ($k_{\mathrm{TET}} = 0$)	TET only ($k_{\mathrm{SET}} = 0$)	SET and TET
1:0.4[a]	0.27	0.476	0.076	0.073	0.031
1:0.4[b]	0.28	0.432	0.055	0.023	0.016
1:0.5	0.31	0.431	0.099	0.048	0.028
1:0.9[c]	0.44	0.535	0.081	0.035	0.029
1:0.9[d]	0.45	0.450	0.087	0.024	0.021
1:1.1	0.48	0.556	0.068	0.043	0.034
1:2.1	0.65	0.501	0.059	0.047	0.037
1:4.2	0.79	0.494	0.042	0.031	0.023
Average		0.484	0.071	0.040	0.027

[a] Fit to sample with an initial TIPS-Tn concentration of 30 µM.
[b] Fit to sample with an initial TIPS-Tn concentration of 17 µM.
[c] Fit to sample with an initial TIPS-Tn concentration of 23 µM.
[d] Fit to sample with an initial TIPS-Tn concentration of 26 µM.

The fitted time constants are reported in Table S4 and the corresponding second-order rate constants in Table S5. Notably, the rate constants of SET and TET consistently and sensitively fit to non-zero values, again indicating that both processes are active. The rate constant of TIPS-Tn diffusion, $k_{\mathrm{D,Tn}}$, does not fit as sensitively, and occasionally cross zero, indicating that this process is negligible, as with the diffusion sites in neat TIPS-Tn NPs. Figures S29 and S28 show the individual contributions of SF and diffusion sites in TIPS-Tn and TIPS-Pn, respectively. Finally, Figures S30 and S31 show the proportions of each process for each population, and how they change over time as the NP degrades and the concentration of molecules decreases.

Fitting was only performed for blend ratios up to 1:4.2 TIPS-Tn:TIPS-Pn. For these samples, only one TIPS-Pn degradation rate constant was used (the slower rate constant that corresponded to a larger portion of the photodegradation in the neat TIPS-Pn NPs), which results in a good fit for these blends. Blending with TIPS-Tn likely makes the TIPS-Pn more disordered, and differences in packing or arrangements of molecules are less significant, so only one photodegradation rate constant is needed. However the TIPS-Pn photodegradation for 1:5.7 and 1:10 NPs begins to behave similarly to neat TIPS-Pn NPs, with two distinct slopes, suggesting the need for multiple TIPS-Pn photodegradation rate constants. However, including the second rate-constant population would result in reduced sensitivity of these fits, and the trends in rate constants between these samples and NPs with lower proportions of TIPS-Pn would not necessarily be meaningful. Hence we did not attempt to model the data for these samples in

detail for now.

Table S4: Parameters fit to the blend NP photodegradation data, including proportions of diffusion sites, time constants of diffusion (at time zero), and time constants of triplet and singlet energy transfer (at time zero).$^{\gamma}$ Errors were calculated as 1.645 times the standard error of the fit (i.e a 90% confidence interval).

TIPS-Tn:TIPS-Pn	Proportion of TIPS-Pn	Proportion of $[S^{D}_{0,Tn}](0)^{\dagger}$	Proportion of $[S^{D}_{0,Pn}](0)^{\delta}$	$\tau_{D,Tn}$ (ps)	$\tau_{D,Pn}$ (ps)	τ_{SET} (ps)$^{\gamma}$	τ_{TET} (ps)$^{\gamma}$
1:0.4^{a}	0.27	0.0±0.3	0.75±0.03	220±60	24.2±2	1.180±0.001	160±90
1:0.4^{b}	0.28	0.00±0.09	0.704±0.009	2000±2000	20±3	0.6±0.1	271±80
1:0.5	0.31	0.01±0.03	0.691±0.001	100±30	18.7±0.4	0.4±0.2	300±100
1:0.9^{c}	0.44	0.0±0.6	0.66±0.01	20±6	17±2	0.6±0.3	100±50
1:0.9^{d}	0.45	0±0.08	0.653±0.007	350±20	22±2	0.65±0.05	130±10
1:1.1	0.48	0±0.03	0.685±0.008	200±300	18±1	0.50±0.04	106±2
1:2.1	0.65	0.00±0.05	0.584±0.004	70±9	13±1	0.5±0.1	120±20
1:4.2	0.79	0.08±0.04	0.665±0.007	292±9	13.0±0.6	0.111±0.003	450±40

^{a} Fit to sample with an initial TIPS-Tn concentration of 30 µM.
^{b} Fit to sample with an initial TIPS-Tn concentration of 17 µM.
^{c} Fit to sample with an initial TIPS-Tn concentration of 23 µM.
^{d} Fit to sample with an initial TIPS-Tn concentration of 26 µM.

$\dagger$ $\frac{[S^{D}_{0,Tn}](0)}{[S^{D}_{0,Tn}](0)+[S^{SF}_{0,Tn}](0)}$

δ $\frac{[S^{D}_{0,Pn}](0)}{[S^{D}_{0,Pn}](0)+[S^{SF}_{0,Pn}](0)}$

$^{\gamma}\tau_{SET/TET} = 1/(k_{SET/TET} \times [S_{0,Pn}](0))$ and $\tau_{D,Tn/Pn} = 1/(k_{D,Tn/Pn} \times [S^{SF}_{0,Tn/Pn}](0))$

Table S5: Second-order rate constants fit to the blend NP photodegradation data, including rate constants of diffusion for TIPS-Pn and TIPS-Tn singlet excitons, and rates of singlet and triplet energy transfer. Errors were calculated as 1.645 times the standard error of the fit (i.e a 90% confidence interval).

TIPS-Tn:TIPS-Pn	Proportion of TIPS-Pn	$k_{\mathrm{D,Tn}}$ ($\times10^{14}$ $\mathrm{s^{-1}M^{-1}}$)	$k_{\mathrm{D,Pn}}$ ($\times10^{15}$ $\mathrm{s^{-1}M^{-1}}$)	k_{SET} ($\times10^{16}$ $\mathrm{s^{-1}M^{-1}}$)	k_{TET} ($\times10^{14}$ $\mathrm{s^{-1}M^{-1}}$)
1:0.4[a]	0.27	1.4±0.4	15±2	7.56±0.01	6±3
1:0.4[b]	0.28	0.3±0.3	25±4	26±7	6±2
1:0.5	0.31	7±2	27.8±0.6	40±20	6±2
1:0.9[c]	0.44	2.2±0.6	10±1	9±4	5±2
1:0.9[d]	0.45	1.11±0.06	6.3±0.7	7.3±0.6	3.6±0.4
1:1.1	0.48	2±2	7.4±0.4	8±0.6	3.85±0.08
1:2.1	0.65	15±700	9.5±0.4	10±1	4.4±0.6
1:4.2	0.79	4±2	7.0±0.4	2.74±0.08	0.68±0.06
Average[e]		7±3	14±3	17±5	4.4±0.6

[a] Fit to sample with an initial TIPS-Tn concentration of 30 µM.
[b] Fit to sample with an initial TIPS-Tn concentration of 17 µM.
[c] Fit to sample with an initial TIPS-Tn concentration of 23 µM.
[d] Fit to sample with an initial TIPS-Tn concentration of 26 µM.
[e] Errors are the standard error of the average values.

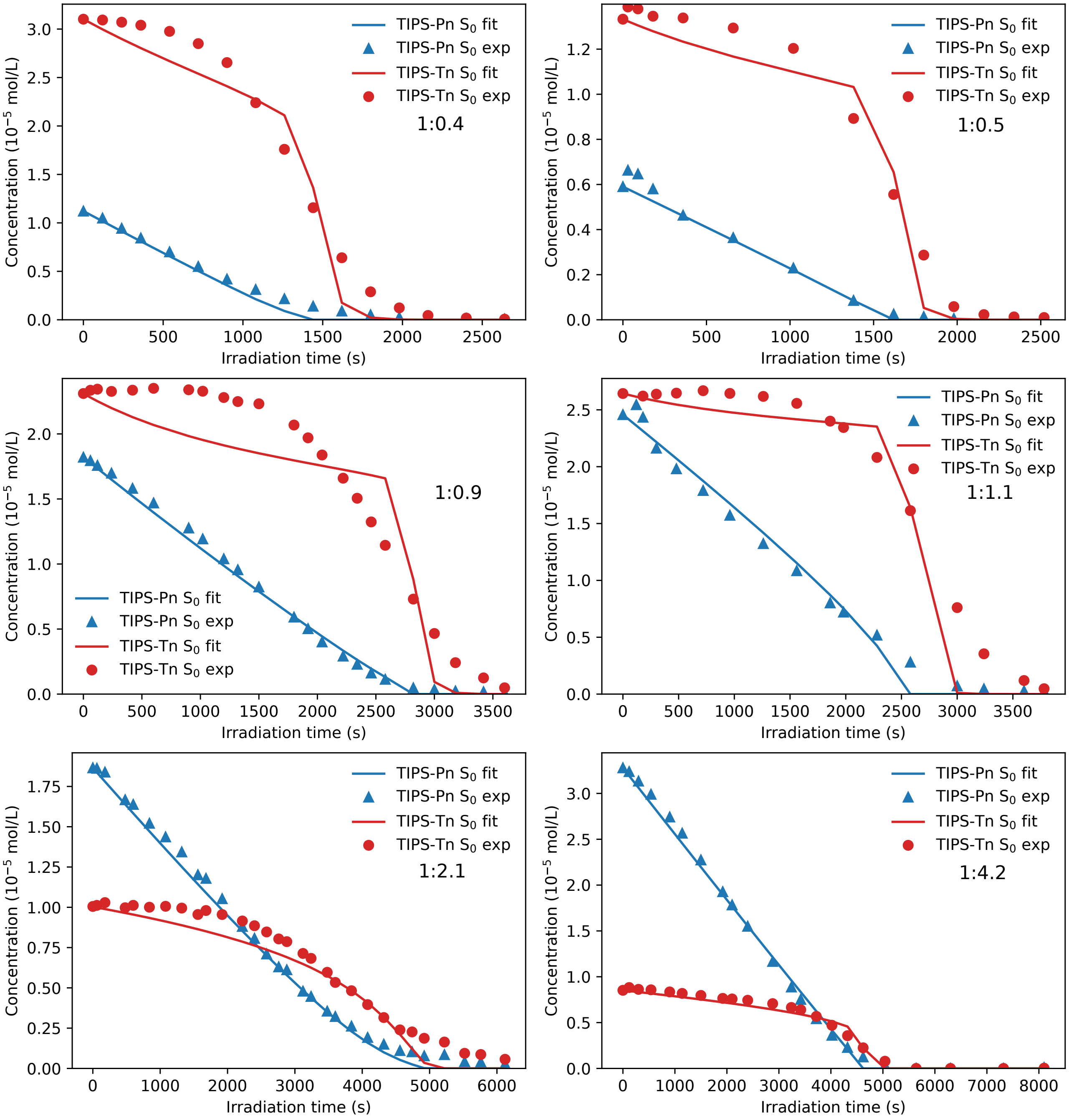

Figure S25: Fit of the blend model to the photodegradation of TIPS-Tn:TIPS-Pn NPs, with SET, but no TET ($k_{\mathrm{TET}} = 0$) from TIPS-Tn to TIPS-Pn.

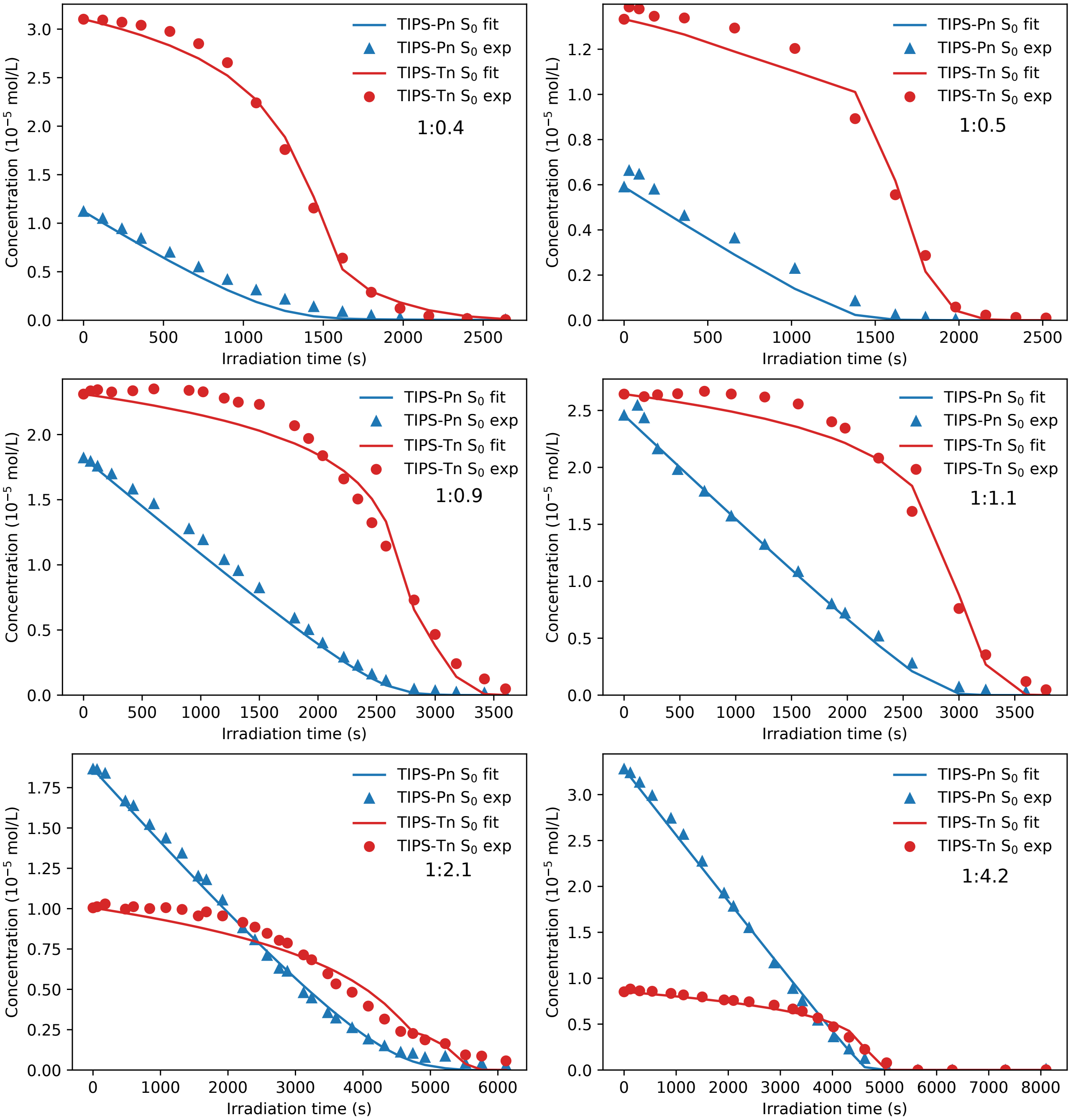

Figure S26: Fit of the blend model to the photodegradation of TIPS-Tn:TIPS-Pn NPs, with TET, but no SET ($k_{\mathrm{SET}} = 0$) from TIPS-Tn to TIPS-Pn.

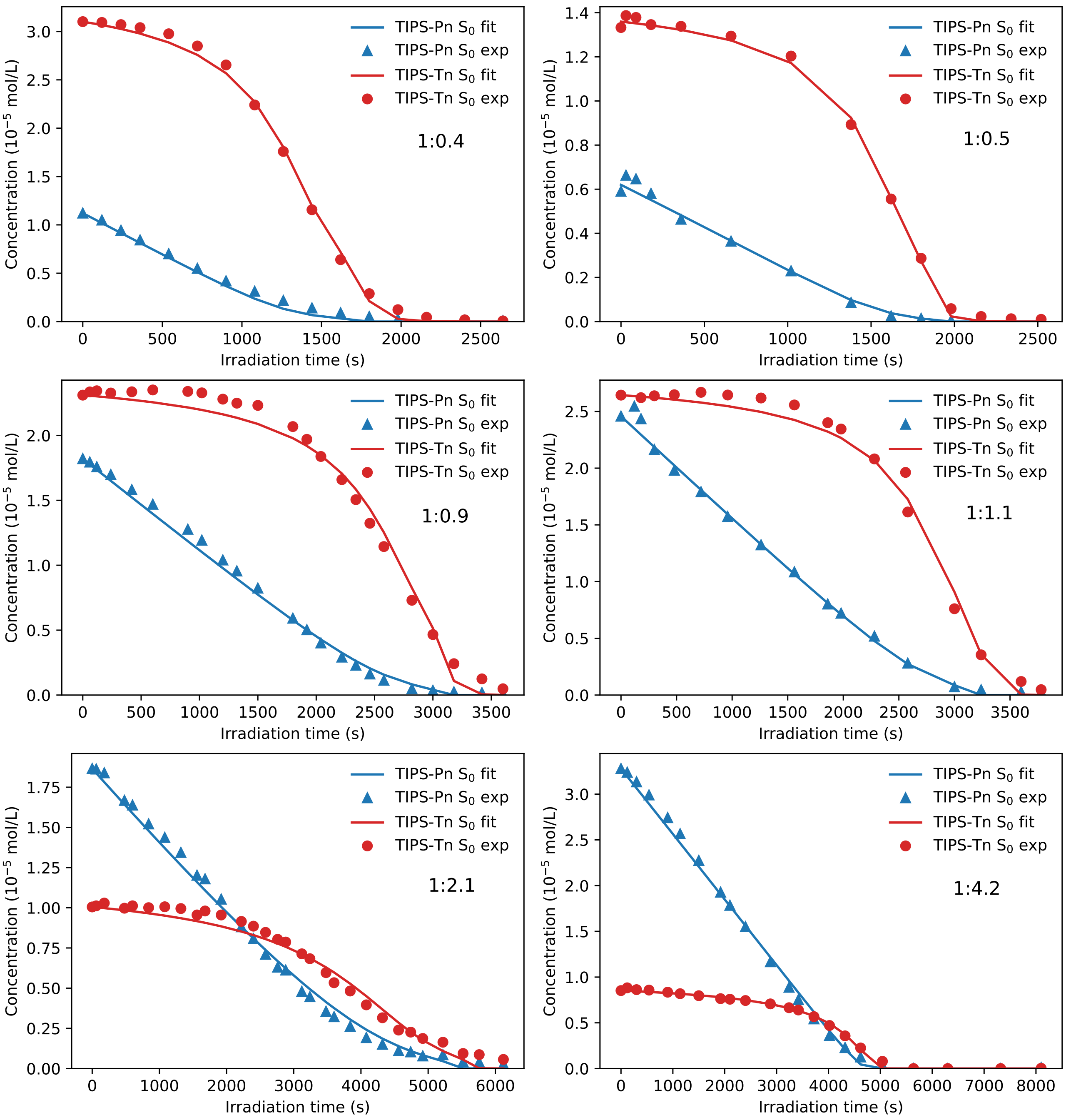

Figure S27: Fit of the blend model to the photodegradation of TIPS-Tn:TIPS-Pn NPs, with the addition of SET and TET from TIPS-Tn to TIPS-Pn.

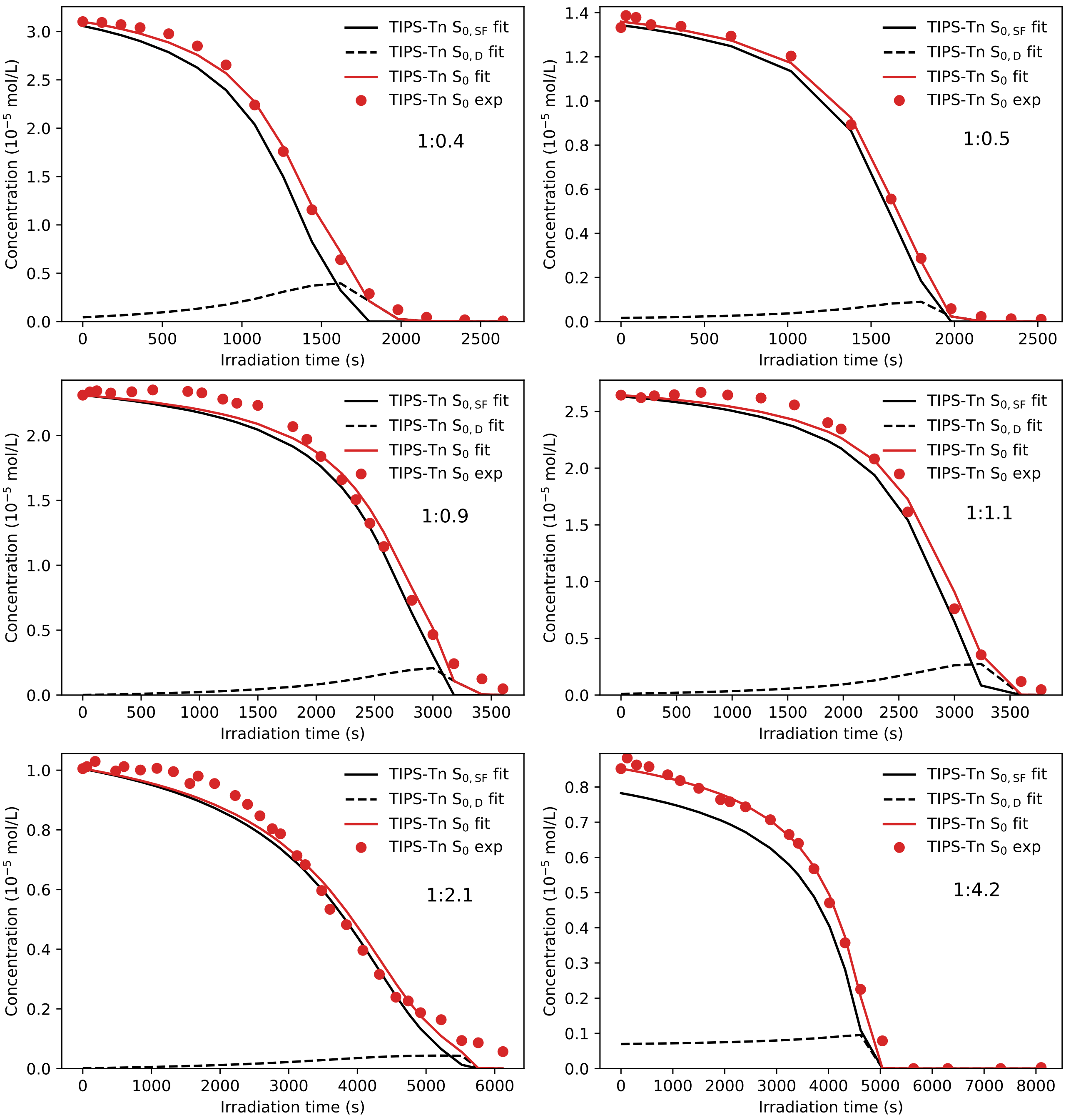

Figure S28: TIPS-Tn component of the fit of the blend model to the photodegradation of TIPS-Tn:TIPS-Pn NPs, with the addition of SET and TET from TIPS-Tn to TIPS-Pn. Contributions from the SF site population and the diffusion site population are shown as solid and dashed black lines, respectively.

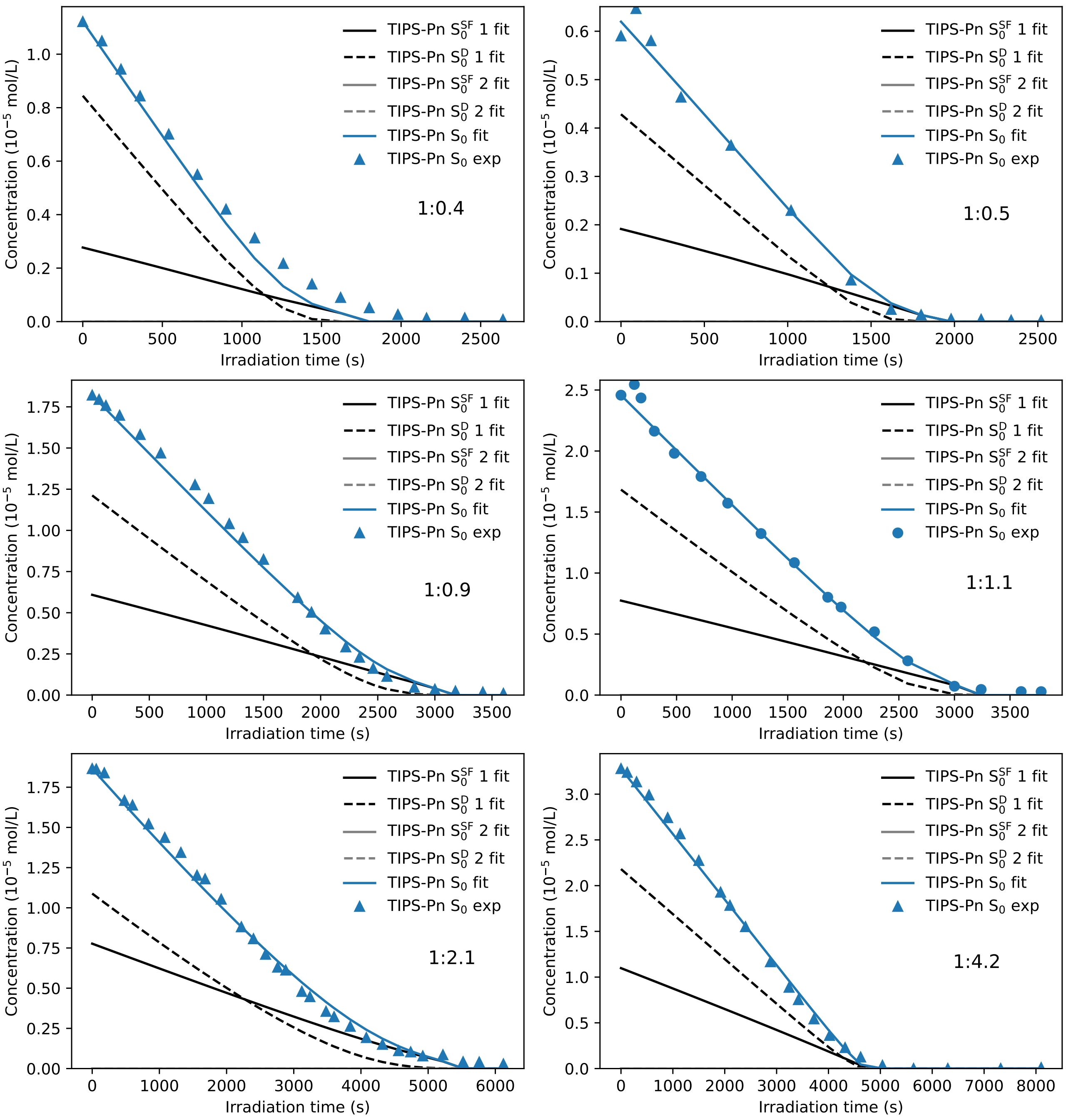

Figure S29: TIPS-Pn component of the fit of the blend model to the photodegradation of TIPS-Tn:TIPS-Pn NPs, with the addition of SET and TET. "1" populations correspond to sites with a TIPS-Pn photodegradation rate constant of 1.27×10^7 s^{-1}, and "2" correspond to a photodegradation rate of 7×10^7 s^{-1} (only used for 1:5.7 and 1:10 blend NPs). Contributions from SF site populations and diffusion site populations are shown as solid and dashed black or gray lines, respectively

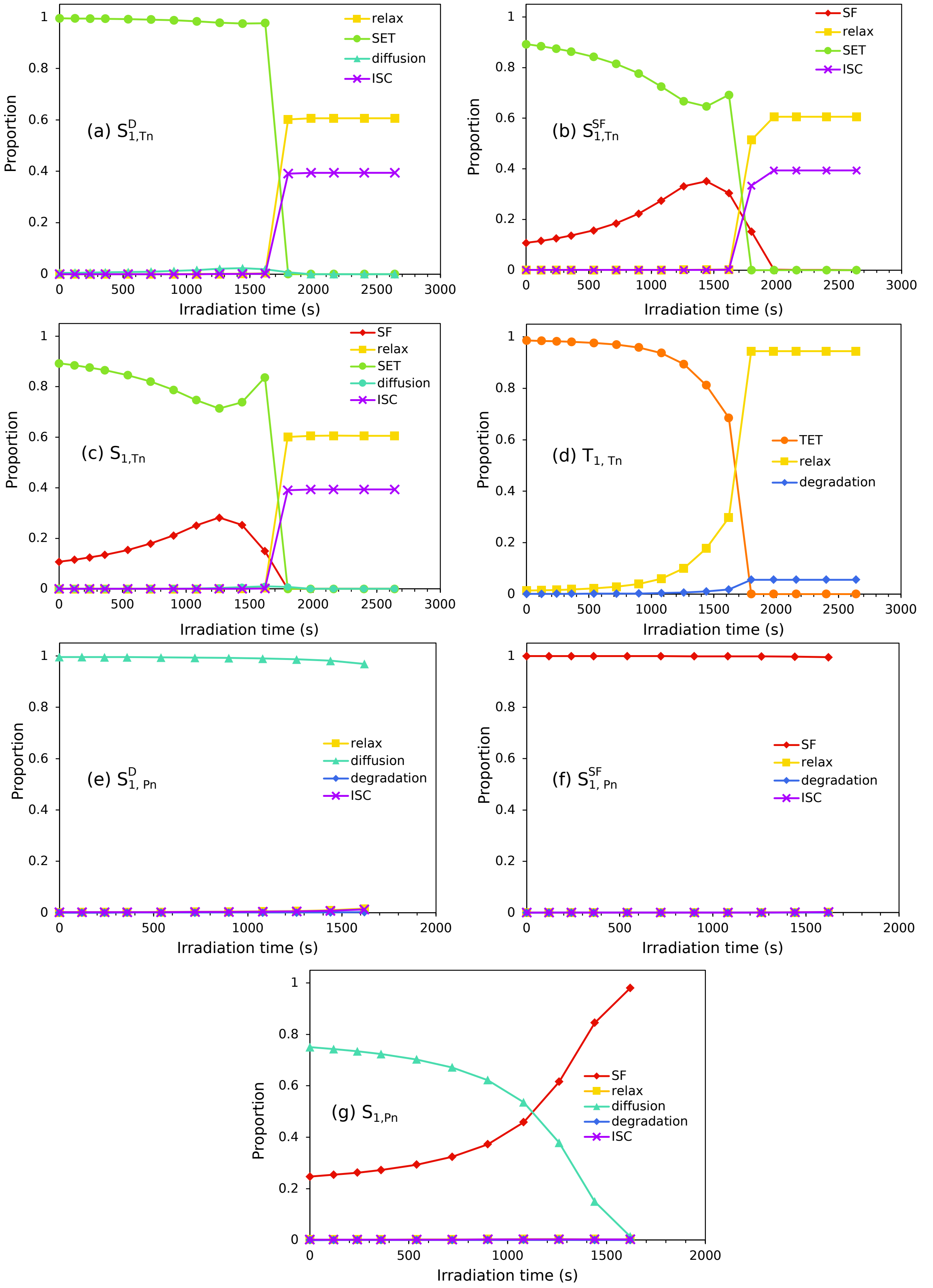

Figure S30: Proportions of each process undergone by different excited states for 1:0.4 blend NPs, as a function of irradiation time.

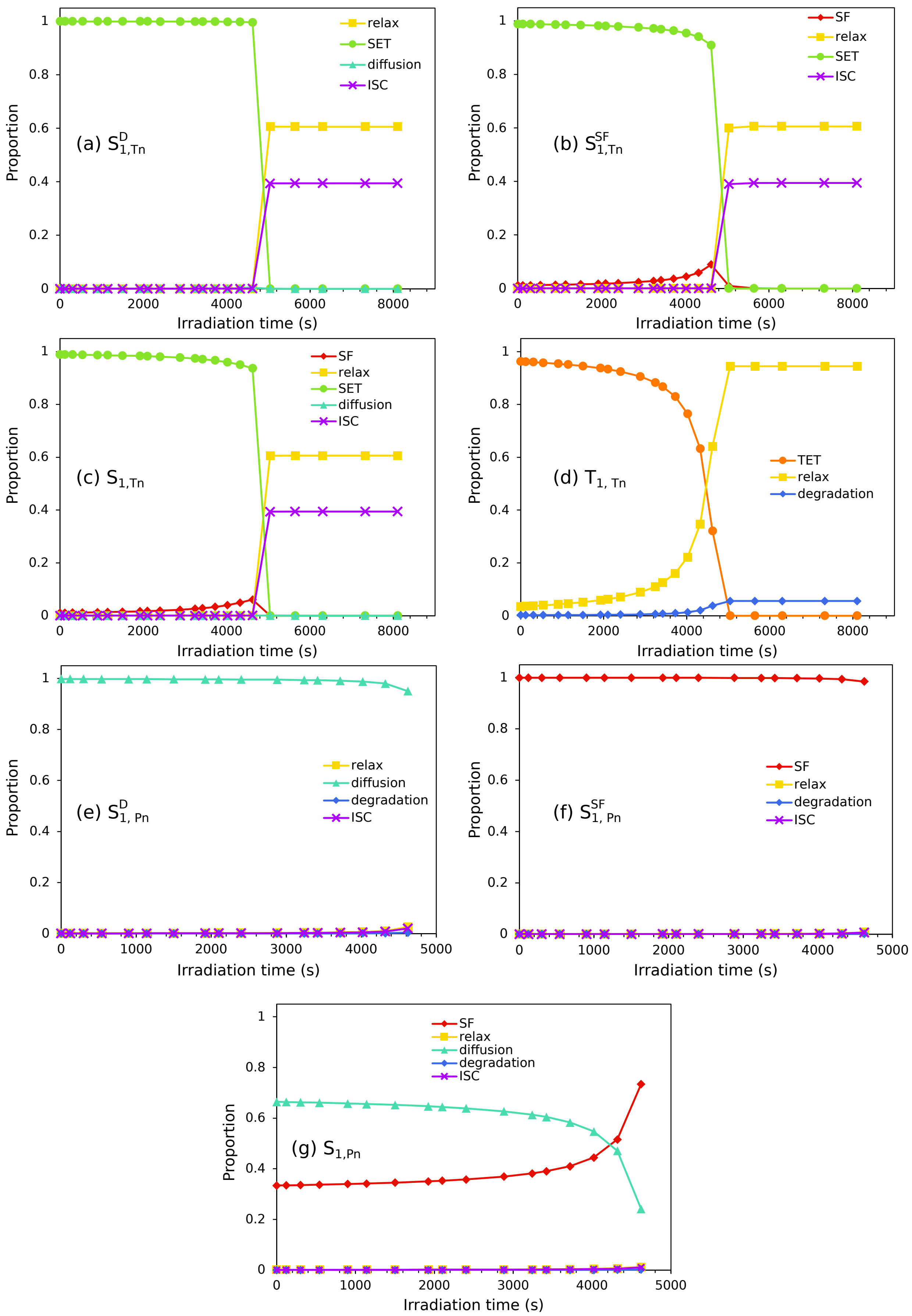

Figure S31: Proportions of each process undergone by different excited states for 1:4 blend NPs, as a function of irradiation time.

References

(1) Hudson, R. J.; Huang, D. M.; Kee, T. W. Anisotropic Triplet Exciton Diffusion in Crystalline Functionalized Pentacene. *J. Phys. Chem. C* **2020**, *124*, 23541–23550.

(2) Tayebjee, M. J. Y.; Schwarz, K. N.; MacQueen, R. W.; Dvorak, M.; Lam, A. W. C.; Ghiggino, K. P.; McCamey, D. R.; Schmidt, T. W.; Conibeer, G. J. Morphological Evolution and Singlet Fission in Aqueous Suspensions of TIPS-Pentacene Nanoparticles. *J. Phys. Chem. C* **2016**, *120*, 157–165.

(3) Pensack, R. D.; Grieco, C.; Purdum, G. E.; Mazza, S. M.; Tilley, A. J.; Ostroumov, E. E.; Seferos, D. S.; Loo, Y.-L.; Asbury, J. B.; Anthony, J. E. et al. Solution-processable, Crystalline Material for Quantitative Singlet Fission. *Mater. Horiz.* **2017**, *4*, 915–923.

(4) Stuart, A.; Tapping, P. C.; Schrefl, E.; Huang, D. M.; Kee, T. W. Controlling the Efficiency of Singlet Fission in TIPS-Pentacene/Polymer Composite Nanoparticles. *J. Phys. Chem. C* **2019**, *123*, 5813–5825.

(5) Hudson, R. J.; Stuart, A. N.; de la Perrelle, J. M.; Huang, D. M.; Kee, T. W. Nanoparticle Size-Dependent Singlet Fission and Exciton Dynamics in Amorphous TIPS-Pentacene. *J. Phys. Chem. C* **2021**, *125*, 21559–21570.

(6) Burdett, J. J.; Bardeen, C. J. Quantum Beats in Crystalline Tetracene Delayed Fluorescence Due to Triplet Pair Coherences Produced by Direct Singlet Fission. *J. Am. Chem. Soc.* **2012**, *134*, 8597–8607.

(7) Zeiser, C.; Moretti, L.; Lepple, D.; Cerullo, G.; Maiuri, M.; Broch, K. Singlet Heterofission in Tetracene–Pentacene Thin-Film Blends. *Angew. Chem. Int. Ed.* **2020**, *59*, 19966–19973.

(8) Hudson, R. J.; de la Perrelle, J. M.; Pensack, R. D.; Kudisch, B.; Scholes, G. D.; Huang, D. M.; Kee, T. W. Organizing Crystalline Functionalized Pentacene Using Periodicity of Poly(Vinyl Alcohol). *J. Phys. Chem. Lett.* **2020**, *11*, 516–523.

(9) Kang, S.; Yoon, T. W.; Kim, G.-Y.; Kang, B. Review of Conjugated Polymer Nanoparticles: From Formulation to Applications. *ACS Appl. Nano Mater.* **2022**, *5*, 17436–17460.

(10) Schwarz, K. N.; Farley, S. B.; Smith, T. A.; Ghiggino, K. P. Charge generation and morphology in P3HT:PCBM nanoparticles prepared by mini-emulsion and reprecipitation methods. *Nanoscale* **2015**, *7*, 19899–19904.

(11) Stuart, A. N.; Kee, T. W.; Huang, D. M. Role of Singlet and Triplet Excited States in the Oxygen-Mediated Photophysics and Photodegradation of Polyacenes. *J. Am. Chem. Soc.* **2024**, *146*, 2174–2186.

(12) Brasseur, G. P.; Jacob, D. J. *Modeling of Atmospheric Chemistry*; Cambridge University Press, 2017; p 253–274.

(13) Virtanen, P.; Gommers, R.; Oliphant, T. E.; Haberland, M.; Reddy, T.; Cournapeau, D.; Burovski, E.; Peterson, P.; Weckesser, W.; Bright, J. et al. SciPy 1.0: Fundamental Algorithms for Scientific Computing in Python. *Nature Methods* **2020**, *17*, 261–272.

(14) Hairer, E.; Wanner, G. Stiff differential equations solved by Radau methods. *J. Comput. Appl. Math.* **1999**, *111*, 93–111.

(15) Newville, M.; Otten, R.; Nelson, A.; Stensitzki, T.; Ingargiola, A.; Allan, D.; Fox, A.; Carter, F.; Michał,; Osborn, R. et al. lmfit/lmfit-py: 1.2.2. 2023; https://doi.org/10.5281/zenodo.8145703.

(16) Nelder, J. A.; Mead, R. A Simplex Method for Function Minimization. *Comput. J.* **1965**, *7*, 308–313.

(17) Roberts, S. T.; McAnally, R. E.; Mastron, J. N.; Webber, D. H.; Whited, M. T.; Brutchey, R. L.; Thompson, M. E.; Bradforth, S. E. Efficient Singlet Fission Discovered in a Disordered Acene Film. *J. Am. Chem. Soc.* **2012**, *134*, 6388–6400.

(18) de la Perrelle, J. M.; Tapping, P. C.; Schrefl, E.; Stuart, A. N.; Huang, D. M.; Kee, T. W. Singlet Fission Preserves Polarisation Correlation of Excitons. *Phys. Chem. Chem. Phys.* **2023**, *25*, 6817–6829.

(19) Dover, C. B.; Gallaher, J. K.; Frazer, L.; Tapping, P. C.; Petty II, A. J.; Crossley, M. J.; Anthony, J. E.; Kee, T. W.; Schmidt, T. W. Endothermic Singlet Fission is Hindered by Excimer Formation. *Nat. Chem.* **2018**, *10*, 305–310.

(20) Hudson, R. J.; Stuart, A. N.; Huang, D. M.; Kee, T. W. What Next for Singlet Fission in Photovoltaics? The Fate of Triplet and Triplet-Pair Excitons. *J. Phys. Chem. C* **2022**, *126*, 5369–5377.

(21) Stuart, A. N.; Tapping, P. C.; Kee, T. W.; Huang, D. M. Pitfalls of Quantifying Intersystem Crossing Rates in Singlet-fission Chromophore Solutions. *J. Chem. Phys.* **2022**, *157*, 084312.
